# Molecular bowls for inclusion complexation of toxic anticancer drug methotrexate†

**DOI:** 10.1039/d3sc05627a

**Published:** 2024-05-28

**Authors:** Pratik Karmakar, Tyler J. Finnegan, Darian C. Rostam, Sagarika Taneja, Sefa Uçar, Alexandar L. Hansen, Curtis E. Moore, Christopher M. Hadad, Kornkanya Pratumyot, Jon R. Parquette, Jovica D. Badjić

**Affiliations:** a Department of Chemistry and Biochemistry, The Ohio State University 100 West 18^th^ Avenue Columbus Ohio 43210 USA badjic.1@osu.edu; b Supramolecular Chemistry Research Unit, Department of Chemistry, Faculty of Science, King Mongkut's University of Technology Thonburi 126 Pracha Uthit Road, Bang Mod, Thung Khru Bangkok 10140 Thailand; c Atatürk University, Faculty of Science, Department of Chemistry Erzurum 25240 Turkey; d Campus Chemical Instrumentation Center, The Ohio State University 100 West 18^th^ Avenue Columbus Ohio 43210 USA

## Abstract

We describe the preparation and study of novel cavitands, molecular bowls 1^6+^ and 2^6+^, as good binders of the anticancer drug methotrexate (MTX). Molecular bowls are comprised of a curved tribenzotriquinacene (TBTQ) core conjugated to three macrocyclic pyridinium units at the top. The cavitands are easily accessible *via* two synthetic steps from hexabromo-tribenzotriquinacene in 25% yield. As amphiphilic molecules, bowls 1^6+^ and 2^6+^ self-associate in water by the nucleation-to-aggregation pathway (NMR). The bowls are preorganized, having a semi-rigid framework comprising a fixed bottom with a wobbling pyridinium rim (VT NMR and MD). Further studies, both experimental (NMR) and computational (DFT and MCMM), suggested that a folded MTX occupies the cavity of bowls wherein it forms π–π, C–H–π, and ion pairing intermolecular contacts but also undergoes desolvation to give stable binary complexes (μM) in water. Moreover, a computational protocol is introduced to identify docking pose(s) of MTX inside molecular bowls from NMR shielding data. Both molecular bowls have shown *in vitro* biocompatibility with liver and kidney cell lines (MTS assay). As bowl 2^6+^ is the strongest binder of MTX reported to date, we envision it as an excellent candidate for further studies on the way toward developing an antidote capable of removing MTX from overdosed cancer patients.

## Abstract 2

We describe synthesis, conformational dynamics and assembly characteristics of novel and tribenzotriquinacene-based hosts in water. These biocompatible molecules form stable inclusion complexes with toxic and anticancer drug methotrexate.

## Graphical abstract

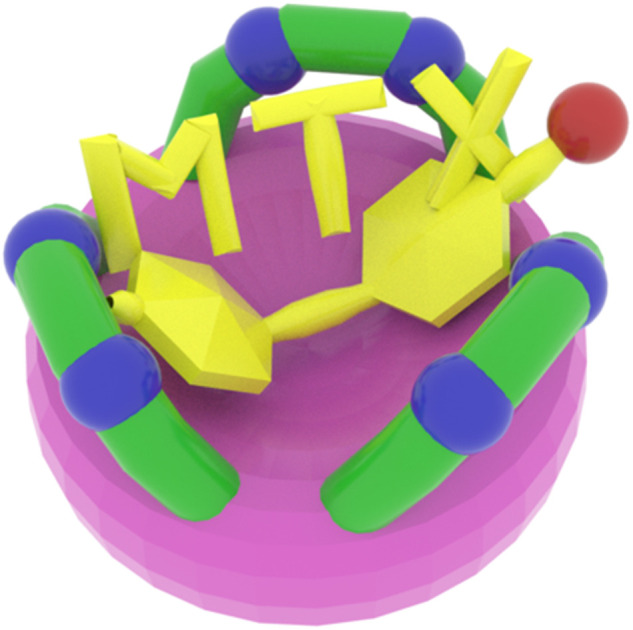

## Introduction

**Fig. 1:**
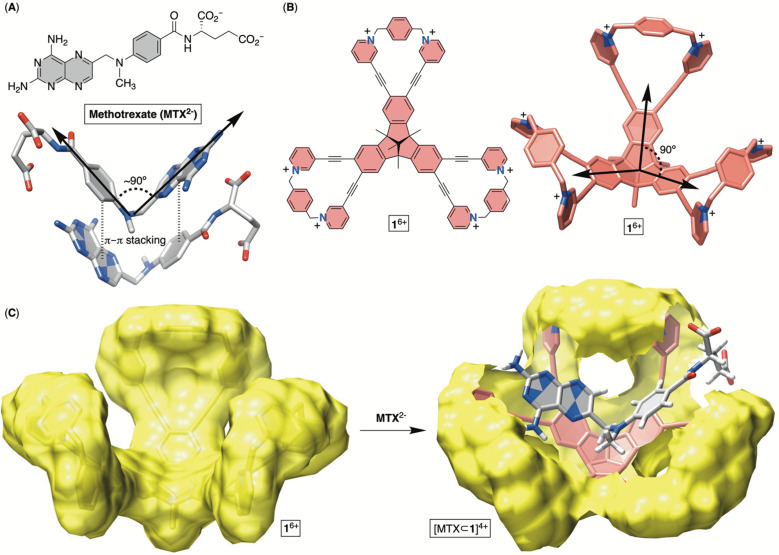
(A) Chemical structure of anticancer drug methotrexate (MTX^2−^) with a stick representation of its folded form in the solid state. (B) Chemical structure of bowl 1^6+^ with its stick representation. (C) Van der Waals surface of bowl 1^6+^ (left) along with an energy-minimized structure of [MTX⊂1]^4+^ (OPLS3).

Methotrexate (MTX, Fig. 1A) is one of the world's essential drugs (World Health Organization) that the FDA has approved (in 1953) for treating neoplastic (including breast cancer, lung cancer, acute lymphocytic leukemia, osteosarcoma and non-Hodgkin's lymphoma) and autoimmune (rheumatoid arthritis and psoriasis) diseases.^1^ Importantly, 2–12% of cancer patients under a high-dose MTX regimen develop acute kidney injury (AKI) leading to myelosuppression, hepatotoxicity, neurotoxicity and eventually multiorgan failure.^2^ A standard countermeasure includes treatment with leucovorin,^3^ urine alkalinization and vigorous hydration to flush the molecule from the system. In more severe cases,^4^ or after chemotherapy medication errors,^5^ the enzyme glucarpidase (Voraxaze, approved by FDA in 2012), which is capable of rapidly hydrolysing MTX, is recommended.^6^ However, glucarpidase's distribution is limited to the cardiovascular system, so the enzyme is unable to reach cells across the intercellular matrix, requiring patients to additionally receive leucovorin. Furthermore, the enzyme does not cross the blood–brain barrier (BBB), necessitating its intrathecal injection (*i.e.*, spine) for treating toxic effects (such as encephalopathy, seizure and stroke) in leukemia patients.^7^ The high cost of glucarpidase is another deficiency, along with its inability to prevent fatal toxicity in 3% of the patients. In this work, we wondered if an alternative therapy for MTX overdose can be developed in the form of an inexpensive, abiotic cavitand capable of including MTX in its cavity for pharmacokinetic drug removal.^8^ This inspiration comes from sugammadex^9^ (*i.e.*, a derivative of γ-cyclodextrin), a cavitand that acts as a sequester of the neuromuscular relaxant rocuronium, thereby arresting the action of the relaxant and helping speed up the postoperative recovery of patients. In fact, Bridion (Merck) was approved by the FDA in 2015. Indeed, α-, β- and γ-cyclodextrin derivatives,^10^ cucurbit[7]uril^11^ and resorcinarenes^12^ bind to MTX by including either the pteridine or *p*-aminobenzoic moiety in their cylindrical cavity. However, the absence of host–guest complementarity, with only a partial desolvation of MTX, has resulted in the formation of complexes with only millimolar stability (*K*_d_ ∼ mM), so far.^13^ Other investigations, specifically X-ray studies of MTX in its free^14^ and enzyme-bound^15^ forms, revealed the drug folding its aromatic planes (*i.e.*, pteridine and *p*-aminobenzoyl ring; Fig. 1A) at a *circa* 90° angle. Moreover, MTX molecules assemble in the solid-state (Fig. 1A) such that a folded drug molecule is holding onto another *via* π–π stacking contacts and in an anti-parallel arrangement.^14*b*^ With this in mind, we wondered: can a tribenzotriquinacene (TBTQ) and bowl-shaped cavitand 1^6+^ (Fig. 1B and C), with *circa* 90° angle between its fused indanes,^16^ host MTX with the aromatics folded at ∼90° in aqueous media? First, the electron-rich surface^17^ of the TBTQ moiety of 1^6+^ (Fig. 1C) is expected to complement the electron-deficient pteridine from MTX. Second, six positively charged pyridinium units at the rim could ion-pair with the negatively charged glutamate of MTX while also enhancing the solubility of 1^6+^ in water. The energy-minimized structure of [MTX⊂1]^4+^ (Fig. 1C) suggested MTX^2−^ in its folded form occupying the cavitand with both pteridine and *p*-aminobenzoyl groups forming π–π stacking contacts and glutamate residing between the positively charged pyridinium moieties. While TBTQ cavitands have mostly been explored for complexing fullerenes in organic media,^18^ there are a handful of recent studies describing their inclusion complexation in water.^19^

**Fig. 2:**
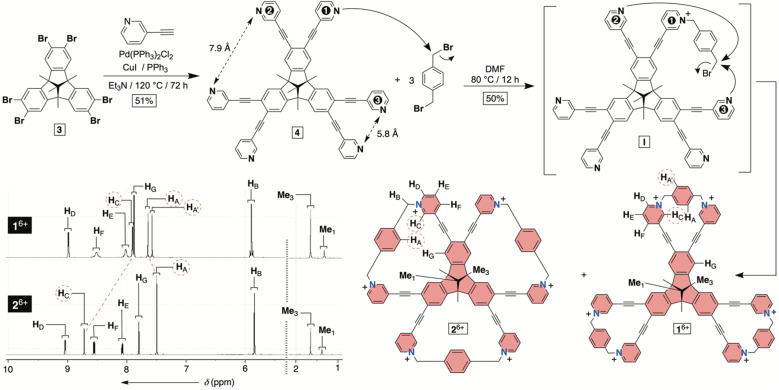
Synthesis of molecular bowls 1^6+^ and 2^6+^ with their ^1^H NMR spectra (850 MHz, 298 K) in water (30 mM phosphate buffer at pH = 7.4).

As for the main objectives of this study, we aimed to develop a facile synthetic method^20^ for accessing bowl-shaped 1^6+^ in addition to its constitutional isomer 2^6+^ (Fig. 2). Next, we set out to (a) examine conformational dynamics and assembly characteristics of molecular bowls, (b) quantify their affinity for capturing toxic anticancer drug MTX in aqueous media, (c) elucidate docking position(s) of MTX within each bowl and (d) quantify *in vitro* biocompatibility of these novel hosts.

## Results and discussion

### Syntheses of molecular bowls 1^6+^ and 2^6+^

**Fig. 3:**
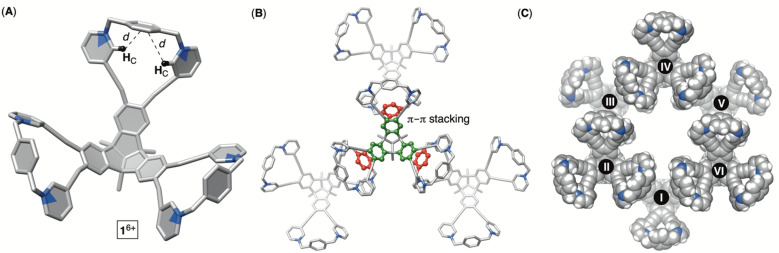
Different representations of the X-ray structure of molecular bowl 1^6+^ showing its conformation at the rim (A) and the modes of packing (B and C).

To obtain hexacationic bowl 1^6+^ (Fig. 2), we began with Sonogashira cross-coupling of 3-ethynylpyridine to hexabromo-tribenzotriquinacene 3.^21^ The process, requiring six consecutive covalent-bond formations, was effective and provided hexakis-pyridine 4 in 51% yield. The alkylation of 4 with an excess of 1,4-dibromoxylene was then conducted in *N*,*N*-dimethylformamide (DMF).^22^ Interestingly, an HPLC chromatogram of the reaction mixture (Fig. S1†) showed the presence of two main products in the ratio of *circa* 2 : 1. After isolation (35 and 15% yields), ^1^H NMR spectra of each product in water (Fig. 2) revealed a set of signals with the integration and resonance pattern corresponding to 4 alkylated with three 1,4-dibromoxylenes. Moreover, ^19^F NMR spectra (Fig. S15 and S23†) showed the presence of six trifluoroacetate anions for each *C*_3v_ symmetric molecule and ESI-MS corroborated their identical molecular weights (Fig. S13 and S21†). To rationalize the data, we reasoned that intermolecular alkylation of pyridine-1 with 1,4-dibromoxylene gives intermediate I (Fig. 2) which is set for intramolecular nucleophilic substitution in two distinct manners. In one case, pyridine-2 may act as a nucleophile to give the 18-membered pyridinium macrocycle on the way to the formation of 1^6+^. Meanwhile, proximal and nucleophilic pyridine-3 (Fig. 2) can compete to give the 23-membered pyridinium macrocycle on the way to the formation of 2^6+^. For energy-minimized 4 (AM1), the closest distance of two adjacent pyridine nitrogens is 5.8 and 7.9 Å (Fig. 2). With 6.6–7.0 Å separation of *syn*-periplanar pyridinium nitrogens in the solid state of related pyridinium macrocycles,^23^ we concluded that the formation of both 1^6+^ and 2^6+^ should have taken place (Fig. 2): energy-minimized 1^6+^ and 2^6+^ (OPLS3) have N-to-N gap of 6.6 and 7.2 Å, respectively. In the light of the above analysis, resonances from both ^1^H NMR spectra were fully assigned using results from ^1^H–^1^H NOESY, ^13^C–^1^H HMBC and ^13^C–^1^H HSQC spectra (Fig. S10–S12 and S18–S20†). Importantly, the more abundant product has two resonances H_A/A′_ corresponding to the benzene bridging pyridiniums at the rim. With hindered rotation of the benzenes, we assumed that the more shielded H_A′_ resides on the concave side of the cavitand. ^13^C NMR spectrum of the same product showed three lines from the same benzene ring (Fig. S9†). On the other hand, ^1^H NMR spectrum of the minor reaction product (Fig. 2B) showed a single resonance from benzene's H_A_ protons in addition to only one ^13^C NMR line from carbons carrying H_A_ nuclei (Fig. S17†). With hindered rotation of the benzene about its axis in the main product and free rotation in the minor one, we reasoned that the first molecule ought to be 1^6+^, including the smaller 18-membered pyridinium macrocycles, while the second isomer is 2^6+^ with the larger 23-membered pyridinium macrocycles (see also discussion below). The deduction also makes sense from the standpoint of presumably greater effective molarity^24^ for the annulation of smaller 18-membered rings within the major product 1^6+^ over the larger 23-membered rings in the minor product 2^6+^. Finally, X-ray diffraction analysis of crystals from the major product confirmed its correspondence to 1^6+^ (Fig. 3). Each pair of pyridiniums in 1^6+^ has its aromatic rings pointing in different directions (Fig. 3A) with a N-to-N distance of 6.6 Å. The three benzene rings that bridge the pyridinium moieties are horizontal with respect to the TBTQ, thereby residing on top of H_C_ (*d* = 2.5 and 3.5 Å; Fig. 3A). With ^1^H-NMR spectrum of 1^6+^ showing a magnetic shielding of H_C_ (Fig. 2), we reason that this conformational feature is retained in solution. Furthermore, within the crystal structure, each bowl is surrounded by three other bowls holding onto it *via* π–π stacking contacts (Fig. 3B). Electron-rich benzene rings from TBTQ are in this way stacked with electron-deficient and pyridinium-bridging benzenes (3.5 Å of centroid-to-centroid distance). Along the crystallographic *a*/*b* plane, bowls 1^6+^ organize into honeycomb-like (*i.e.*, hexagonal) prismatic cells^25^ (Fig. 3C) whereas their vertical stacking (*c* axis) into columns resembles the packing of coins in a stack.

### Conformational dynamics of bowls 1^6+^ and 2^6+^

**Fig. 4:**
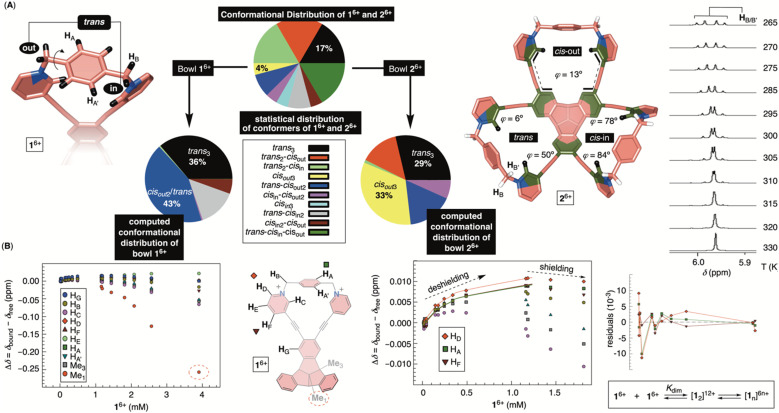
(A) Energy-minimized conformers of bowls 1^6+^ (left) and 2^6+^ (right, *trans*:*cis*_out_:*cis*_in_) with pie charts showing statistical and computed (DFT: B3LYP/6-31+G(d)) distributions of conformational stereoisomers. A segment of variable-temperature ^1^H NMR spectra of 2^6+^ in CD_3_OD showing a change in the shape of the signal from H_B_ as a function of temperature (265–330 K). (B) A change in the chemical shift of protons from 1^6+^ as a function of the bowl's concentration in water (30 mM PBS buffer at pH = 7.4). (Right) Dilution isotherms of H_D_, H_F_ and H_A_ protons from 1^6+^ fit well to a dimerization model (SigmaPlot) with a random distribution of residuals.

Molecular dynamics (MD) simulation of bowl 1^6+^ in a box of explicit water molecules revealed that the cavitand's TBTQ platform stays rigid while the pyridinium rim is conformationally flexible. For 200 ns of the MD simulation time, six methylene groups rocked back (*out*) and forth (*in*) thereby pulling pyridiniums to point away or toward the cavitand's inner space (Fig. 4A). For analysing the data, we identified three distinct positions of each pair of methylenes at the rim: *cis*_in_, *cis*_out_ and *trans* (Fig. 4A). With three juxtaposed arms, there are ten possible triplets of these co-orientations. Interestingly, MD simulations showed a similar statistical and computed distribution of stereoisomers. However, energy-minimization of conformers at a higher level of theory (DFT: B3LYP/6-31+G(d)) showed a distribution in which the *trans*_3_ stereoisomer dominates (Fig. 4A). To experimentally probe the methylene rocking, variable-temperature (VT) ^1^H NMR spectra of 1^6+^ revealed a singlet from diastereotopic H_B_ nuclei (Fig. 4A) in the interval of −5 to 55 °C (CD_3_OD, Fig. S14†). Supposedly, a conformational in/out motion of the methylene groups at the rim of 1^6+^ is occurring at a rapid rate to account for the observation (see also the discussion below). Additionally, the rotation of nearly horizontal bridging benzenes about their bonding axis was not taking place on the 200 ns time scale of the MD simulations. The result is supported with VT ^1^H NMR spectra of 1^6+^ (Fig. S14†) showing two singlets from benzene's H_A_ and H_A′_ over the entire −5 to 55 °C temperature range. To sum up, 1^6+^ is a molecular bowl with a rigid TBTQ bottom and flexible pyridinium moieties at the top.

As for the bowl 2^6+^, we built its ten distinct conformers using molecular mechanics. The energy minimization in implicit water solvent (DFT: B3LYP/6-31+G(d)) showed stereoisomers having a considerable free-energy difference (Fig. 4A). In particular, the Boltzmann-weighted population showed *trans*_3_ and *cis*_out3_ states dominating the distribution. We noted a trend for dihedral angles *φ* (Fig. 4A) characterizing 23-membered rings of 2^6+^. For *cis*_in_ and *cis*_out_ torsions, the dihedral angles *φ* are 80° and 13°, respectively, while the *trans* state appears to be a combination of the two with *φ*_1_ = 50° and *φ*_2_ = 6°. As *φ* denotes the degree of rotation of pyridiniums with respect to benzenes from TBTQ, it follows that by approaching *φ* = 0°, the extent of π-conjugation across the molecule increases. With smaller torsion angles increasing the stability of 2^6+^, the computed paucity of *cis*_in_ and the dominance of *cis*_out_/*trans* states makes sense. VT ^1^H NMR spectra of 2^6+^ in CD_3_OD (Fig. 4A; Fig. S22†) showed the resonance from methylene H_B_ protons as a singlet at higher and an AB quartet at lower temperatures. For each of the participating and exchanging conformational states (see pie chart in Fig. 4A), diastereotopic H_B_ protons shall give one or more AB quartets that at lower temperature would be expected to give multiple signals. Since only one AB quartet was observed, we sought for an alternative explanation. If methylene groups within 2^6+^ have similar magnetic characteristics, on both the inner or outer sides of the cavitand, then in/out movement of each CH_2_ will exchange the positions of its two diastereotopic CH_B_H_B′_ protons. At lower temperatures (*i.e.*, the slow exchange regime), this will give an AB quartet coalescing into a singlet as the rocking rate increases (*i.e.*, higher temperatures). The rate coefficient characterizing the process is, at the coalescence temperature of 315 K (Fig. 4A), estimated to 103 s^−1^
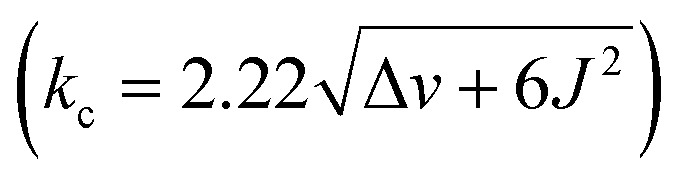
 thereby corresponding to Δ*G*^‡^ = 15.6 kcal mol^−1^.^26^ In addition, VT ^1^H NMR spectra of 2^6+^ (Fig. S22†) showed one singlet from benzene's H_A_ over the entire temperature range indicating a rapid rotation of benzene about its axis. To sum up, molecular bowl 2^6+^ has, like 1^6+^, a rigid TBTQ platform, albeit with a less dynamic and wobbling rim.

### Self-association of bowls 1^6+^ and 2^6+^

Molecular bowls 1^6+^ and 2^6+^ are amphiphilic compounds, each possessing a nonpolar bottom and a positively charged top. With the common observation of concave amphiphiles assembling in aqueous media,^27^ we decided to obtain ^1^H NMR spectra of variously concentrated 1^6+^ and 2^6+^ (30 mM PBS buffer at pH = 7.4; Fig. S24 and S26†). As shown in Fig. 4B, proton resonances from 1^6+^ showed a consistent change of their chemical shift as a function of cavitand's concentration indicating self-association. Interestingly, the proton nuclei are first getting deshielded to a small degree, with the chemical-shift change reaching a maximum point. After that, a greater degree of shielding follows with the central methyl (*i.e.*, Me_1_) at the bottom of 1^6+^ experiencing the largest perturbation. Presumably, the aggregation is characterized with two steps: nucleation in which an intermediate is accumulated (*i.e.*, NMR deshielding) followed by its oligomerization (*i.e.*, NMR shielding).^28^ NMR DOSY of variously concentrated 1^6+^ (Fig. S25†) revealed a small change in hydrodynamic radius of the cavitand during the nucleation phase while the apparent size increased in the oligomerisation. We deduced that the nucleation could be depicted as dimerization followed by the assembly of [1_2_]^12+^ into a more complex structure(s) (Fig. 4B). Indeed, the binding isotherm corresponding to nucleation fit well to a dimerization model^29^ with a random distribution of residuals and *K*_dim_ = 1396 ± 118 M^−1^ (Fig. 4B). Given the available data, it is difficult to elucidate the exact mode of the assembly, yet the observed magnetic deshielding of nuclei for dimer formation is in line with side-to-side stacking of the cavitands; in the solid-state, bowls 1^6+^ are indeed assembling side-to-side *via* π–π contacts (Fig. 3B). The magnetic shielding of the central methyl points to oligomerization characterized by the bowl-in-bowl type of complexation.^30^ On the other hand, ^1^H NMR chemical shifts of 2^6+^ as a function of concentration (Fig. S26†) resemble 1^6+^ with DOSY NMR (Fig. S27†), indicating formation of a dimer in the nucleation phase. Interestingly, bowl 2^6+^ forms a less stable dimer than 1^6+^ with *K*_a_ = 299 ± 161 M^−1^ (Fig. S26†). With the goal of the study centered on quantifying the inclusion complexation of MTX drug by monomeric bowls 1^6+^ and 2^6+^ (see below), we refrained from further examining the nature of hosts' extended aggregates.

### Inclusion complexation of methotrexate (MTX)

To probe the inclusion complexation of methotrexate (MTX), we began with the notion that both bowls self-associate in water. Regarding such equilibria, the results from above measurements suggested that the extent of dimerization is small (<6%) when [1]^6+^ or [2]^6+^ is less than 90 μM. Based on that but also the observed trend of chemical shifts describing the aggregation of 1^6+^ or 2^6+^ (Fig. 4C), we assumed that self-association equilibria can be neglected at μM concentrations. From the standpoint of MTX^2−^, the results of ^1^H NMR dilution measurements (Fig. S28†) were in line^31^ with the formation of its dimer in water.^32^ With *K*_a_ = 12 M^−1^, it necessitates a high (*i.e.*, >1 M) concentration of MTX^2−^ to prompt any appreciable self-association. Hence, we reasoned that ^1^H NMR spectroscopic titration^33^ of a standard solution of MTX^2−^ to <90 μM solution of 1^6+^ or 2^6+^ shall give results for which only equilibria depicting host–guest interaction(s) would play the major role.

**Fig. 5:**
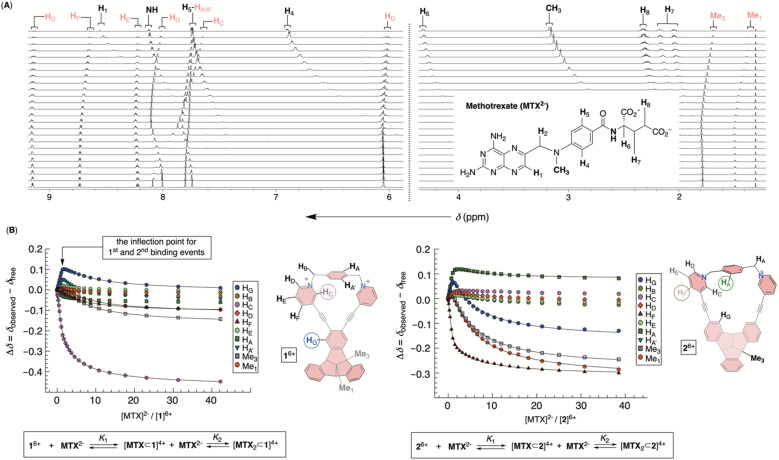
(A) Segment of ^1^H NMR spectra of 47 μM 1^6+^ in water (30 mM PBS buffer at pH = 7.4) obtained after incremental addition of 2.5 mM solution of MTX^2−^. (B) A relative change of the chemical shift of protons from 1^6+^ (left) or 2^6+^ (right) was fit to 1 : 2 model describing the formation of binary [MTX⊂1]^4+^ and ternary [MTX_2_⊂1]^2+^ complexes.

**Fig. 6:**
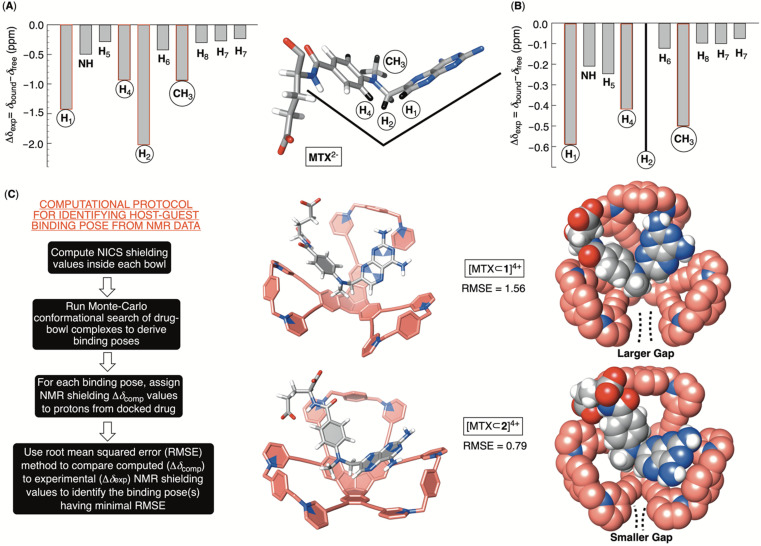
Relative ^1^H NMR spectroscopic perturbation of resonances Δ*δ*_exp_ from methotrexate during supramolecular titrations with bowls 1^6+^ (A) or 2^6+^ (B); chemical shift of H_2_ could not be obtained for titration with 2^6+^. (C) The computational protocol for finding the docking pose of MTX^2−^ inside bowls 1^6+^ and 2^6+^ along with binary [MTX⊂1]^4+^ and [MTX⊂2]^4+^ complexes (RMSE 1.56 and 0.79) obtained from the procedure.

Incremental addition of a standard solution of MTX^2−^ to 1^6+^ caused a steady perturbation of ^1^H NMR resonances to both host and guest (Fig. 5). Interestingly, most resonances from 1^6+^ underwent a magnetic deshielding first only to be shielded at higher proportions of the drug (Fig. 5A and S29†). With noncovalent bowl-to-drug association comprising two or more steps, we found that the binding isotherm fit well to 1 : 2 model of complexation (Fig. 5B and S30†). The formation of 2 : 1 ternary complex comprising two bowls and one drug would fit the data although with a greater covariance and less satisfactory residuals.^34^ Indeed, ESI mass spectrometry of a mixture of 1^6+^ and MTX^2−^ revealed signals corresponding to ternary [MTX_2_⊂1]^2+^ complex (Fig. S31†), in line with binding isotherms from ^1^H NMR titration. The stabilities of binary [MTX⊂1]^4+^ and ternary [MTX_2_⊂1]^2+^ complexes were, from four independent measurements in the millimolar range, *K*_1_ = 4.4 ± 3.1 × 10^4^ M^−1^ and *K*_2_ = 1.9 ± 0.3 × 10^3^ M^−1^. For the first binding event, the resonance from H_C_ (pink in Fig. 5B) and H_G_ (blue in Fig. 5B) nuclei, lining sides and bottom of the cavitand's inner space, experienced the largest magnetic perturbation. At the same time, all resonances from the drug within binary [MTX⊂1]^4+^ were magnetically shielded (Fig. 5A; see also Fig. 6A). As originally anticipated, we conclude that [MTX⊂1]^4+^ is an inclusion complex^35^ in which methotrexate occupies the cavity of 1^6+^, thereby residing in the diamagnetic shielding region of the host. With the inner space occupied, binary [MTX⊂1]^4+^ is likely to associate with another MTX^2−^ by holding it its outer and nonpolar surface. Perhaps, a shielding of the host's Me_1_ and Me_3_ resonances by the guest in the second binding event (Fig. 5B) provides evidence to such hypothesis. An incremental addition of MTX^2−^ to molecular bowl 2^6+^ resulted in perturbation of ^1^H NMR resonances from both host and guest (Fig. 5B and S32†). Interestingly, the first binding event was, in this case, characterized by H_F_ (brown in Fig. 5C) and H_A_ (green in Fig. 5C) nuclei experiencing the largest magnetic perturbation, thereby suggesting a somewhat different binding mode from the previous case. Again, ESI mass spectrometry of a mixture of 2^6+^ and MTX^2−^ provided evidence to the formation of ternary [MTX_2_⊂2]^2+^ (Fig. S34†) and the binding isotherm fit well to 1 : 2 model of complexation with *K*_1_ = 2.17 ± 0.11 × 10^5^ M^−1^ and *K*_2_ = 1.88 ± 0.01 × 10^3^ M^−1^ (Fig. 5B and S33†). Importantly, the stability of binary [MTX⊂2]^4+^ is an order of magnitude greater than [MTX⊂1]^4+^, thereby approaching μM range (*i.e.*, *K*_d_ = 4.6 μM for [MTX⊂2]^4+^) which does correspond to micromolar concentration of MTX in the blood of actual patients.^4^ Moreover, the stabilities of ternary [MTX_2_⊂1]^2+^ and [MTX_2_⊂2]^2+^ are practically the same and consistent with sterically less demanding, face-to-face, noncovalent contacts of binary complexes and methotrexate.

### Computational and experimental protocols for elucidating docking pose of MTX^2−^ within 1^6+^ and 2^6+^

The inclusion of MTX^2−^ inside bowls 1^6+^ and 2^6+^ resulted in the greatest degree of diamagnetic shielding of H_1_, H_4_, H_2_ and CH_3_ from methotrexate (Fig. 6A, B, S29 and S32†). As originally anticipated, the data suggest the drug occupies each cavitand in its folded form (Fig. 6A), thereby allowing H_1_, H_4_, H_2_ and CH_3_ can reach deep inside the aromatic binding pocked. Towards the elucidation of the drug's binding pose for [MTX⊂1]^4+^ and [MTX⊂2]^4+^, we decided to compute nucleus independent chemical shifts (NICS)^36^ inside each bowl (in the most stable *trans*_3_ conformation) and thus map the magnetic environment of their inner space.^37^ Next, we aimed to run Monte Carlo conformational searches for both [MTX⊂1]^4+^ and [MTX⊂2]^4+^ using a force field suitable for organic molecules (OPLS3). By allowing the conformational change of docked MTX^2−^ while freezing the motion of bowls, we hoped to generate a variety of binding poses between guest and host. Finally, we would assign the computed shielding value for each proton of MTX^2−^ docked inside 1^6+^ or 2^6+^ (Δ*δ*_comp_) by using the calculated NICS map for each host, and then we compared the computed values against the experimental ones (Δ*δ*_exp_; Fig. 6A and B). The pose with the lowest root mean squared error, 
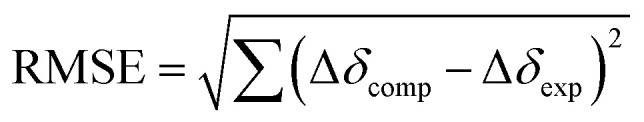
, is assumed to provide the best match with the experiment. After computing the magnetic environment of 1^6+^ and 2^6+^ and running the conformational search of each bowl holding a drug in its cavity, we subjected the data to RMSE analysis. Fascinatingly, the lowest RMSE value was found to correspond to conformers of [MTX⊂1]^4+^ and [MTX⊂2]^4+^ (Fig. 6C) having a folded MTX^2−^ facing the concave surface of the bowl to form π–π and C–H–π contacts (Fig. 6C). The glutamate moiety of MTX^2−^ sits between the cavitand's arms to create ion-pair contacts with pyridinium groups. As MTX^2−^ binds to 1^6+^ or 2^6+^ in the similar manner, how do we account for the large stability difference of their binary complexes? By inspecting CPK representations of [MTX⊂1]^4+^ and [MTX⊂2]^4+^ in Fig. 6C, one can easily note that bowl 1^6+^ has larger gaps between its macrocyclic arms than 2^6+^. In other words, bowl 2^6+^ is more effectively encircling the space with its concave surface capable of, we posit, more effectively desolvating the MTX guest. Indeed, the folded MTX^2−^ is inserting into [MTX⊂2]^4+^ such that the pteridine moiety faces the macrocyclic arm while the *p*-aminobenzoic group covers the gap. In the case of [MTX⊂1]^4+^ though, both pteridine and *p*-aminobenzoic group face the gaps between macrocyclic arms of this host. A greater degree of desolvation (*i.e.*, hydrophobic effect) is therefore suggested to contribute to the greater stability of [MTX⊂2]^4+^ over [MTX⊂1]^4+^.

### Cytotoxicity of molecular bowls 1^6+^ and 2^6+^

**Fig. 7:**
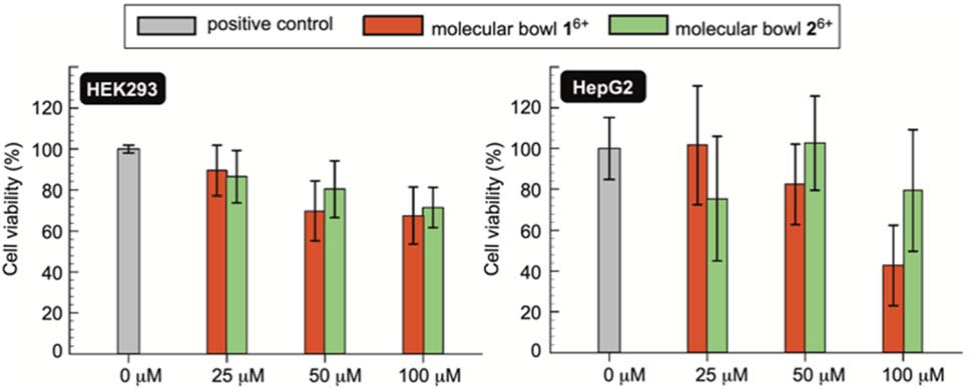
The cell viability (%) of HEK293 (left) and HepG2 (right) cells were quantified using MTS assays without (positive control, gray bars) and with molecular bowls 1^6+^ (red bars) and 2^6+^ (green bars) at their respective 25, 50 and 100 μM concentrations. Each measurement was completed three times (48 h incubation time) with values reported as median ± standard deviation.

If novel hosts 1^6+^ and 2^6+^, or their future variants, are to act as sequesters of toxic methotrexate (MTX) in blood circulation,^4^ they need to be innocuous to human cells.^38^ To examine *in vitro* biocompatibility of 1^6+^ and 2^6+^, we completed MTS assays^39^ using human kidney (HEK 293) and human liver cancer (HepG2) cell lines. MTS assay is a quantitative colorimetric method in which NAD(P)H-dependent dehydrogenase enzymes from healthy cells reduce 3-(4,5-dimethylthiazol-2-yl)-5-(3-carboxymethoxyphenyl)-2-(4-sulfophenyl)-2*H*-tetrazolium (MTS) into colored and water-soluble formazan dye.^40^ By measuring UV-Vis absorbance of the formazan produced by metabolically active cells, cellular viability can be quantified.^41^ Importantly, as methotrexate is known to cause renal^2*b*^ (kidney) and hepatotoxicity^42^ (liver), we decided to probe the effect of molecular bowls 1^6+^ and 2^6+^ on the viability of HEK293 and HepG2 cell lines. In this regard, a positive control was established by incubating wells of untreated cell media in both the HEK293 and HepG2 lines (Fig. 7). When HEK293 cells were incubated in the presence of 25–100 μM of bowls 1^6+^ and 2^6+^, the viability dropped to *circa* 80% to be in line with both hosts having negligible toxic effect on the kidney cell line. On the other side, bowl 1^6+^ revealed a somewhat greater cytotoxicity toward HepG2 with the viability dropping as a function of its concentration (Fig. 7). Bowl 2^6+^ showed acceptable biocompatibility with the viability fluctuating around 80% (Fig. 7); note that this bowl is the more effective sequester of toxic MTX. With IC_50_ (half maximal inhibitory concentration)^25^ of 1^6+^ and 2^6+^ greater than 100 μM (Fig. 7) we conclude that molecular bowls are biocompatible hosts and promising candidates for further examining the sequestration of toxic MTX, for which IC_50_ = 78 nM.^43^

## Conclusions

In conclusion, molecular bowls 1^6+^ and 2^6+^ are novel, modular, and accessible hosts. Their curved nonpolar platform with a positively charged and macrocyclic rim makes amphiphilic 1^6+^ and 2^6+^ undergo self-association in water. Moreover, molecular bowls are relatively rigid and preorganized molecules capable of including aromatic methotrexate (MTX): the curved platform of 1^6+^/2^6+^ has three fused indane rings, arranged at 90° angles, that are complementary to anticancer MTX having two aromatic moieties folded at *circa* 90°. Both experimental and computational studies suggested that the folded drug occupies the cavity of bowls wherein it forms π–π, C–H–π, and ion pairing intermolecular contacts but also undergoes desolvation to give stable binary complexes. In this regard, we described a computational protocol for identifying docking pose(s) of MTX inside molecular bowls that may be applied toward elucidation of binding of other host–guest systems in supramolecular chemistry. Finally, bowl 2^6+^ is, to our knowledge, the strongest binder of MTX reported to date, thus holding promise as a potential sequestering agent capable of removing the drug from overdosed cancer patients. In this vein, both 1^6+^ and 2^6+^ showed minimal adverse effects on the growth of human kidney (HEK 293) and liver cancer (HepG2) cells thereby attesting to the biocompatibility of this class of hosts.

Our next objectives center on tuning the structure of molecular bowls to minimize their self-association but also to permit selective recognition of MTX in the presence of similarly sized and shaped leucovorin and folic acid. This will set the stage for probing the action of this intriguing family of hosts in biological systems.

## Data availability

The data that support our findings are available on request from the corresponding author.

## Author contributions

Pratik Karmakar synthesized hosts, studied their aggregation and binding to MTX. Tyler J. Finnegan completed computational studies. Darian C. Rostam assisted with synthesis of hosts and study of their biocompatibility. Sagarika Taneja completed biocompatibility studies. Sefa Uçar worked on synthesis of hosts. Alexandar L. Hansen assisted with NMR studies. Curtis E. Moore collected X-ray data and processed them. Christopher M. Hadad assisted with interpretation of computational data. Kornkanya Pratumyot worked on the synthesis of hosts and interpretation of data. Jon R. Parquette directed biocompatibility studies and interpretation of data. Jovica D. Badjić wrote the paper, directed all experimental and computational studies and interpretation of data.

## Conflicts of interest

There are no conflicts to declare.

## Supplementary Material

SC-015-D3SC05627A-s001

SC-015-D3SC05627A-s002
