# Supplementary material for: Molecular bowls for inclusion complexation of toxic anticancer drug methotrexate
Source: Chem Sci. 2024 May 28;15(26):10155–63. doi: 10.1039/d3sc05627a (PMC11220613; doi:10.1039/d3sc05627a)
Supplement: SC-015-D3SC05627A-s001 [file SC-015-D3SC05627A-s001.pdf]

Electronic Supplementary Material (ESI) for Chemical Science.
This journal is © The Royal Society of Chemistry 2024

Molecular Bowls for Inclusion Complexation of Toxic Anticancer Drug Methotrexate

Pratik Karmakar,[a][b] Tyler J. Finnegan,[a] Darian C. Rostam,[a] Sagarika Taneja,[a] Sefa Uçar, [a][c] Alexandar L. Hansen,[d] Curtis E. Moore,[a] Christopher M. Hadad,[a] Kornkanya Pratumyot,[b] Jon R. Parquette[a] and Jovica D. Badjić*[a]

[a] Dr. P. Karmakar, T. J. Finnegan, D. C. Rostam, S. Taneja, Dr. C. E. Moore, Prof. C. M. Hadad , Prof. J. R. Parquette and Prof. J. D. Badjić
Department of Chemistry and Biochemistry
The Ohio State University
100 West 18th Avenue, Columbus, Ohio 43210 (USA)
E-mail: badjic.1@osu.edu

[b] Dr. P. Karmakar and Dr. K. Pratumyot
Supramolecular Chemistry Research Unit, Department of Chemistry, Faculty of Science
King Mongkut's University of Technology Thonburi
126 Pracha Uthit Road, Bang Mod, Thung Khru, Bangkok 10140 (Thailand)

[c] Dr. Sefa Uçar
Atatürk University,
Faculty of Science, Department of Chemistry,
Erzurum 25240, Turkey

[d] Dr. A. L. Hansen
Campus Chemical Instrumentation Center
The Ohio State University
100 West 18th Avenue, Columbus, Ohio 43210 (USA)

Supporting Information

Table of Contents

1. General Information……………………………………………………S3

2. Synthetic Procedures………………………………………………….…...S4

3. Spectroscopic Characterizations…………………………….…...…...S7

4. Host-Guest Binding Studies….……………………………………..S19

5. Computational Studies…………………………………………………S31

6. X-Ray Studies…………………………………………………………….S80

7. Cell Studies…………………………………………………...….S98

General Information

All chemicals were purchased from commercial sources and used as received unless stated otherwise. All solvents were dried prior to use according to standard literature procedures. Chromatographic purifications were performed with silica gel 60 (SiO_2, Sorbent Technologies 40-75 μm, 200 x 400 mesh). Thin-layer chromatography (TLC) was performed on silica-gel plate w/UV254 (200 μm). For NMR studies, we used class B glass NMR tubes (Wilmad Lab Glass). NMR experiments were performed with Bruker 400, 600, 700, 800 and 850 MHz spectrometers. Chemical shifts are expressed in parts per million (δ, ppm) while coupling constant values (J) are given in Hertz (Hz). Residual solvent resonances were used as internal standards: for ^{1}H NMR spectra CD_2Cl_2 = 5.32 ppm, CD_3OD = 3.31 ppm and D_2O = 4.79 ppm while for ^{13}C NMR spectra CD_2Cl_2 = 53.84 ppm, CD_3OD = 49.00 ppm. Deuterated solvents CD_2Cl_2, $CDCl_3$, CD_3OD and D_2O were purchased from Cambridge Isotope Laboratories. HRMS data were obtained on a Bruker Impact II QqTOF instrument in the positive mode.

Synthetic Procedures

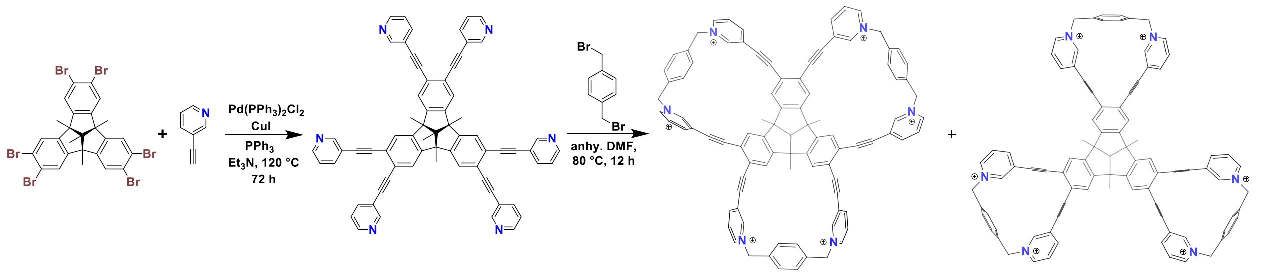

3 4 (51%) 2^{6+} (15%) 1^{6+} (35%)

Scheme S1. Synthetic scheme describing the preparation of bowls $\mathbf{1}^{6+}$ and $\mathbf{2}^{6+}$; after HPLC purification, the bowls are in the form of trifluoroacetate salts. Hexabromo-tribenzotriquinacene **3** was obtained by following the existing procedure (see: Kuck, D., Schuster, A., Krause, R. A., Tellenbroker, J., Exner, C. P., Penk, M., Bogge, H., Muller, A., *Tetrahedron* **2001**, *57*, 3587-3613).

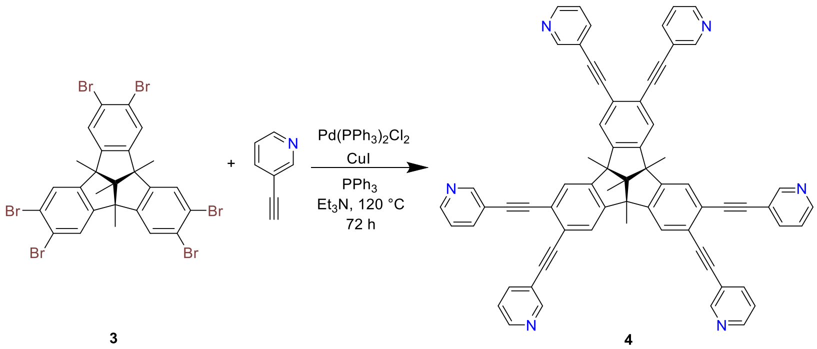

3 4

Compound 4: Hexabromo-tribenzotriquinacene **3** (200 mg, 247.0 μmol), 3-alkynyl pyridine (611.22 mg, 5.93 mmol), $Pd(PPh_3)_2Cl_2$ (52.0 mg, 74.1 μmol), triphenylphosphine (38.87 mg, 148.2 μmol) and CuI (28.22 mg, 148.2 μmol) were mixed together in a glass vial inside a glove box. The vial was then closed using septum and taken outside of the glovebox. Degassed triethylamine (10.0 ml) was added, and the mixture was stirred at 120 °C for 72 h. The resulting mixture was filtered through a plug of cotton, evaporated, dissolved in 100 ml of dichloromethane, and then extracted with saturated aqueous solution of NH_4Cl, water and brine. The organic layer was dried using sodium sulfate and evaporated to give crude product. After purification with column chromatography (SiO_2; from CH_2Cl_2 to CH_3OH:CH_2Cl_2 = 1:18 and CH_3OH:CH_2Cl_2 = 1:12), 120 mg of **4** (51 %) as a white crystalline solid was obtained; if necessary, the solid can be triturated with acetone to improve its purity. ^{1}H NMR (600 MHz, 20:1 CD_2Cl_2/CD_3OD) δ 8.76 (bs, 6H), 8.53 (bs, 6H), 7.91 (d, *J* = 7.5 Hz, 6H), 7.71 (s, 6H), 7.38 (bs, 6H), 1.74 (s, 9H), 1.42 (s, 3H). ^{13}C NMR (176 MHz, 20:1 CD_2Cl_2/CD_3OD) δ 151.78, 149.66, 148.64, 139.55, 127.36, 125.40, 124.12, 120.97, 91.81, 90.12, 71.26, 63.29, 25.70, 16.11. HRMS (ESI): *m*/*z* calcd for $C_{68}H_{43}N_6^+$ $[M+H]^+$: 943.3544; found: 943.3552.

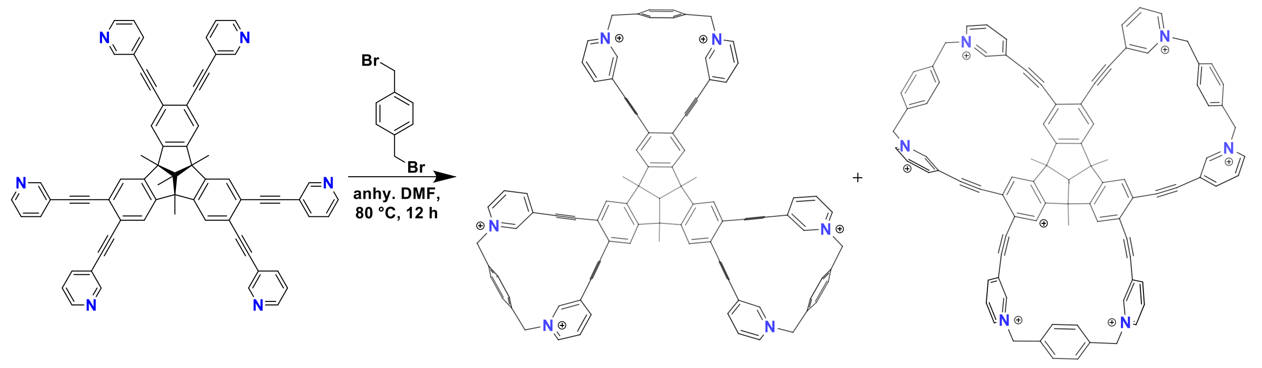

4 **1^{6+} (Major Product)** **2^{6+} (Minor Product)**

Molecular Bowls 1^{6+} and **2^{6+}**: Under an atmosphere of nitrogen, compound **4** (100 mg, 106.0 µmol) and 1,4-dibromoxylene (93 mg, 350 µmol) were stirred in 10 ml of anhydrous DMF at 80 °C for 12 h. The solvent was evaporated and the remaining solid was dissolved in 25 ml of water. The suspension was centrifuged, and the supernatant subjected to purification by HPLC chromatography. For this, 2.0 ml of the supernatant solution was, at a time, injected into an HPLC equipped with a reverse phase column (preparative Waters XBridge BEH C8) and then eluted with a linear gradient of CH_3CN/H_2O (20/100 to 100/0 over 60 minutes) containing 0.1% TFA. The collected fractions were then lyophilized to give **$1(CF_3CO_2)_6$** as a white solid (47.5 mg, 35%) and **$2(CF_3CO_2)_6$** as a yellow solid (20.0 mg, 15%).

Compound $1(CF_3CO_2)_6$: ^{1}H NMR (700 MHz, D_2O) δ 9.05 (d, *J* = 6.1 Hz, 6H), 8.73 (bs, 6H), 8.56 (d, *J* = 8.2 Hz, 6H), 8.08 (dd, *J* = 8.1, 6.3 Hz, 6H), 7.80 (s, 6H), 7.50 (s, 12H), 5.87 – 5.81 (AB quartet, 12H, J = 14.98 Hz), 1.65 (s, 9H), 1.38 (s, 3H). ^{13}C NMR (176 MHz, D_2O) δ 150.43, 147.75, 144.30, 144.11, 132.89, 132.81, 132.74, 128.92, 127.69, 124.24, 122.91, 94.34, 85.76, 70.61, 63.90, 62.87, 24.19, 14.75. HRMS (ESI): m/z calcd for $C_{92}H_{66}N_6^{4+}$ $[M]^{4+}$: 313.6332; found: 313.6329.

Compound $2(CF_3CO_2)_6$: ^{1}H NMR (700 MHz, D_2O) δ 9.08 (d, *J* = 6.2 Hz, 6H), 8.61 (bs, 6H), 8.12 (bs, 6H), 8.00 (s, 6H), 7.97 (s, 6H), 7.74 (s, 6H), 7.67 (s, 6H), 6.01 – 5.96 (AB quartet, 12H, J = 14.56 Hz), 1.74 (s, 9H), 1.42 (s, 3H). ^{13}C NMR (176 MHz, D_2O) δ 150.38, 147.51, 145.65, 144.60, 133.90, 130.47, 128.44, 128.17, 124.68, 122.99, 94.65, 85.61, 70.03, 64.25, 62.94, 23.78, 13.19. HRMS (ESI): m/z calcd for $C_{92}H_{66}N_6^{4+}$ $[M]^{4+}$: 313.6332; found: 313.6320.

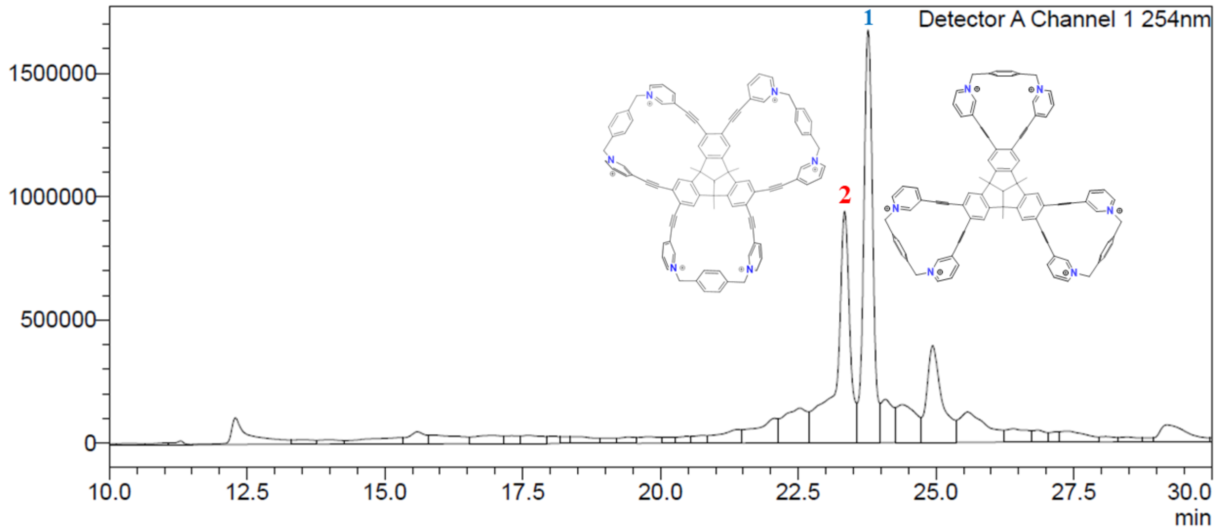

Figure S1. An HPLC chromatogram (reverse-phase) from a crude reaction mixture showing two main peaks corresponding to bowls **1**$^{6+}$ and **2**$^{6+}$; for the experiment, we used preparative Waters XBridge BEH C8 column eluted with a linear gradient of CH_3CN/H_2O containing 0.1% TFA (20/100 to 100/0 over 60 minutes).

Spectroscopic Characterizations

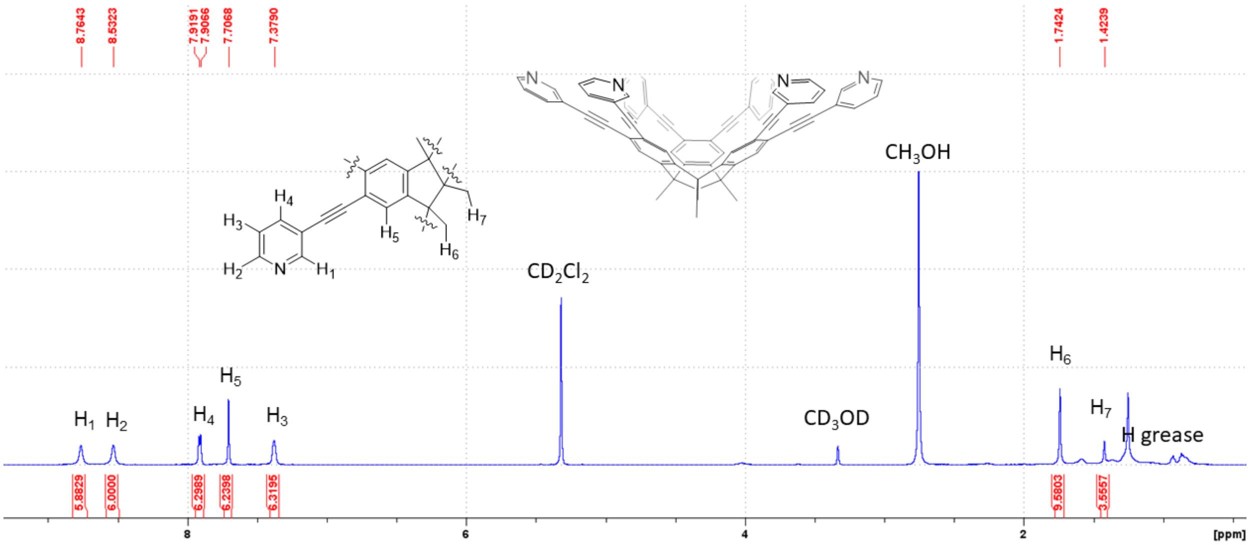

Figure S2. ^{1}H NMR spectrum (600 MHz, 20:1 CD_2Cl_2/CD_3OD, 298K) of Compound **4**.

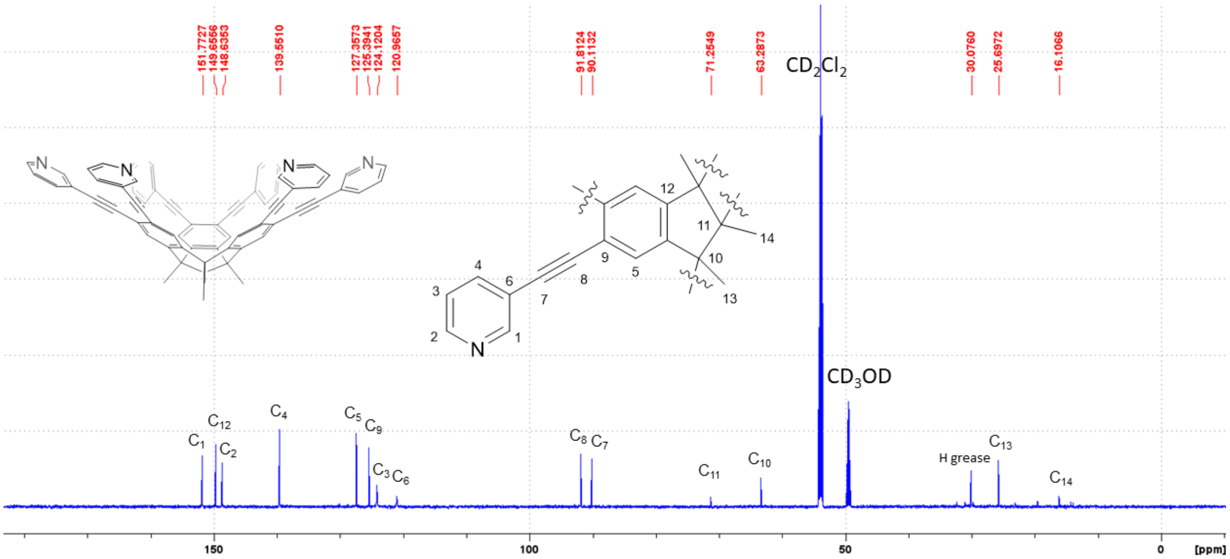

Figure S3. ^{13}C NMR spectrum (176 MHz, 20:1 CD_2Cl_2/CD_3OD, 298K) of compound **4**.

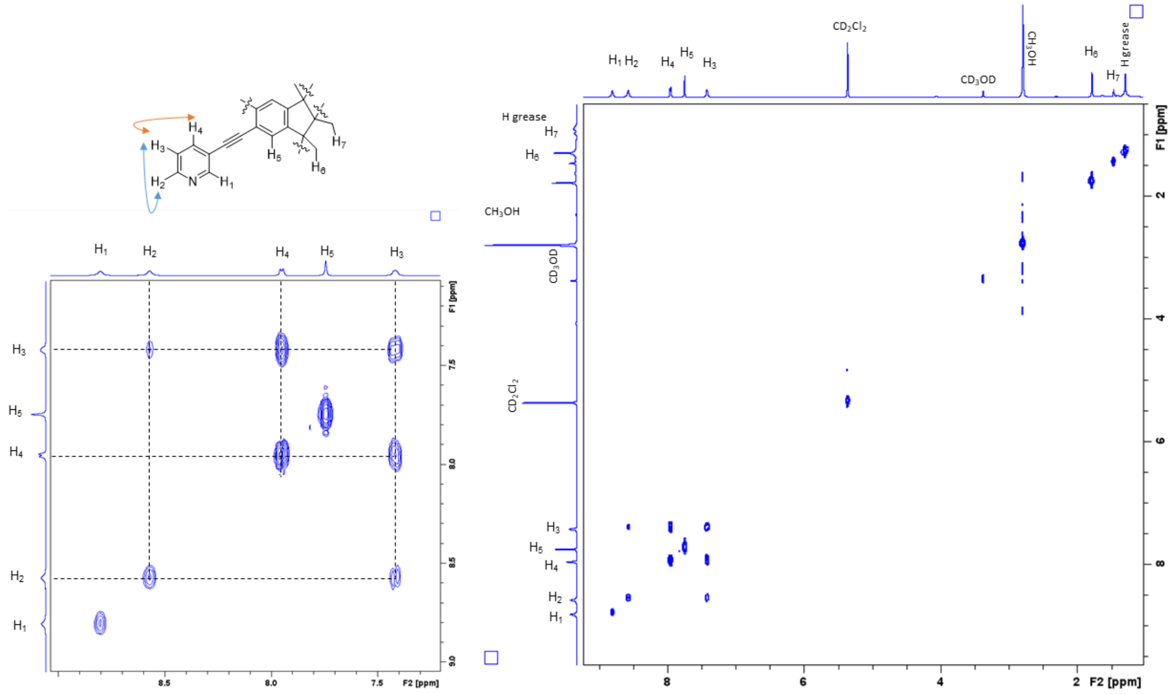

Figure S4. Two views of ^{1}H-^{1}H COSY NMR spectrum (600 MHz, 20:1 CD_2Cl_2/CD_3OD, 298K) of compound **4**.

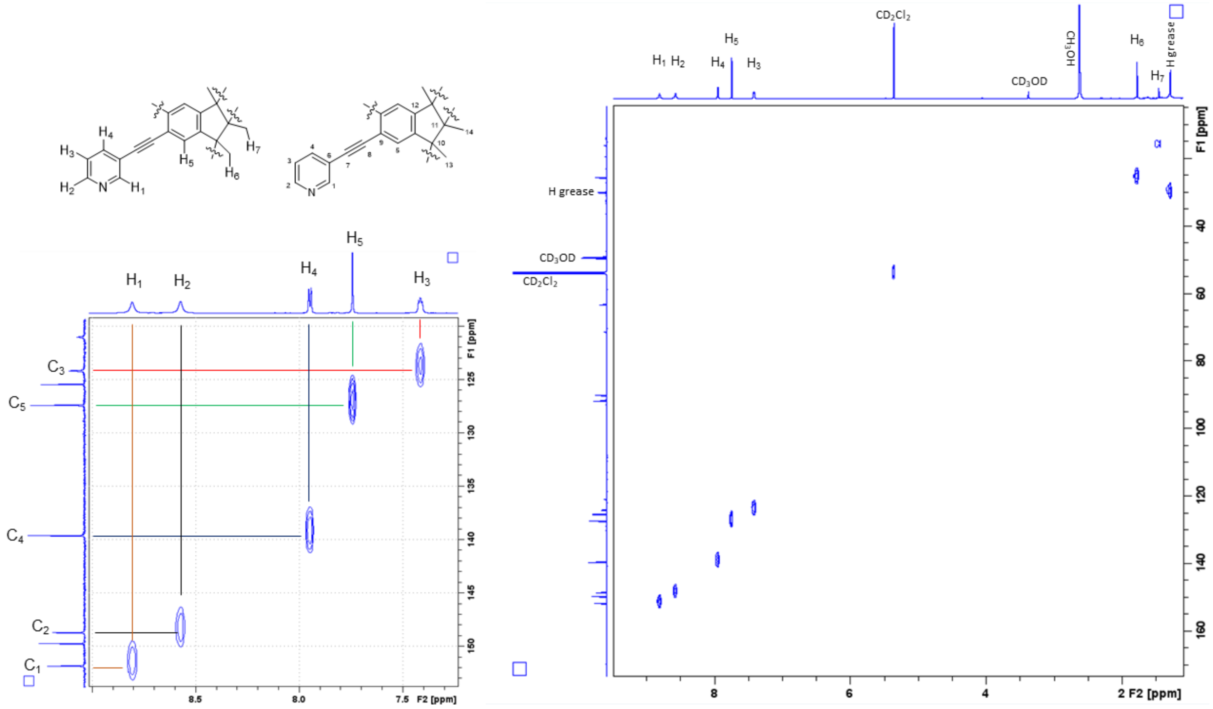

Figure S5. Two views of ^{1}H-^{13}C HSQC NMR spectrum (700 MHz, 20:1 CD_2Cl_2/CD_3OD, 298K) of compound **4**.

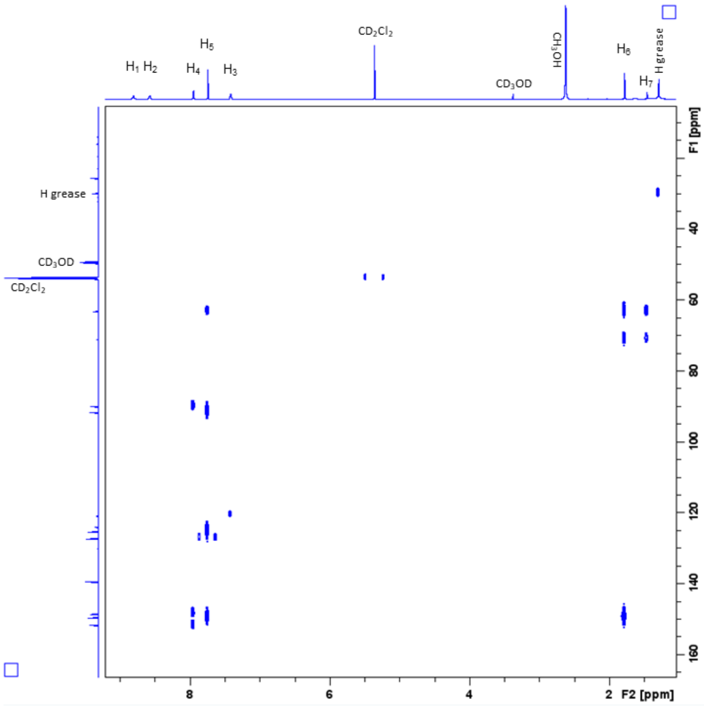

Figure S6. ^{1}H-^{13}C HMBC spectrum (700 MHz, 20:1 CD_2Cl_2/CD_3OD, 298K) of compound **4**.

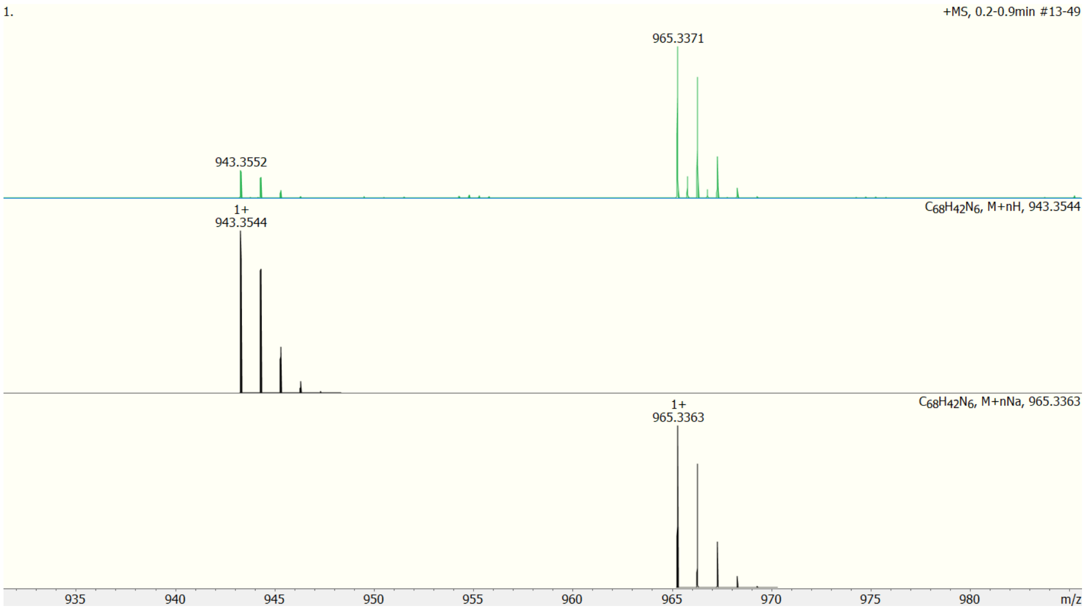

Figure S7. Segments of experimental (top) and simulated (bottom two) high-resolution ESI-MS spectra of **4** using positive mode with simulated isotopic pattern, suggesting the formation of [**4**+H]$^{+}$ cation.

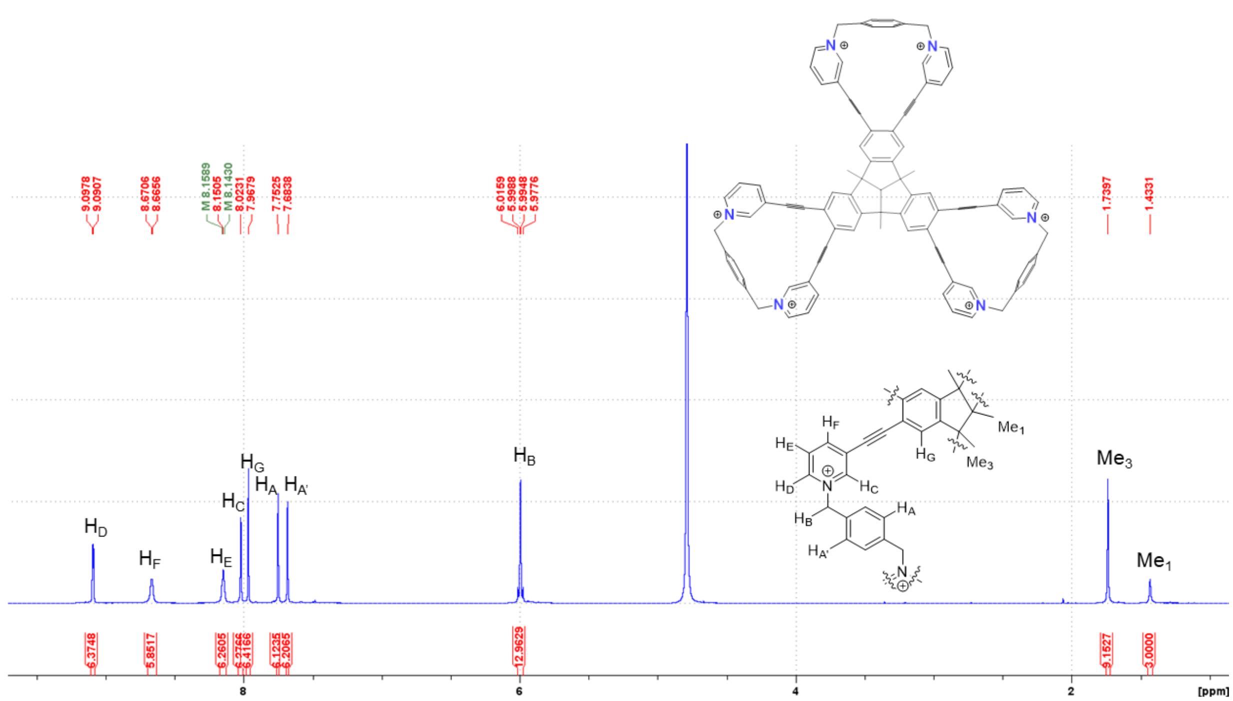

Figure S8. ^{1}H NMR spectrum (600 MHz, D_2O, 298K) of bowl **1**$^{6+}$.

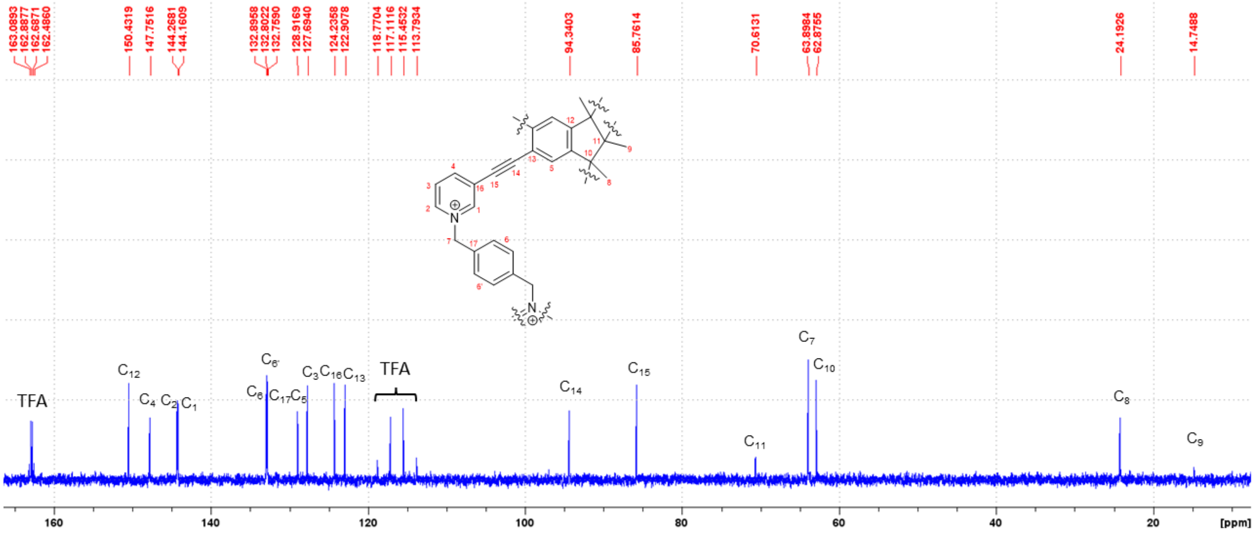

Figure S9. ^{13}C NMR spectrum (176 MHz, D_2O, 298K) of bowl **1**$^{6+}$.

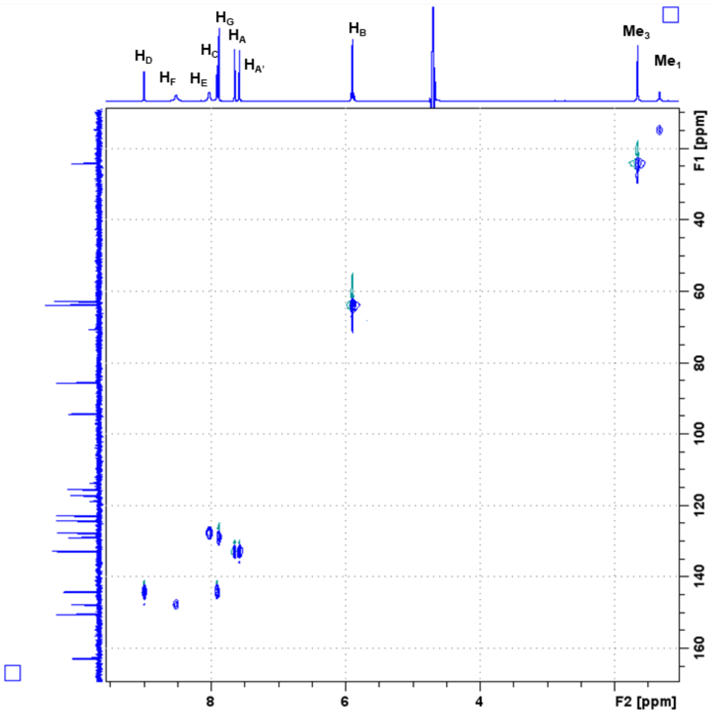

Figure S10. ^{1}H-^{13}C HSQC spectrum (700 MHz, D_2O, 298K) of bowl **1**$^{6+}$.

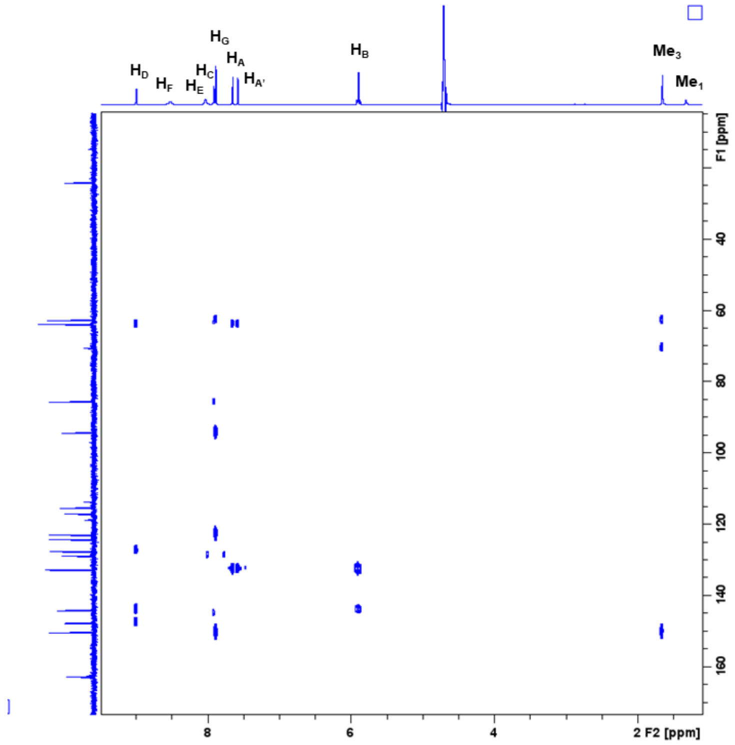

Figure S11. ^{1}H-^{13}C HMBC spectrum (700 MHz, D_2O, 298K) of bowl **1**$^{6+}$.

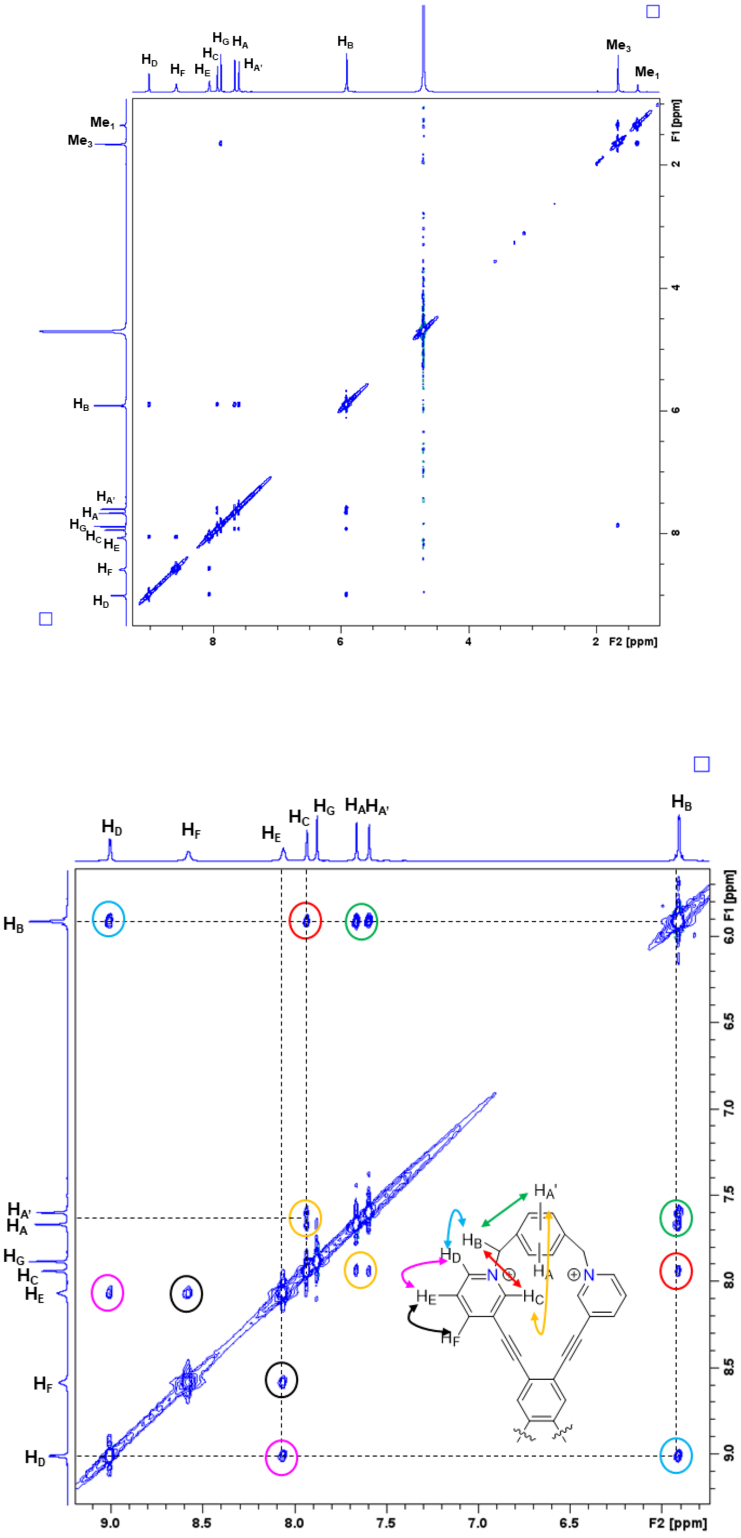

Figure S12. Two views of ^{1}H-^{1}H NOESY spectrum (850 MHz, D_2O, 298K) of bowl **1**$^{6+}$, with the assigned cross peaks.

a.

b.

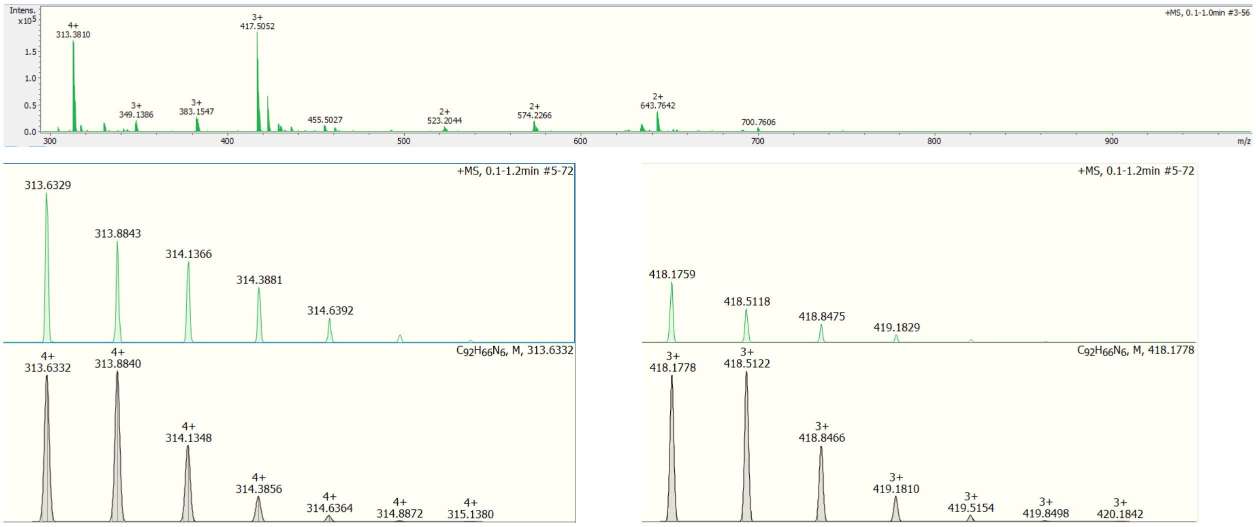

Figure S13. (a) Positive mode HRMS-ESI of bowl **1**$^{6+}$ in ammonium bicarbonate buffer (green). (b) Segments of experimental (top) and simulated (bottom) HRMS-ESI of **1**$^{6+}$. The formation of [**1**]$^{4+}$ cation with *m/z* calcd for $C_{92}H_{66}N_6^{4+}$ 313.6332 (bottom), found 313.6332 (top) and [**1**]$^{3+}$ cation with *m/z* calcd for $C_{92}H_{66}N_6^{3+}$ 418.1778 (bottom), found 418.1759 (top).

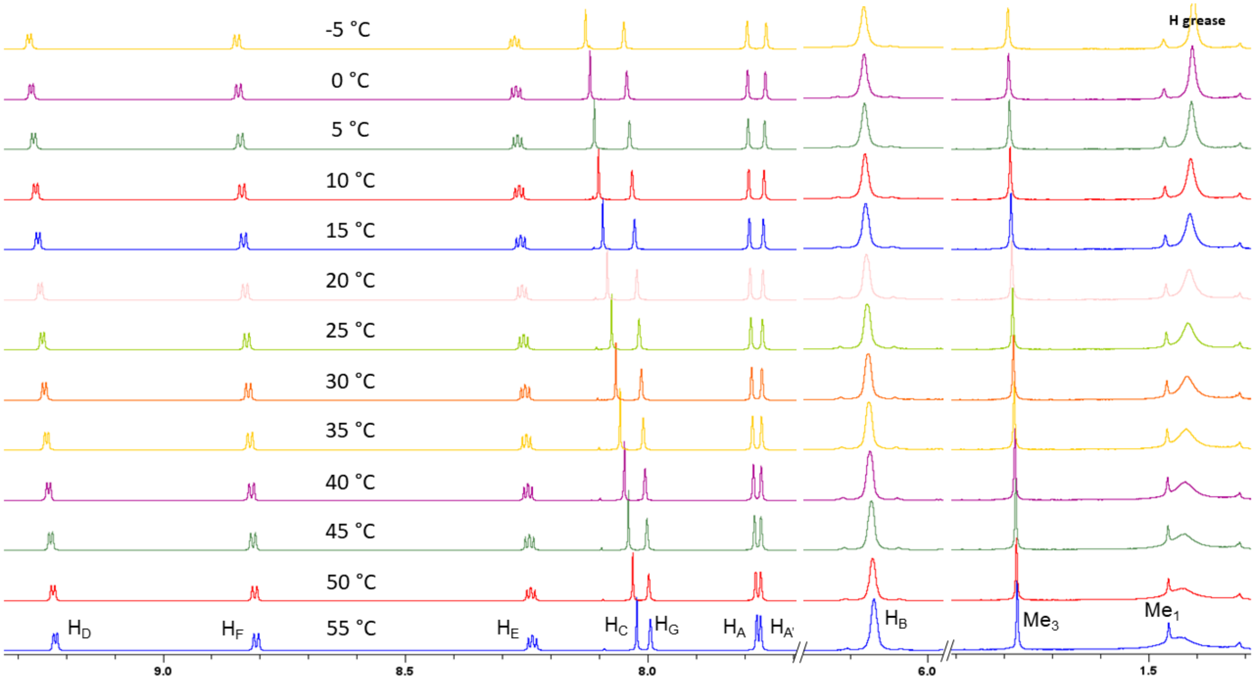

Figure S14. Variable temperature ^{1}H NMR spectra (850 MHz, CD_3OD) of bowl **1**$^{6+}$.

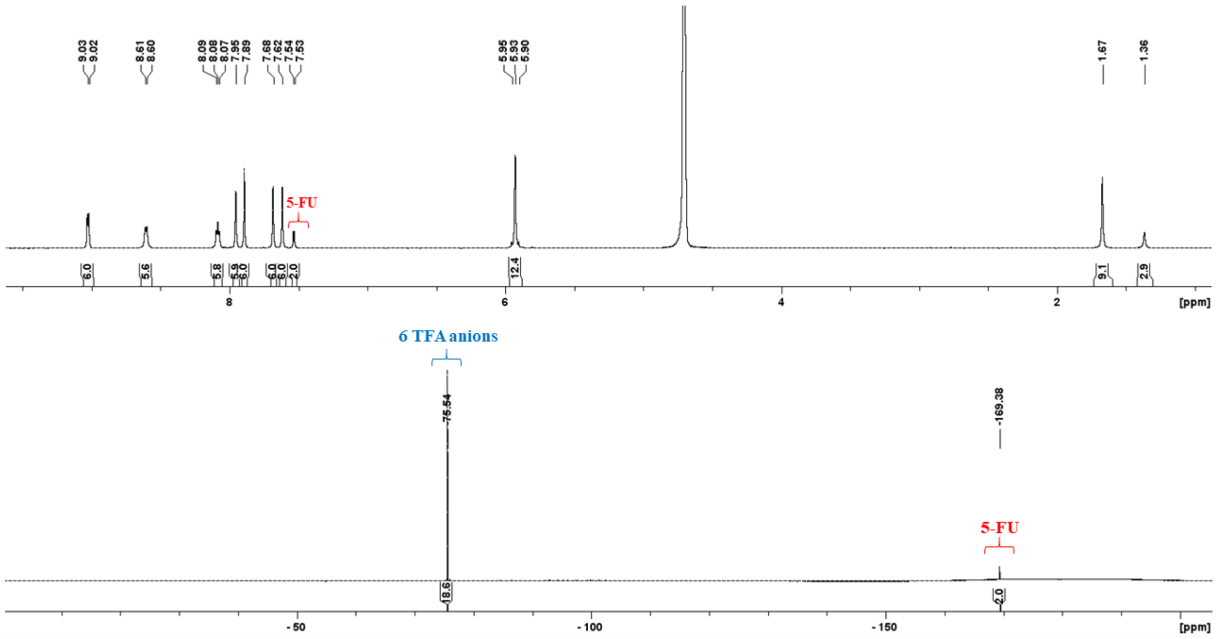

Figure S15. ^{1}H NMR (600 MHz, D_2O) and ^{19}F NMR (600 MHz, D_2O) spectra bowl $\mathbf{1}^{6+}$ with 5-flurouracil (5-FU) as an internal standard. From the signal integration, we determined that one bowl $\mathbf{1}^{6+}$ holds six trifluoroacetate anions (i.e. all bromide anions from the synthetic procedure are being replaced during HPLC purification). From ^{1}H-NMR spectrum, the molar ratio of $\mathbf{1}^{6+}$ to 5-FU is 1 to 2. ^{19}F NMR spectrum of the same mixture indicates the presence of 18 fluorides from TFA with respect to two fluorides from 5-FU (each 5-FU has one fluoride). It follows that there are 6 TFA anions for one bowl $\mathbf{1}^{6+}$.

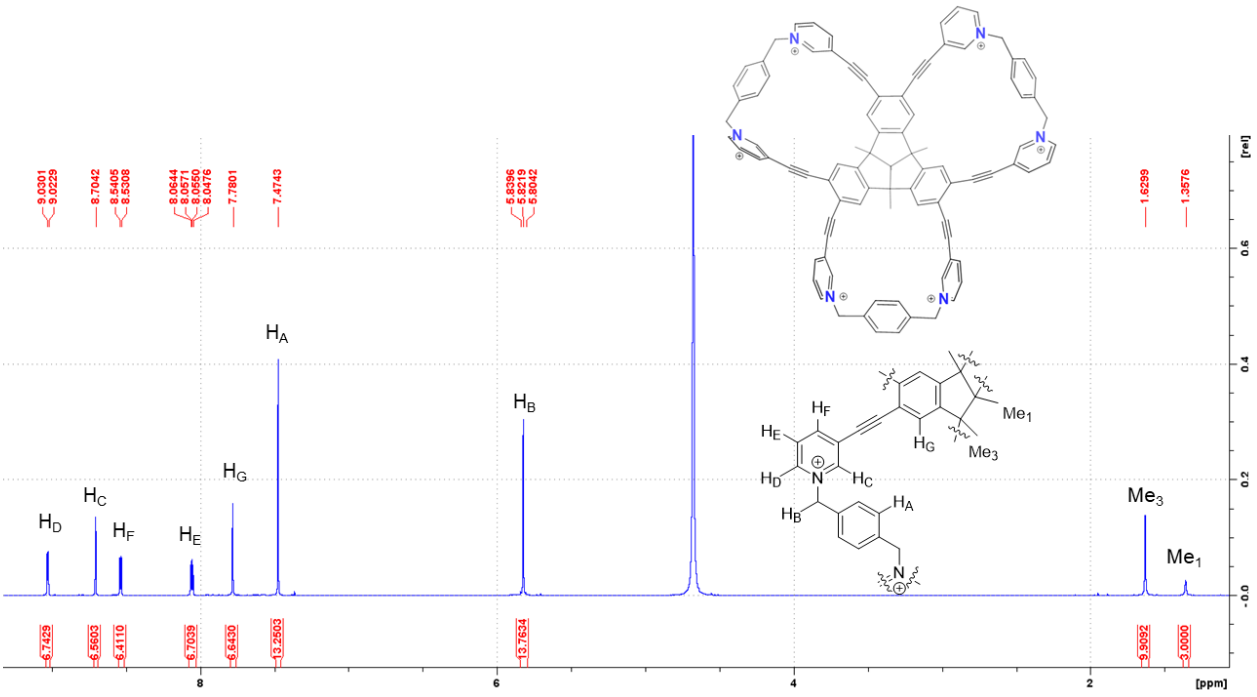

Figure S16. ^{1}H NMR spectrum (600 MHz, D_2O, 298K) of bowl $\mathbf{2}^{6+}$.

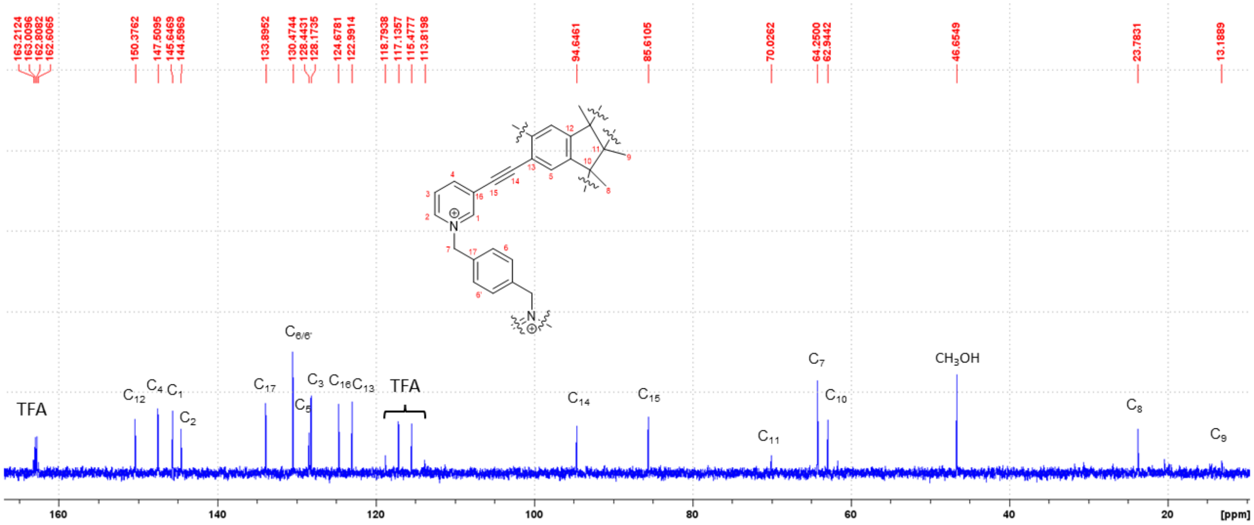

Figure S17. ^{13}C NMR spectrum (176 MHz, D_2O, 298K) of bowl $\mathbf{2}^{6+}$.

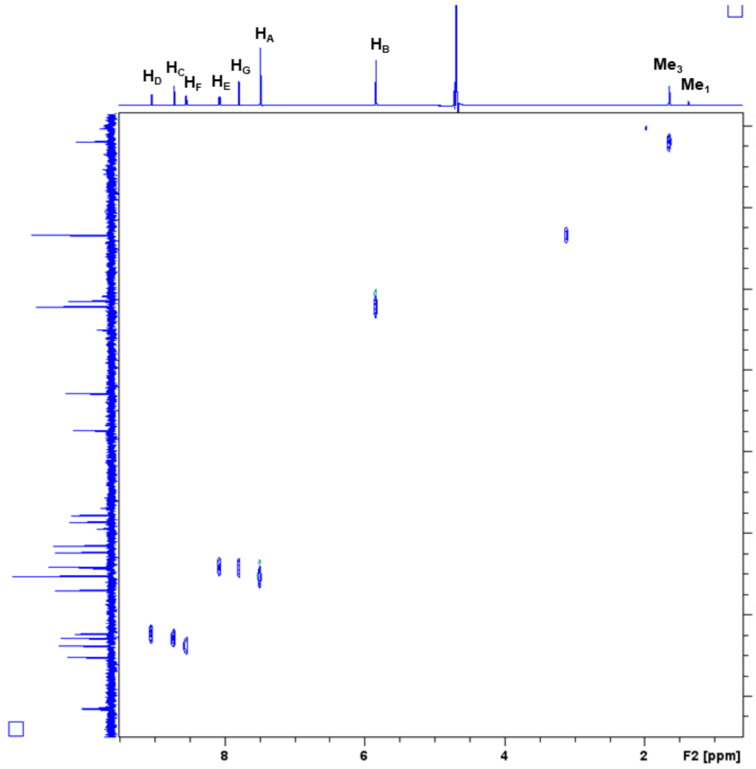

Figure S18. ^{1}H-^{13}C HSQC spectrum (700 MHz, D_2O, 298K) of bowl $\mathbf{2}^{6+}$.

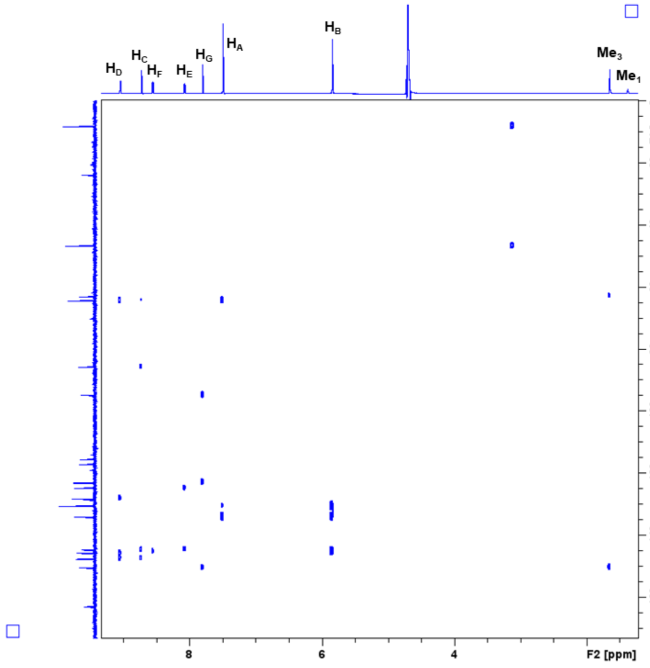

Figure S19. ^{1}H-^{13}C HMBC spectrum (700 MHz, D_2O, 298K) of bowl **2**$^{6+}$.

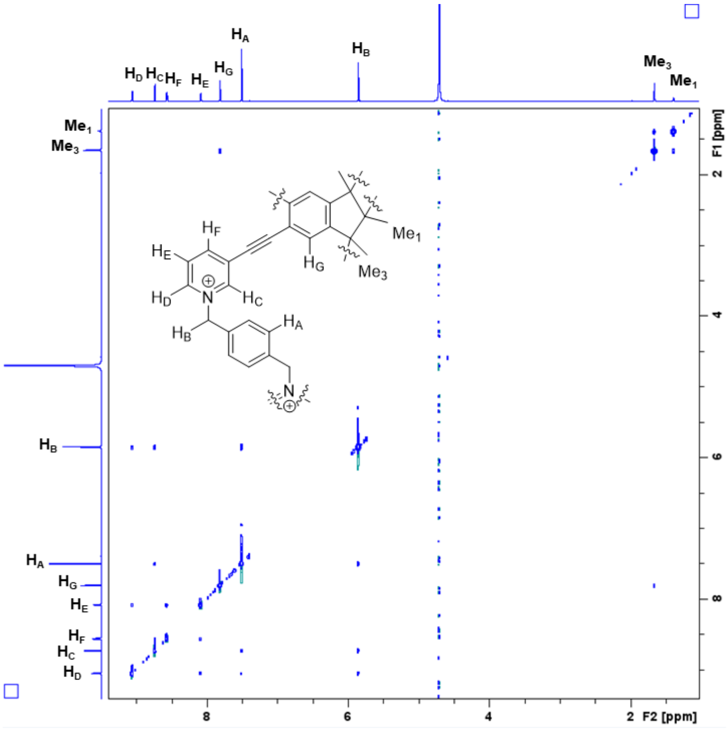

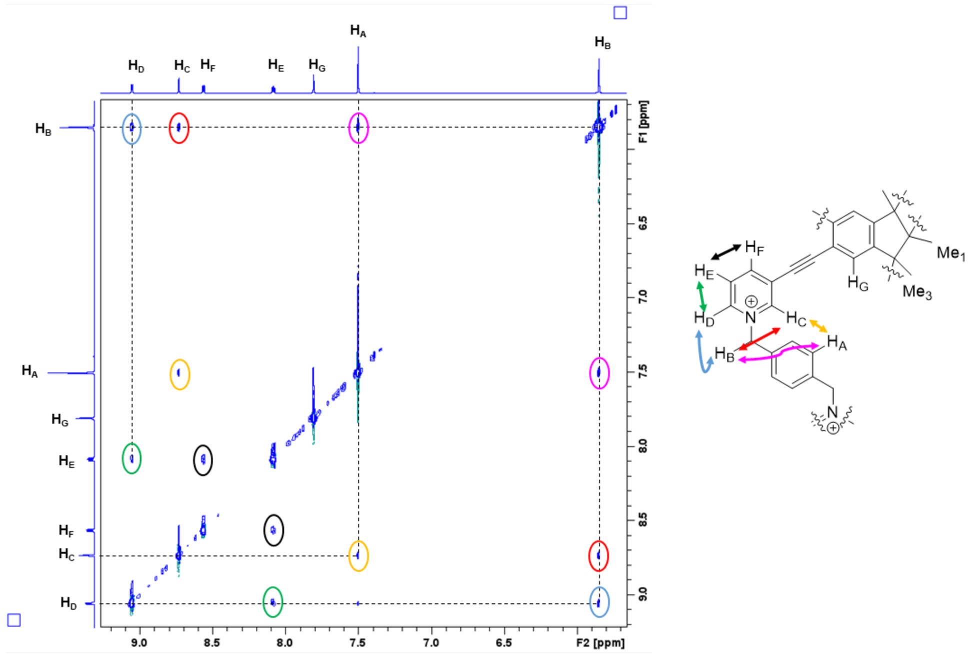

Figure S20. Two views of ^{1}H-^{1}H NOESY spectrum (850 MHz, D_2O, 298K) of bowl $\mathbf{2}^{6+}$ with the assigned cross peaks.

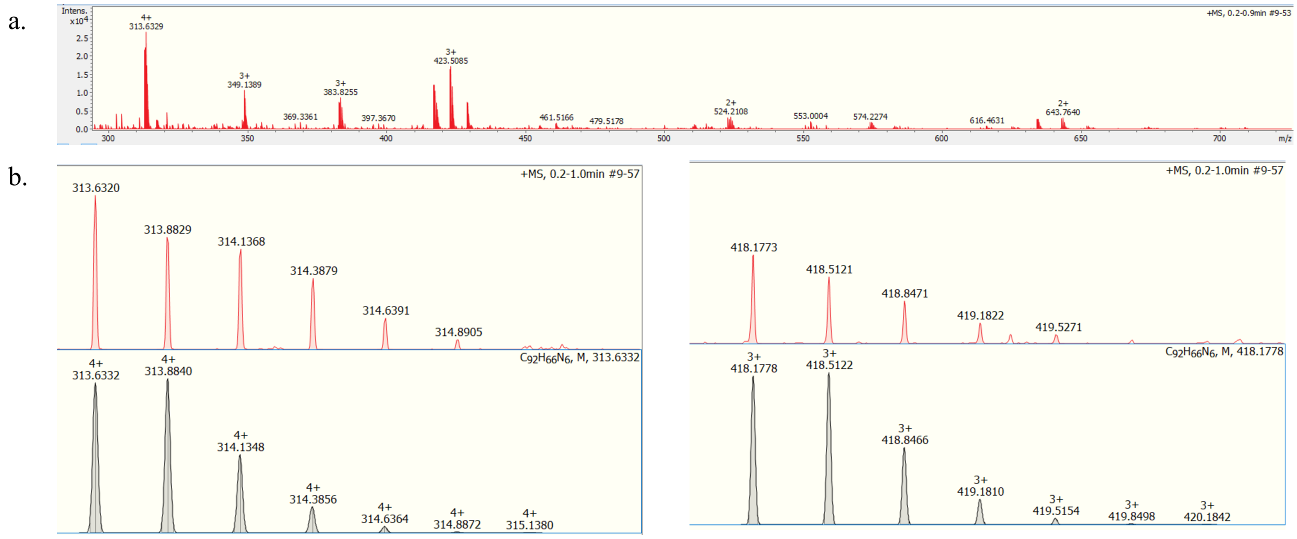

Figure S21. (a) Positive mode HRMS-ESI of bowl $\mathbf{2}^{6+}$ in a solution of ammonium bicarbonate buffer. (b) Segments of experimental (top) and simulated (bottom) HRMS-ESI of bowl $\mathbf{2}^{6+}$. The formation of $[\mathbf{2}]^{4+}$ cation with *m/z* calcd for $C_{92}H_{66}N_6^{4+}$ 313.6332 (bottom), found 313.6320 (top) and $[\mathbf{2}]^{3+}$ cation with *m/z* calcd for $C_{92}H_{66}N_6^{3+}$ 418.1778 (bottom), found 418.1773 (top, right spectra).

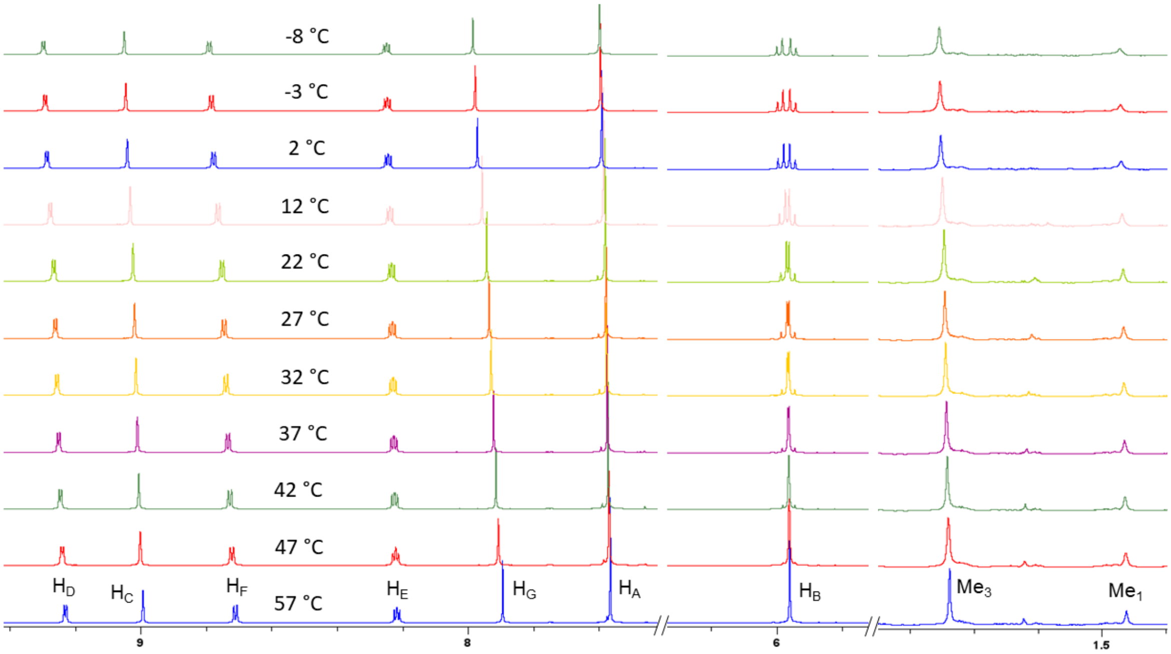

Figure S22. Variable temperature ^{1}H NMR spectra (850 MHz, CD_3OD) of bowl $\mathbf{2}^{6+}$.

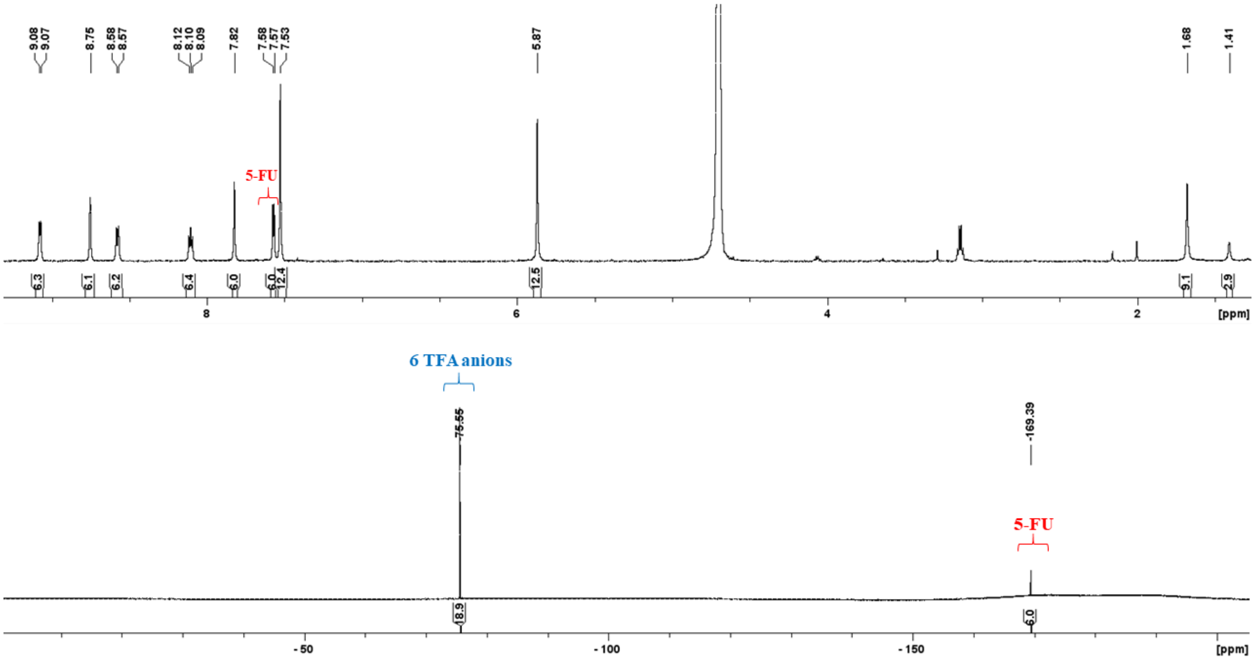

Figure S23. ^{1}H NMR (600 MHz, D_2O) and ^{19}F NMR (600 MHz, D_2O) spectra of bowl $\mathbf{2}^{6+}$ using 5-fluorouracil (5-FU) as an internal standard. From integration of signals, we determined that one bowl $\mathbf{2}^{6+}$ holds six trifluoroacetate anions (i.e., all bromide anions from the synthetic procedure are being replaced during HPLC purification). From ^{1}H-NMR spectrum, the molar ratio of $\mathbf{2}^{6+}$ to 5-FU is 1 to 6. ^{19}F NMR spectrum of the same mixture indicates the presence of 18 fluorides from TFA with respect to six fluorides from 5-FU (each 5-FU has one fluoride). It follows that there are 6 TFA anions for one bowl $\mathbf{2}^{6+}$.

Host-Guest Binding Studies

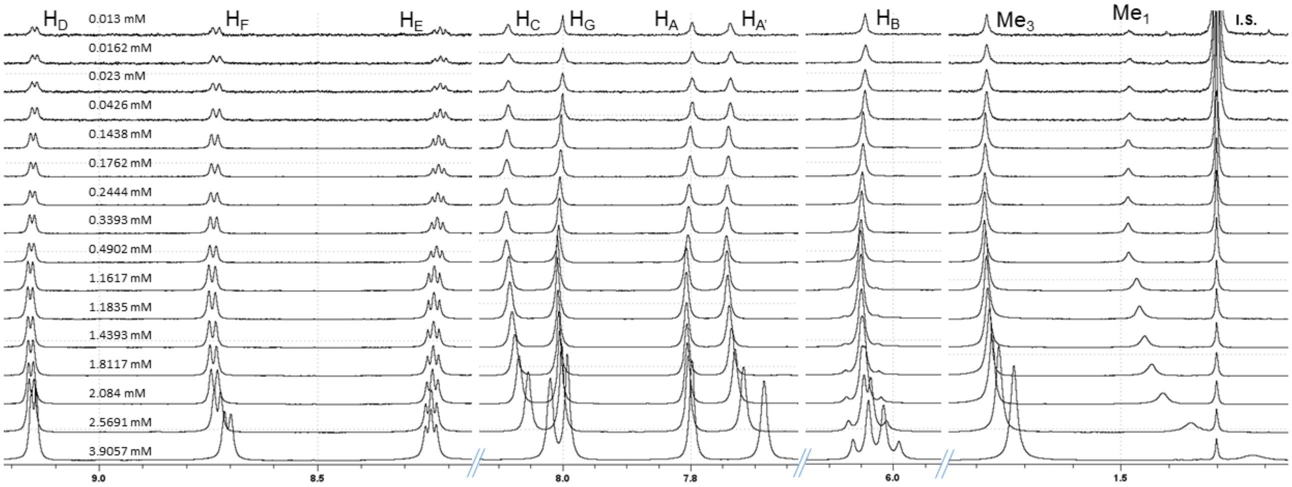

Figure S24. ^{1}H NMR spectra (850 MHz, 298K) of 0.01-3.91 mM solution of bowl $\mathbf{1}^{6+}$ in 30 mM phosphate buffer (10% D_2O in water, pH = 7.4); dimethyl malonate was used as internal standard.

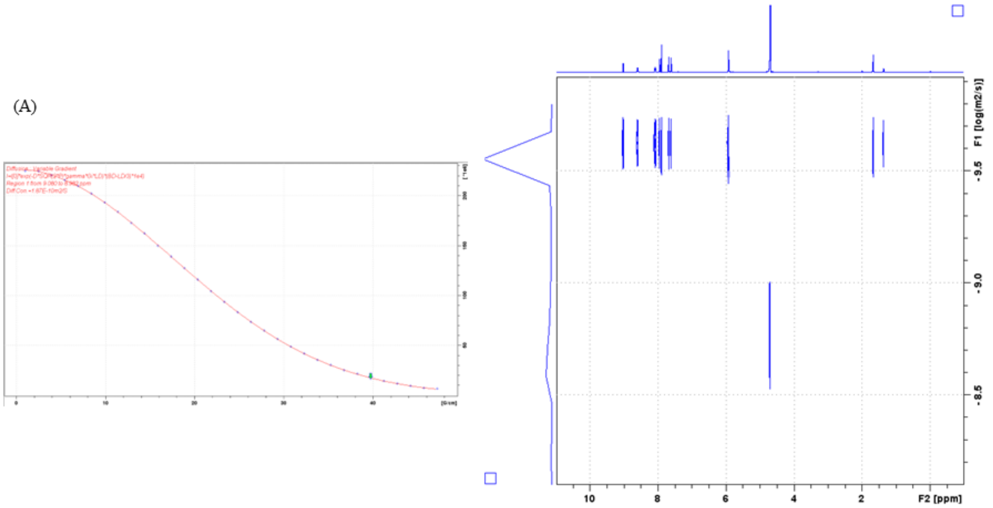

(B)

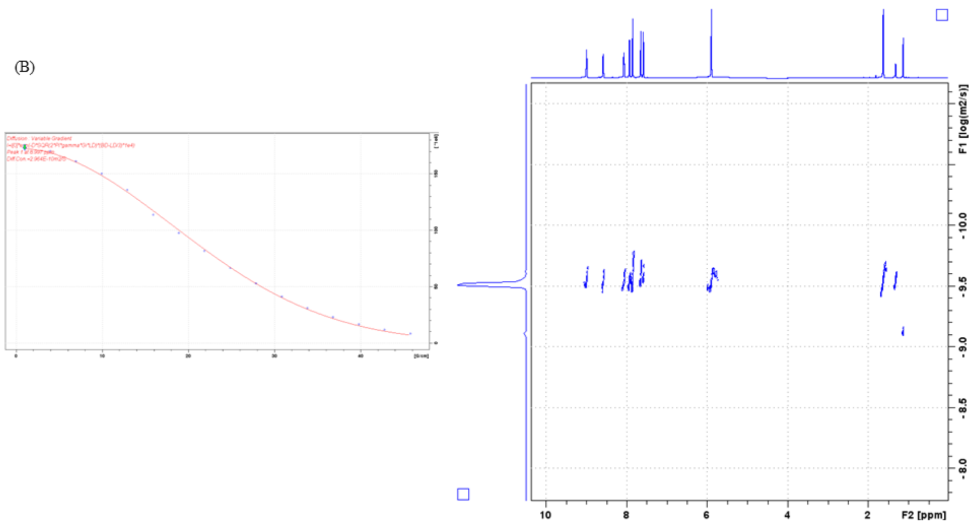
F1 [log(m2/s)]
-10.0
-9.5
-9.0
-8.5
-8.0
10
8
6
4
2
F2 [ppm]

(C)

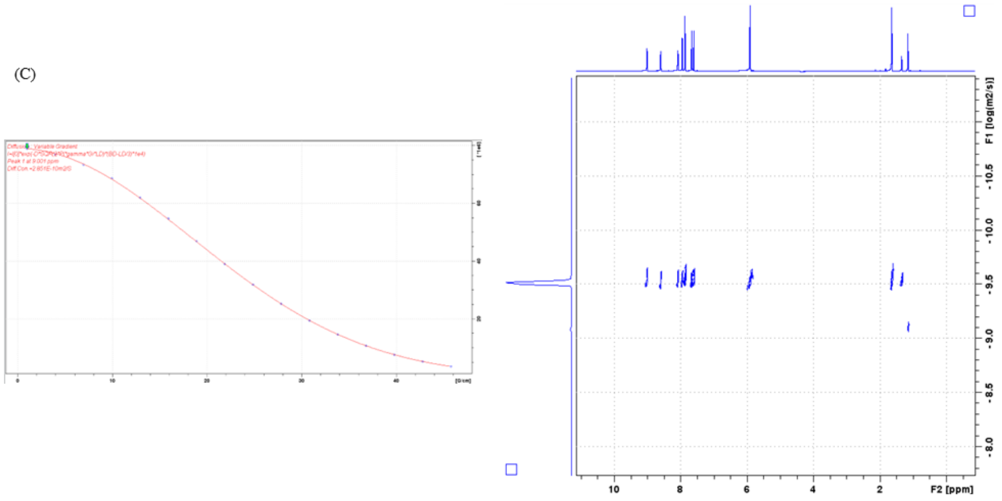
F1 [log(m2/s)]
-10.5
-10.0
-9.5
-9.0
-8.5
-8.0
10
8
6
4
2
F2 [ppm]

(D)

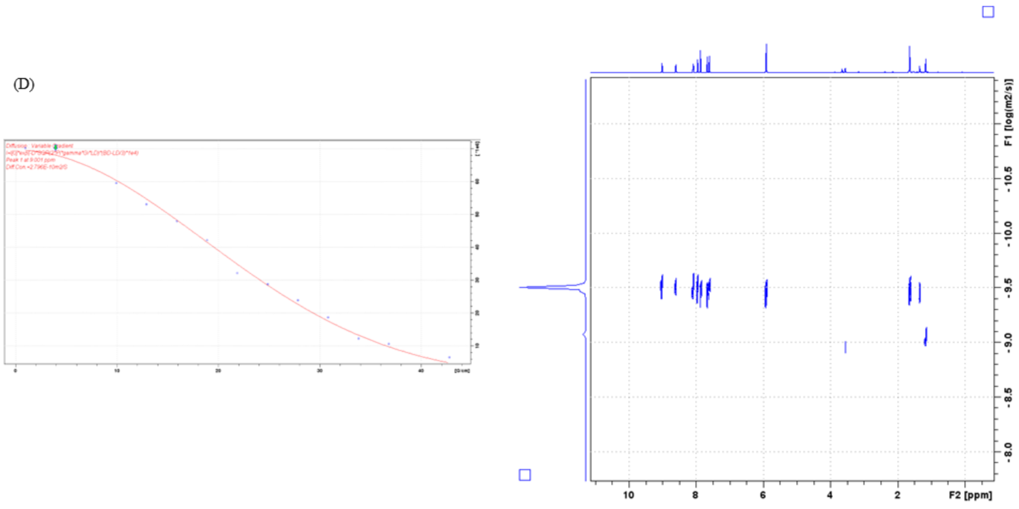
F1 [log(m2/s)]
-10.5
-10.0
-9.5
-9.0
-8.5
-8.0
10
8
6
4
2
F2 [ppm]

Figure S25. DOSY NMR spectra (800 MHz, 298 K) of variously concentrated bowl **1**$^{6+}$ in D_2O: (A) 2.1 mM, (B) 1.0 mM, (C) 0.5 mM and (D) 0.025 mM. Change in intensity of resonance corresponding to H_2 proton as a function of the field gradient *g* (G/cm) was obtained using the pulse field gradient stimulated echo sequence with bipolar gradient pulse pair, 1 spoil gradient, stebpgp1s19 pulse sequence. The data was fit to the Stejskal-Tanner equation to give apparent diffusion coefficient *D* (m^2 /s). Hydrodynamic radii (r_H) were then calculated using the Stokes-Einstein equation with the viscosity of D_2O being η = 0.00891Pa s at 298K. Computed *D* and r_H are as follows: (A) $D = 1.90\times10^{-10}$ m^2s^{-1} and $r_H = 1.299$ nm (B) $D = 2.964\times10^{-10}$ m^2s^{-1} and $r_H = 0.827$ nm, (C) $D = 2.851\times10^{-10}$ m^2s^{-1} and $r_H = 0.86$ nm and (D) $D = 2.796\times10^{-10}$ m^2s^{-1} and $r_H = 0.877$ nm. Each sample contained dimethyl malonate as internal standard.

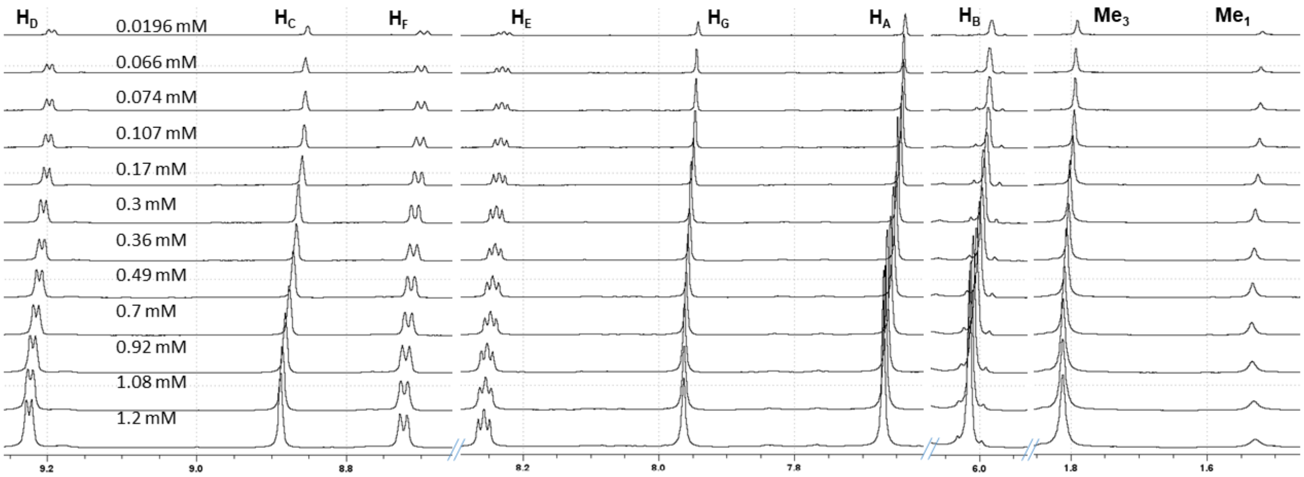

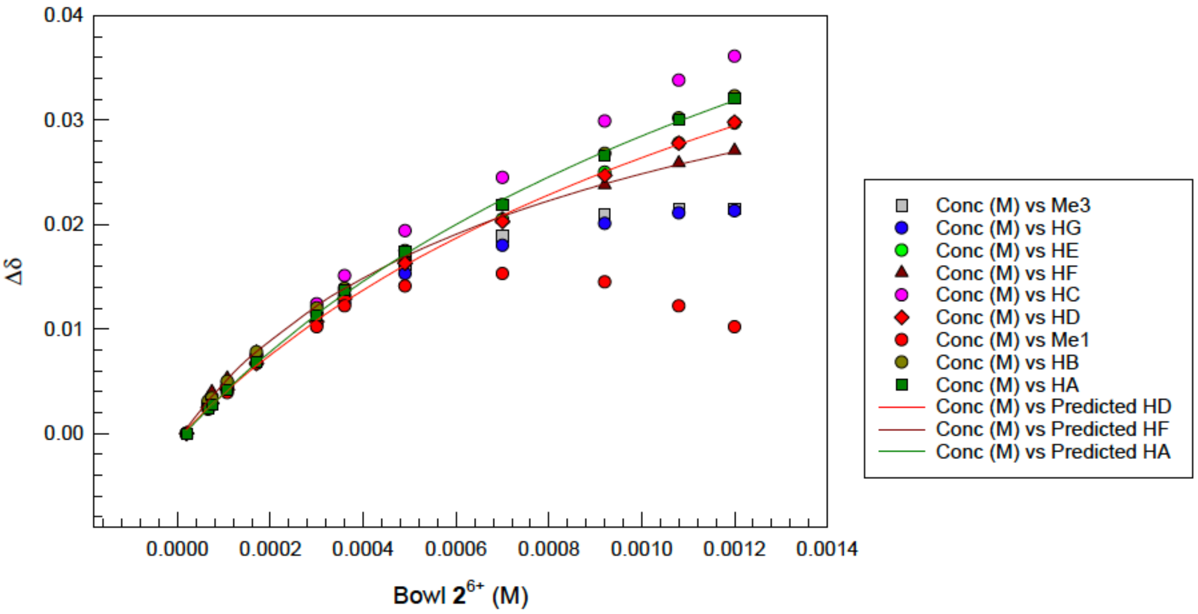

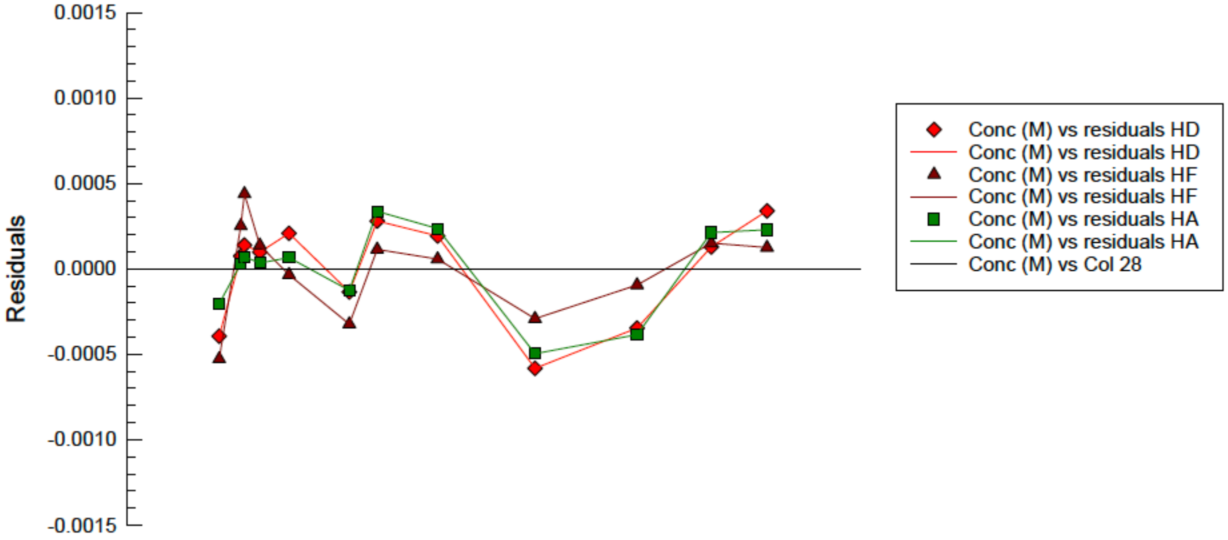

Figure S26. (Top) ^{1}H NMR spectra (850 MHz, 298K) of 0.0196-1.2 mM solution of bowl $\mathbf{2}^{6+}$ in 30 mM phosphate buffer (10% D_2O in water, pH = 7.4); dimethyl malonate was used as internal standard. (Bottom) A change in the chemical shift of protons from bowl $\mathbf{2}^{6+}$ as a function its concentration in water (30 mM PBS buffer at pH = 7.4). A change in chemical shift of H_D, H_F and H_A from $\mathbf{2}^{6+}$ with concentration fit well to dimerization model (SigmaPlot) with a random distribution of residuals with K = 212, 485 and 200 M^{-1}.

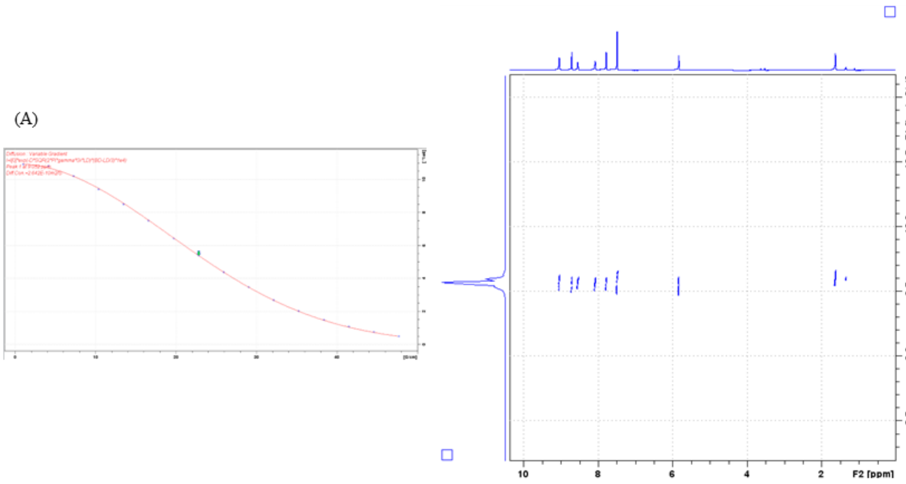

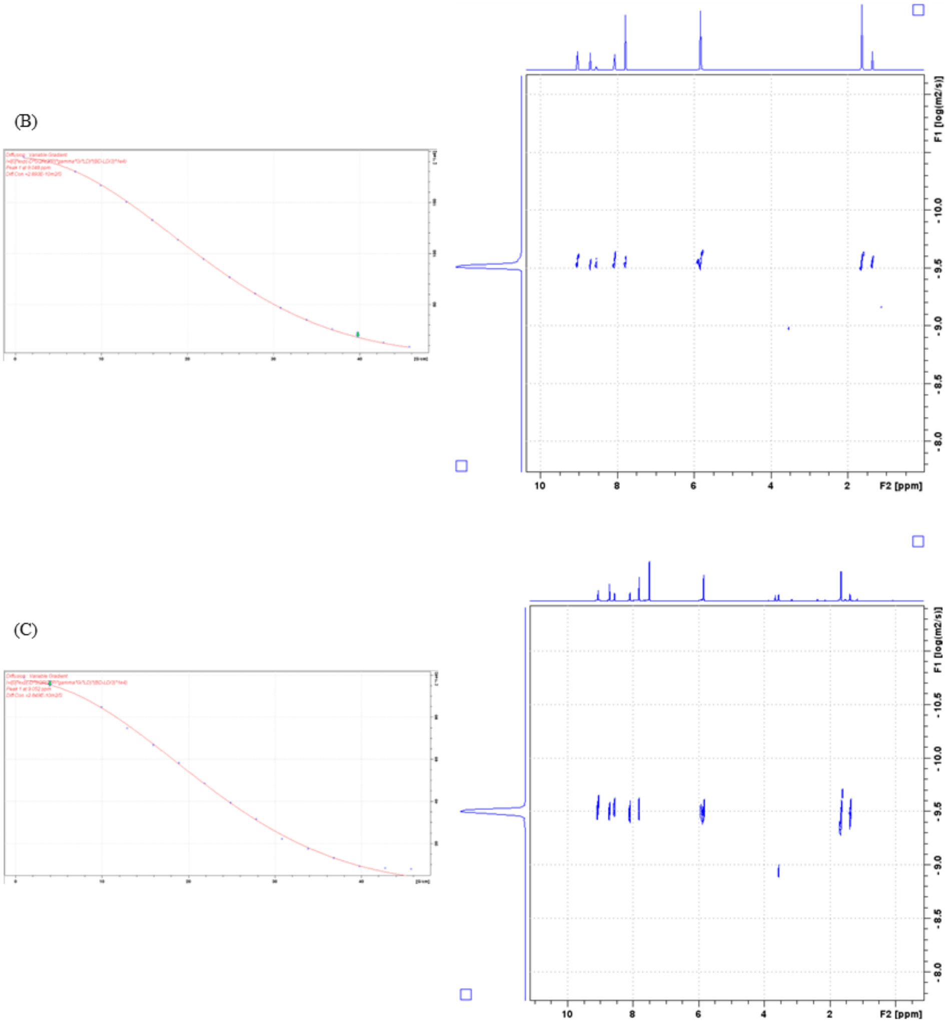

Figure S27. DOSY NMR spectra (800 MHz, 298 K) of variously concentrated $\mathbf{2}^{6+}$ in D_2O: (A) 1.2 mM, (B) 0.44 mM and (C) 0.023 mM. Change in intensity of resonance corresponding to H_2 proton as a function of the field gradient g (G/cm) was obtained using the pulse field gradient stimulated echo sequence with bipolar gradient pulse pair, 1 spoil gradient, stebpgp1s19 pulse sequence. The data was fit to the Stejskal-Tanner equation to give diffusion coefficient D (m^2 /s). Hydrodynamic radii (r_H) were calculated using the Stokes-Einstein equation with the viscosity of D_2O being $\eta = 0.00891$Pa s at 298K. Computed D and r_H are as follows: (A) $D = 2.642\times10^{-10}$ m^2s^{-1} and $r_H = 0.93$ nm (B) $D = 2.893\times10^{-10}$ m^2s^{-1} and $r_H = 0.85$ nm and (C) $D = 2.849\times10^{-10}$ m^2s^{-1} and $r_H = 0.86$ nm. Each sample contained dimethyl malonate as internal standard.

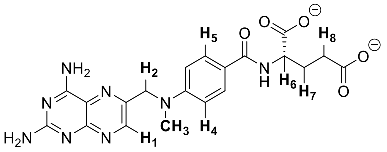

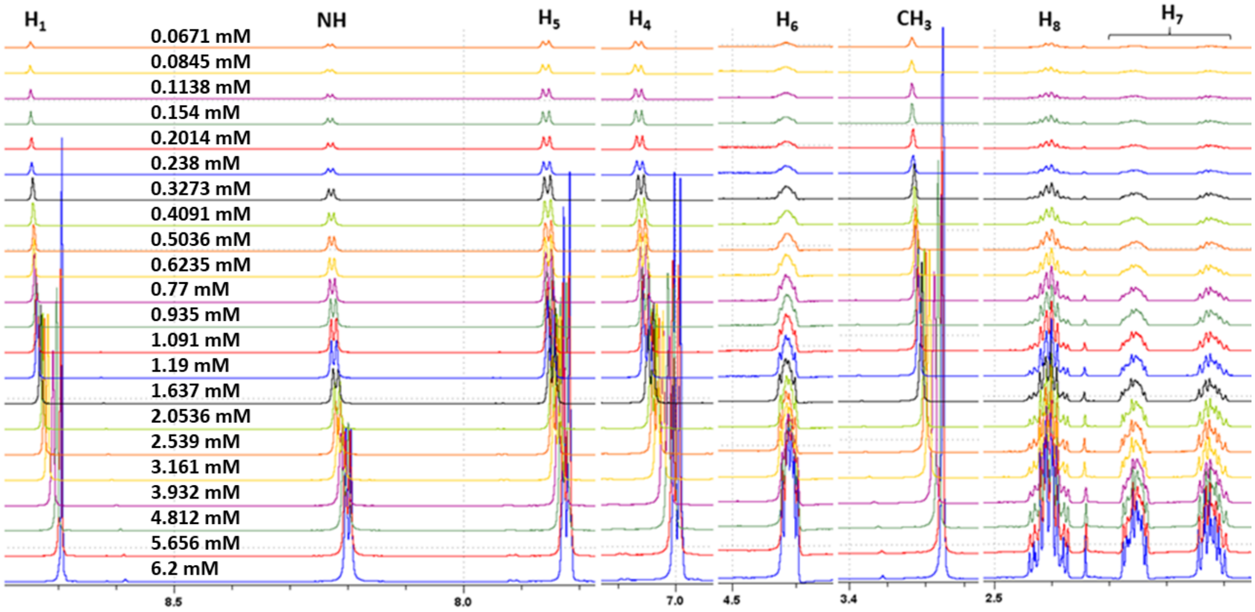
H1
NH
H5
H4
H6
CH3
H8
H7
0.0671 mM
0.0845 mM
0.1138 mM
0.154 mM
0.2014 mM
0.238 mM
0.3273 mM
0.4091 mM
0.5036 mM
0.6235 mM
0.77 mM
0.935 mM
1.091 mM
1.19 mM
1.637 mM
2.0536 mM
2.539 mM
3.161 mM
3.932 mM
4.812 mM
5.656 mM
6.2 mM
8.5
8.0
7.0
4.5
3.4
2.5

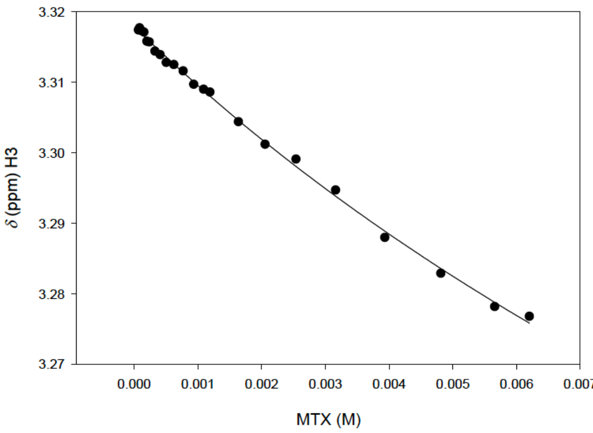
δ (ppm) H3
MTX (M)

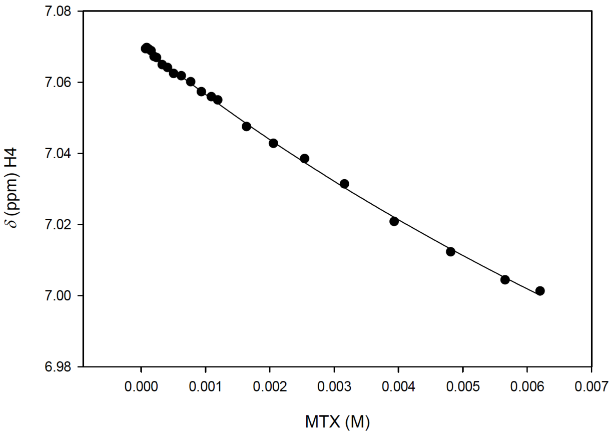
δ (ppm) H4
MTX (M)

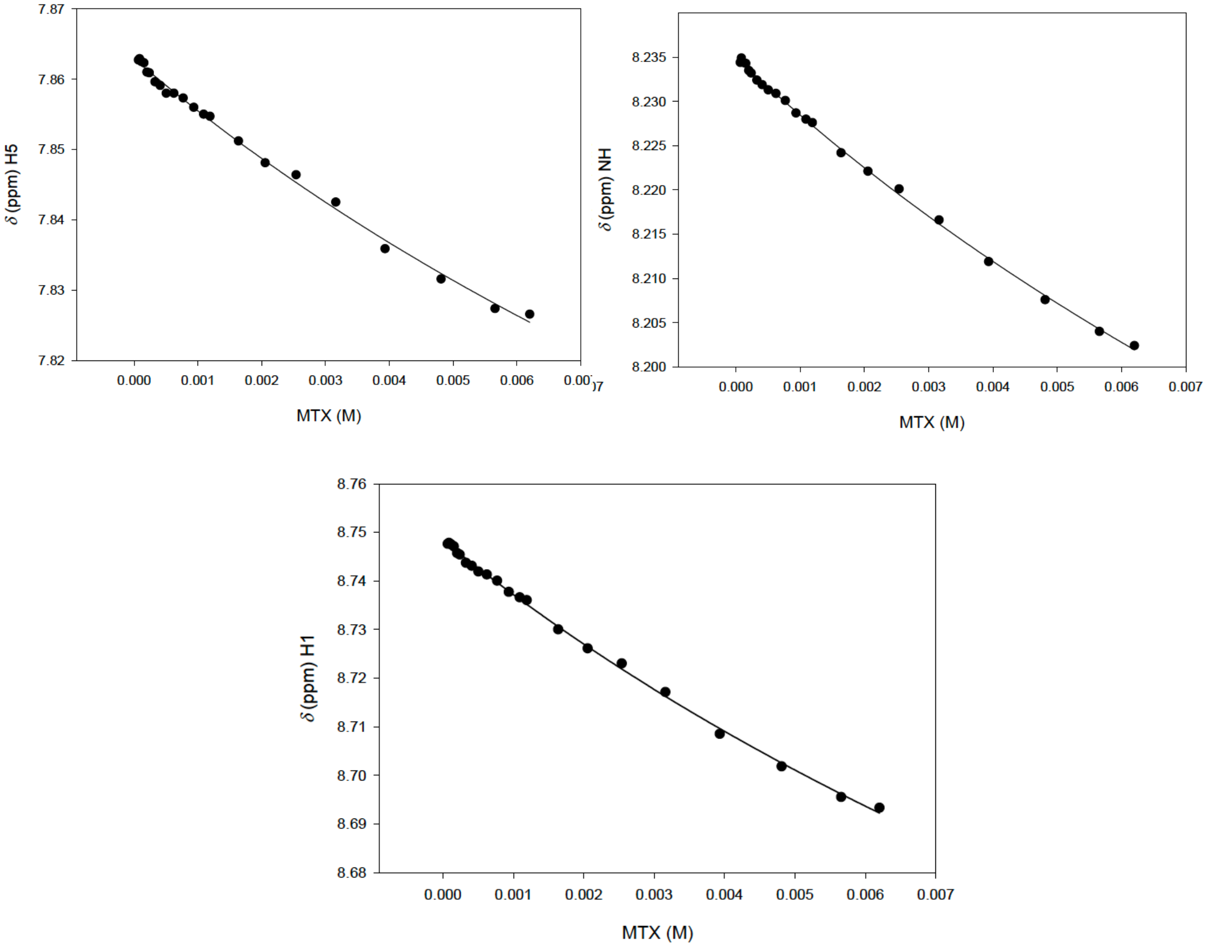

Figure S28. ^{1}H NMR spectra (850 MHz, D_2O, 298 K) of 0.07-6.20 mM solutions of MTX^{2-} in 30 mM phosphate buffer (10% D_2O in water, pH = 7.4). A change in chemical shift of H_3, H_4 , H_5, NH and H_1 from **MTX**$^{2-}$ with concentration fit well to dimerization model (SigmaPlot) with a random distribution of residuals and K = 12.7, 12.3, 12.3, 12.1 and 12.8 M^{-1}.

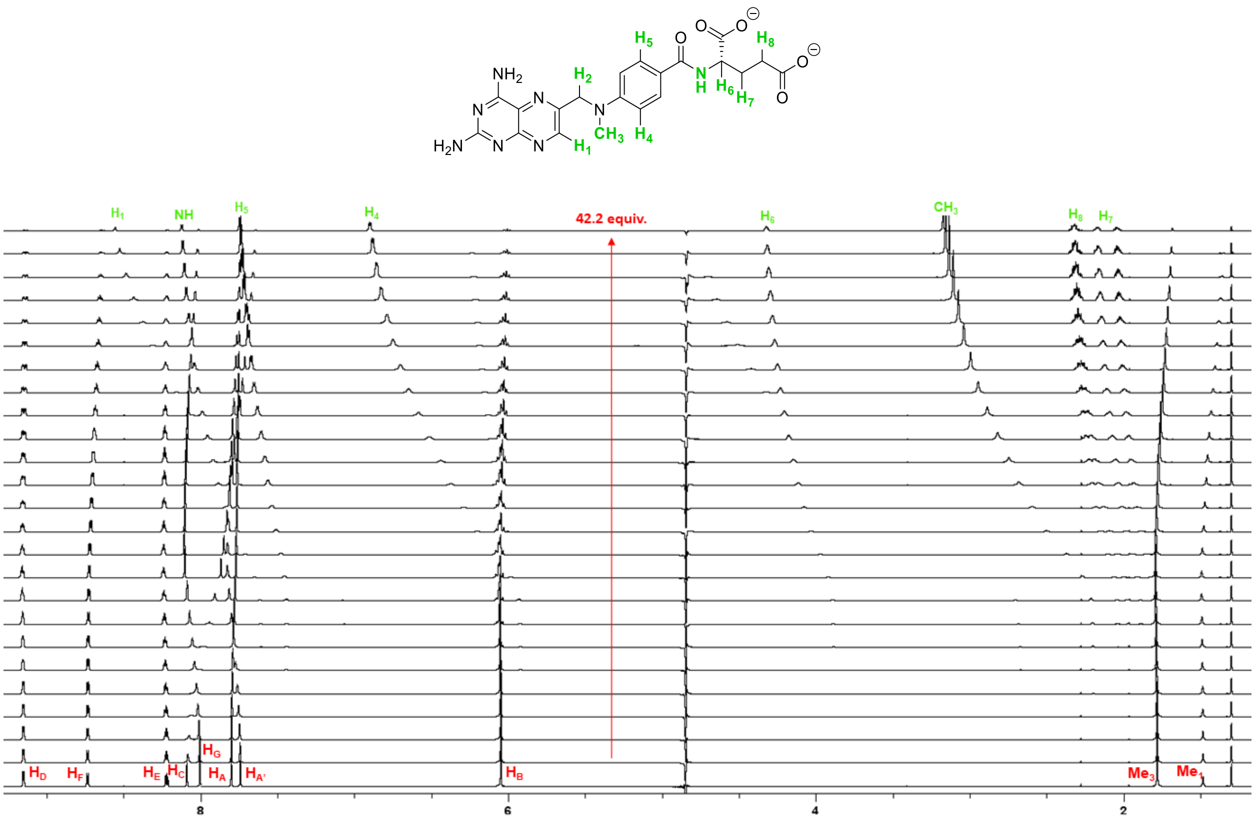

Figure S29. ^{1}H NMR spectra (850 MHz, D_2O, 298 K) of 88 μM of bowl $\mathbf{1}^{6+}$ in 30 mM phosphate buffer (10% D_2O in water, pH = 7.4) obtained upon an incremental addition of 2.5 mM methotrexate in 30 mM phosphate buffer (pH = 7.4); see Figure S27 for additional details.

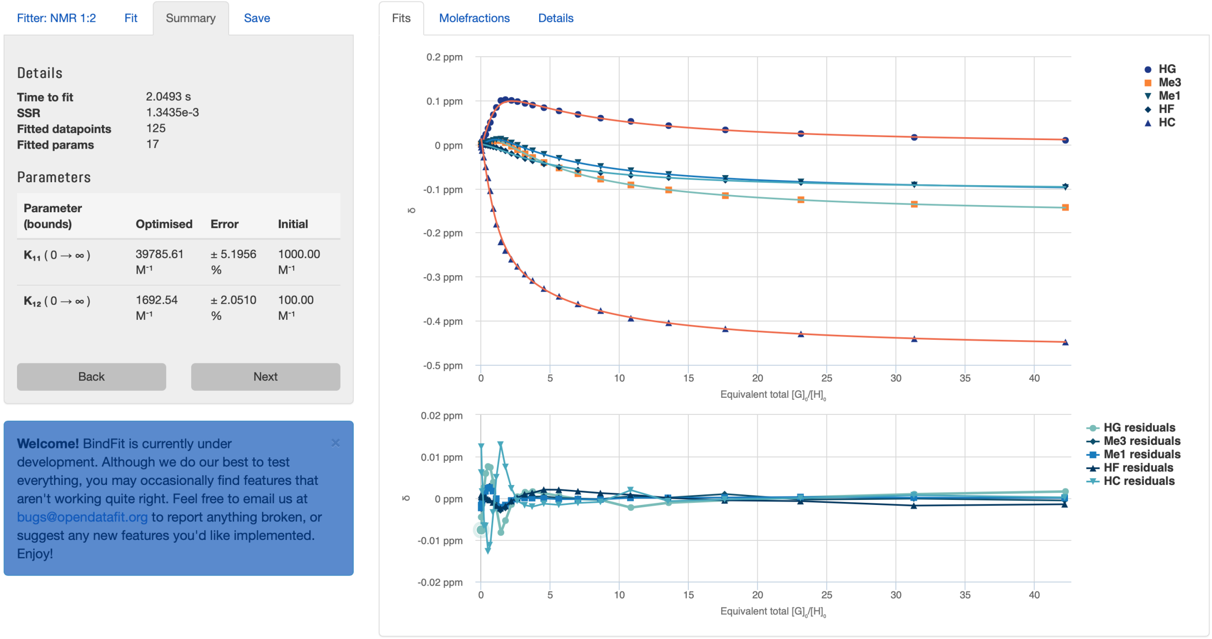

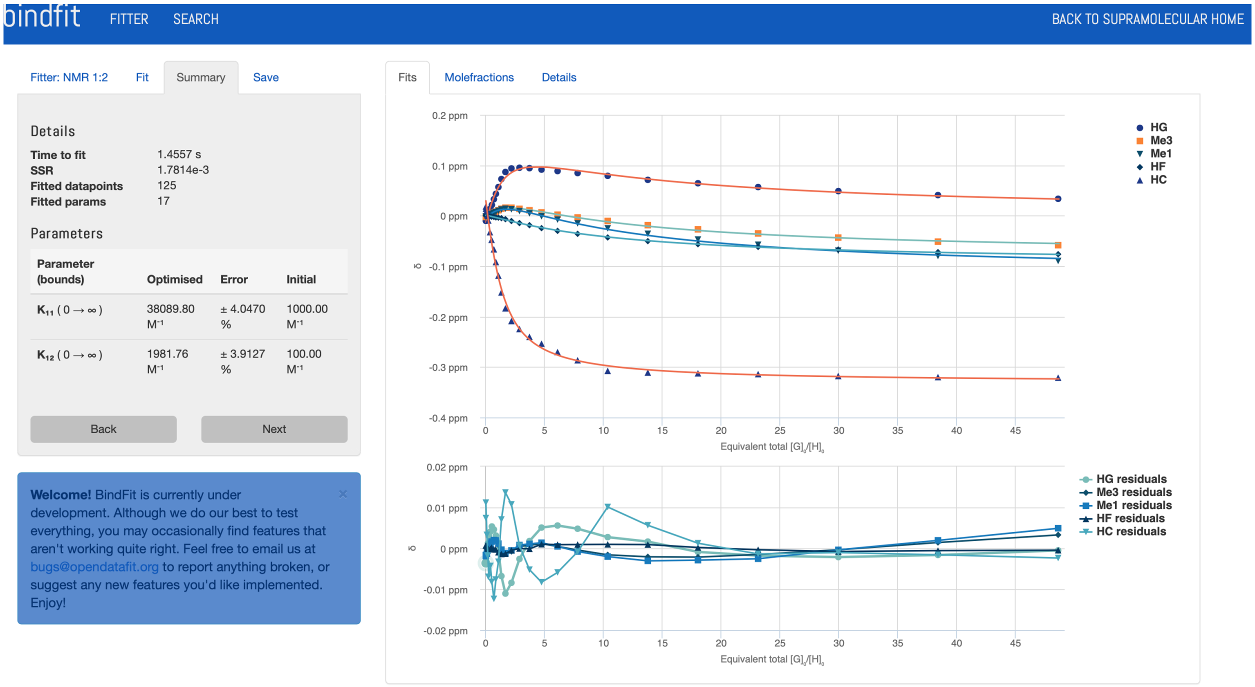

Figure S30. A change in ^{1}H NMR chemical shift of five resonances from bowl $\mathbf{1}^{6+}$ as a function of increasing concentration of methotrexate were subjected to nonlinear least-square regression analysis using 1:2 binding model (see: supramolecular.org). From four independent measurements (only two representative cases are shown above) we obtained: (a) K_1 = 39786 M^{-1} and K_2 = 1693 M^{-1}, (b) K_1 = 85931 M^{-1} and K_2 = 1552 M^{-1}, (c) K_1 = 11871 M^{-1} and K_2 = 2221 M^{-1} and (d) K_1 = 38090 M^{-1} and K_2 = 1982 M^{-1}.

a.

b.

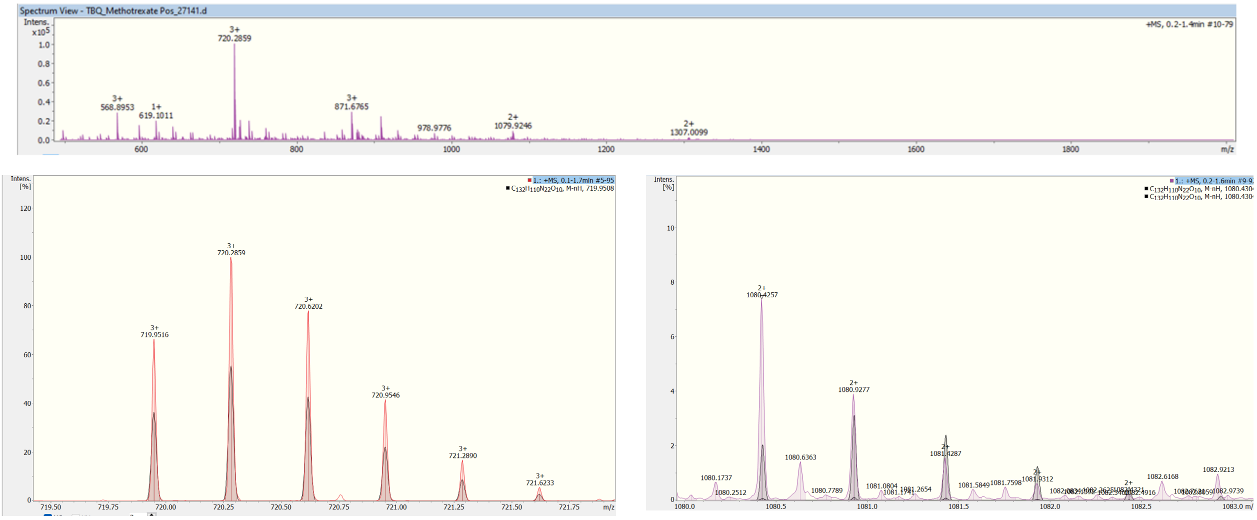

Figure S31. (a) Positive mode HRMS-ESI of methotrexate and $\mathbf{1}^{6+}$ in ammonium bicarbonate buffer. (b) Segments of experimental (top) and simulated (bottom) HRMS-ESI showing peaks corresponding to ternary [MTX$_2$⊂**1**]$^{2+}$; note the formation of [MTX$_2$⊂**1**-H]$^{3+}$ cation with *m/z* calcd for $C_{132}H_{109}N_{22}O_{10}^{3+}$ 719.9508, found 719.9516 and [MTX$_2$⊂**1**-H]$^{2+}$ cation with *m/z* calcd for $C_{132}H_{109}N_{22}O_{10}^{2+}$ 1080.430, found 1080.4257.

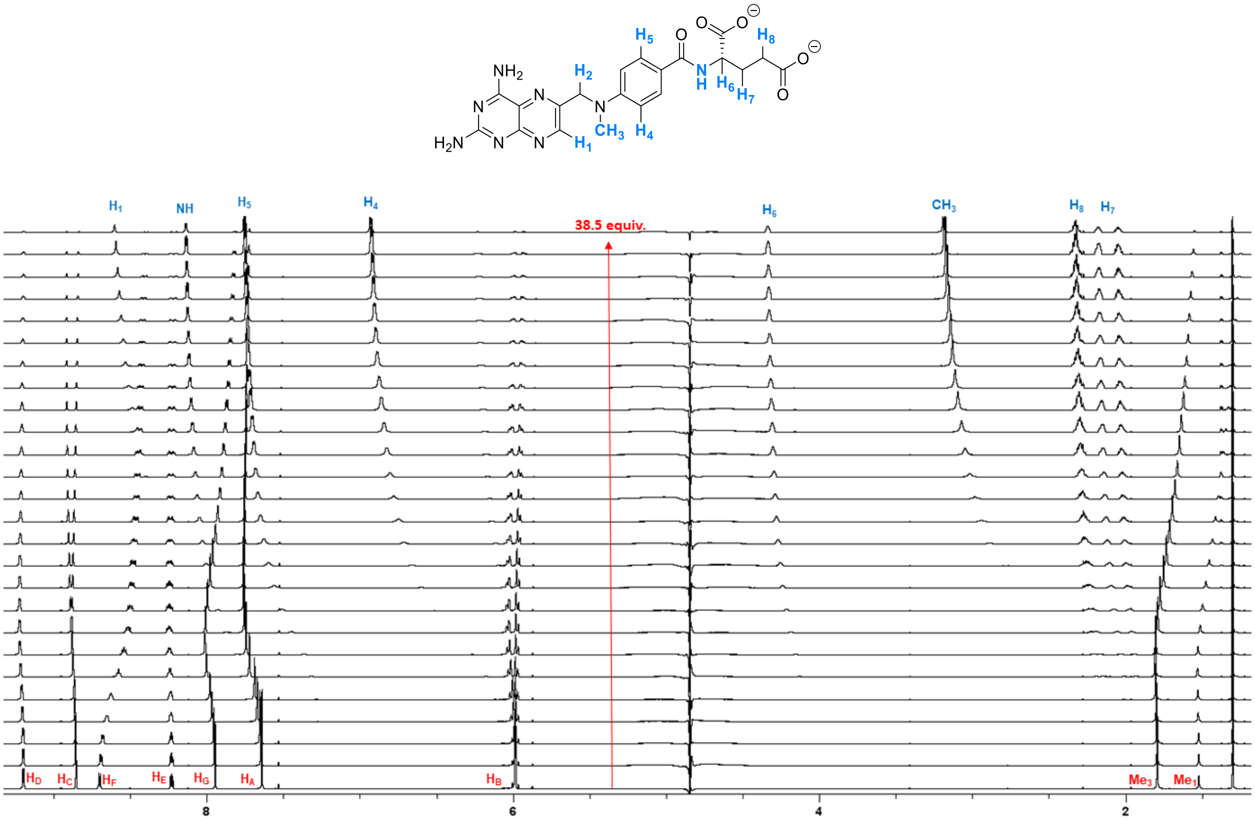

Figure S32. ^{1}H NMR spectra (850 MHz, D_2O, 298 K) of 90 μM solution of bowl $\mathbf{2}^{6+}$ in 30 mM phosphate buffer (10% D_2O in water, pH ~ 7.4) obtained upon an incremental addition of 3.6 mM methotrexate in 30 mM phosphate buffer (30 mM, 10% D_2O in water, pH ~ 7.4). Each sample contained dimethyl malonate as internal standard; see Figure S33 for additional details.

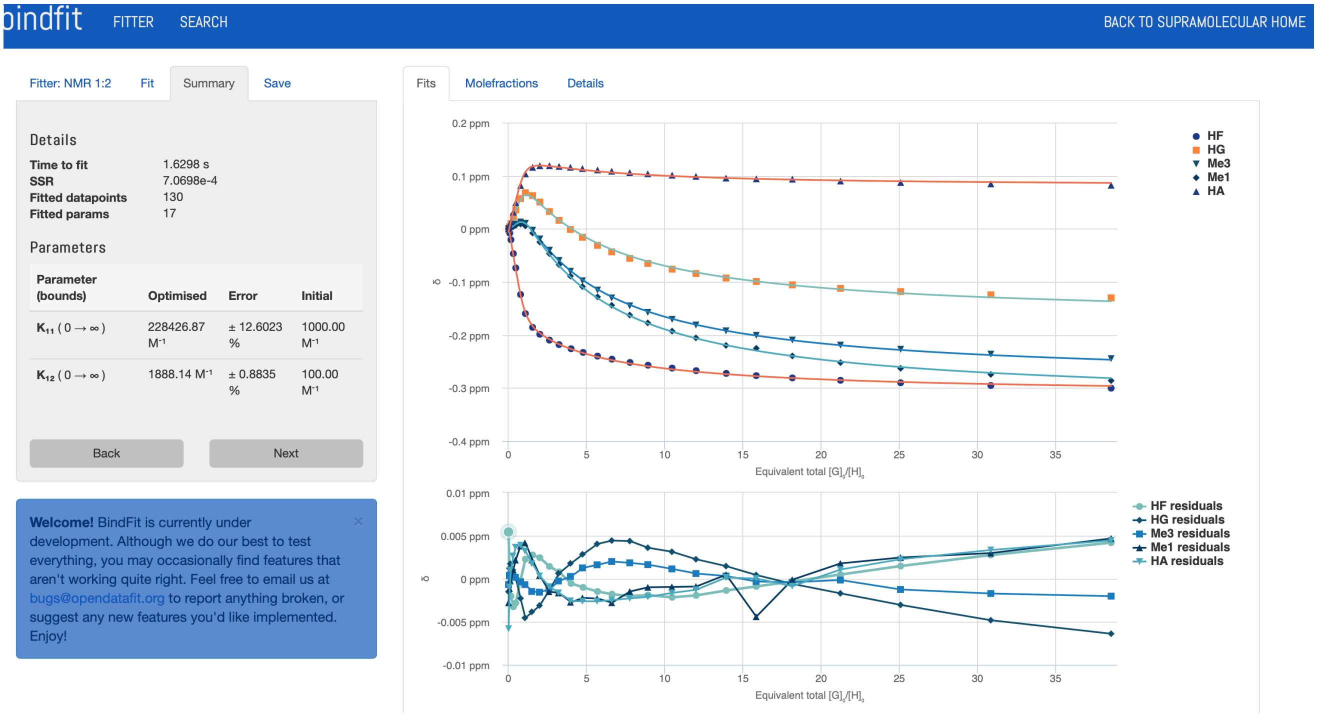

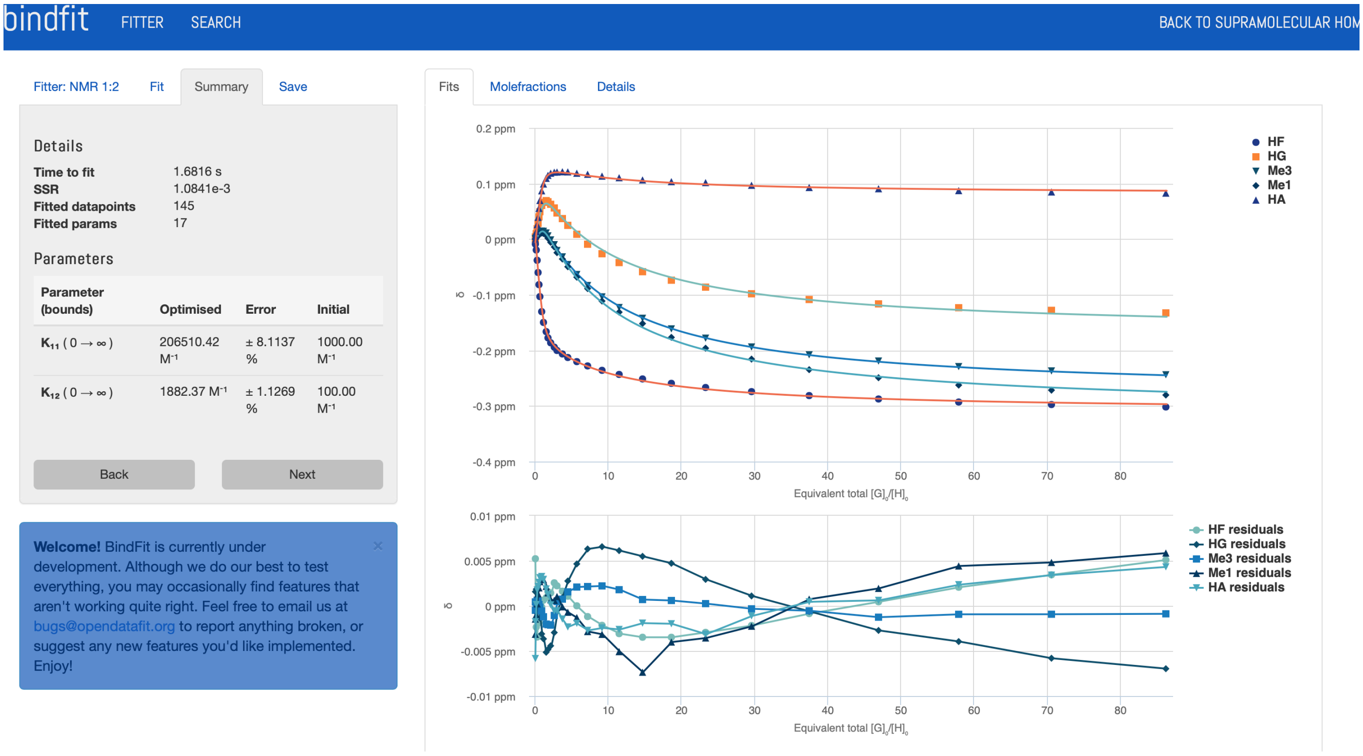

Figure S33. Changes in ^{1}H NMR chemical shifts of four resonances from bowl **2**$^{6+}$ (44 μM) obtained as a function of increasing concentration of methotrexate were subjected to nonlinear least square analysis using 1:2 binding model (see: supramolecular.org) giving $K_1 = 228427$ M^{-1}, $K_2 = 1888$ M^{-1} and $K_1 = 206510$ M^{-1}, $K_2 = 1882$ M^{-1}.

a.

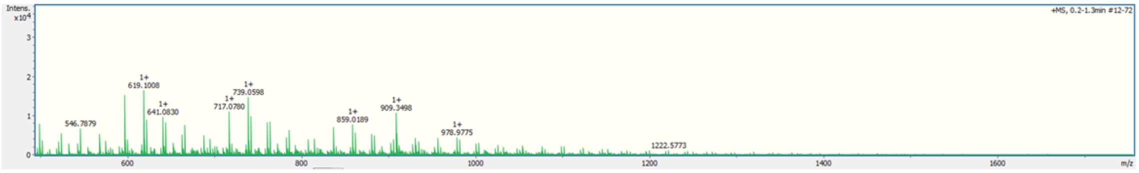

b.

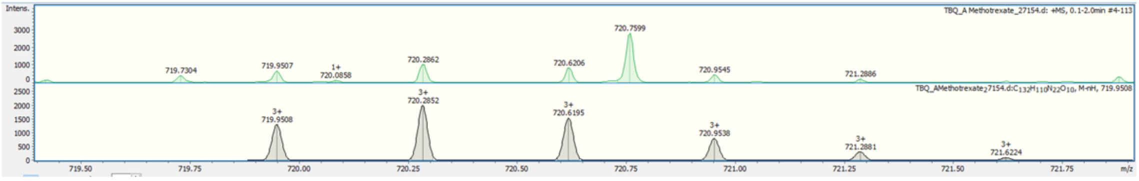

Figure S34. (a) Positive mode HRMS-ESI of methotrexate and $\mathbf{2}^{6+}$ in ammonium bicarbonate buffer. (b) Segments of experimental (top) and simulated (bottom) HRMS-ESI showing peaks from ternary $[MTX_2{\subset}\mathbf{2}]^{2+}$; note the formation of $[MTX_2{\subset}\mathbf{2}\text{-}H]^{3+}$ cation with *m*/*z* calcd for $C_{132}H_{109}N_{22}O_{10}^{3+}$ 719.9508 (bottom), found 719.9507 (top).

Computational Studies

General Notes for Molecular Dynamics and DFT Calculations: In order to investigate molecular bowls $\mathbf{1}^{6+}$ and $\mathbf{2}^{6+}$, the conformational flexibility of both bowls was investigated using a combination of conformational searches in Schrodinger's Macromodel,[1] geometry optimization in Gaussian 16[2] and molecular dynamics simulations using AMBER.[3] All geometry optimizations were completely at the B3LYP/6-31+G(d) level of theory with PCM water solvation. Frequency calculations were carried out for all final optimized molecules, although for some conformations of each bowl small imaginary frequencies remained (1-30 cm^{-1}). As a prerequisite for any MD simulation of a non-biological molecule, a single-point energy and electrostatic potential (ESP) calculation[4] was carried out to parameterize the molecule in the AMBER gaff forcefield.[5]

Molecular Dynamics of Bowls $\mathbf{1}^{6+}$ and $\mathbf{2}^{6+}$: In order to investigate the conformational flexibility of bowl-shaped molecules $\mathbf{1}^{6+}$ and $\mathbf{2}^{6+}$ we ran unconstrained, unbiased molecular dynamics simulations. For each bowl, 200 ns of molecular dynamics were run saving frames for analysis every 4 ps. To analyze these trajectories the following pairwise distances were taken and found to clearly differentiate two conformations of the methylene bridge between the phenyl and pyridine rings of $\mathbf{1}^{6+}$ and $\mathbf{2}^{6+}$ (Figure S35). Using this distance metric, one is able to assign for each "arm," consisting of two pyridine rings linked via the phenyl ring and methylene units, i.e., a state of cis_{in}, cis_{out}, or trans. These states correspond to both pyridine Hs pointing in, both out, or one in and one out, respectively (see QM section for optimized structures of the conformers). After assignment of all of the single arms, the conformations were analyzed and the bowl was clustered into 10 states which reflect all the possible combinations of cis_{in}, cis_{out} and trans. It should be noted that we intentionally disregarded the difference between two anti-symmetric trans orientations and assigned both to the same state. If these states were included, there would be multiple additional states describing the conformations of all three arms. After assigning all of the conformations of the three arms to the 10 distinct states, a pie chart was made showing the population of the different states

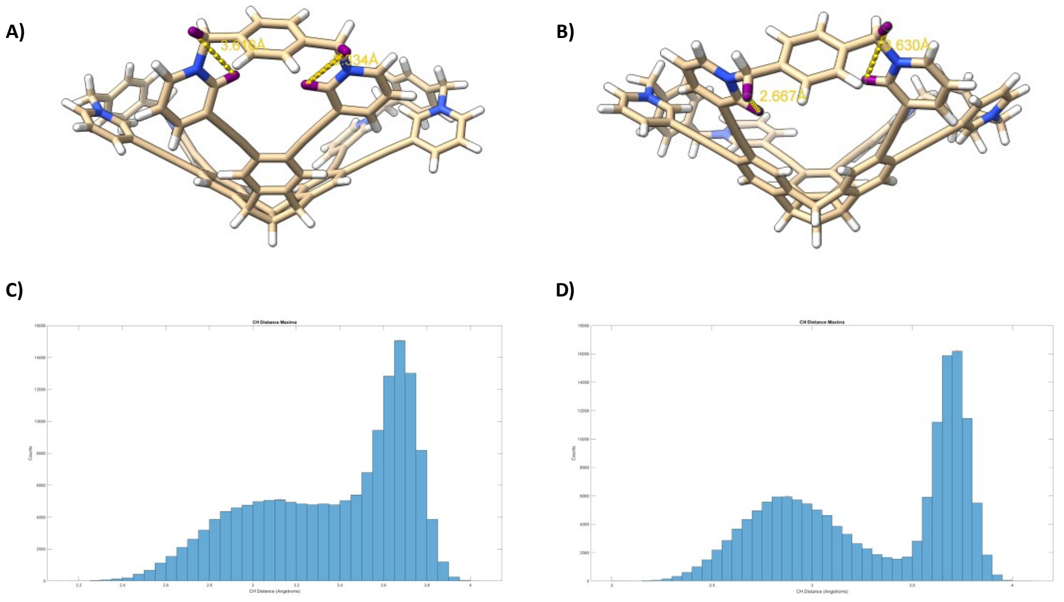

Figure S35. Representative structures for the pairwise distances used for clustering in (a) bowl $\mathbf{1}^{6+}$ and (b) bowl $\mathbf{2}^{6+}$. Histograms showing the distribution of CH-HC distances used for clustering (c) bowl $\mathbf{1}^{6+}$ and (d) bowl $\mathbf{2}^{6+}$.

in the simulation. This was compared to a statistical distribution which was calculated by simply considering all possible combinations of single arm orientations which would be assigned to the 10 different states (Figure S36).

The last piece of analysis done with the bowls was the flexibility of the phenyl ring bridging the pyridines. Specifically, this was prompted due to an observation that in the case of bowl **1**$^{6+}$, there were two distinct H^1 NMR resonances for the phenyl ring at higher and lower temperatures. To investigate the phenyl ring's rotation, the dihedral angle around the methylene-phenyl bond was measured and plotted in a histogram

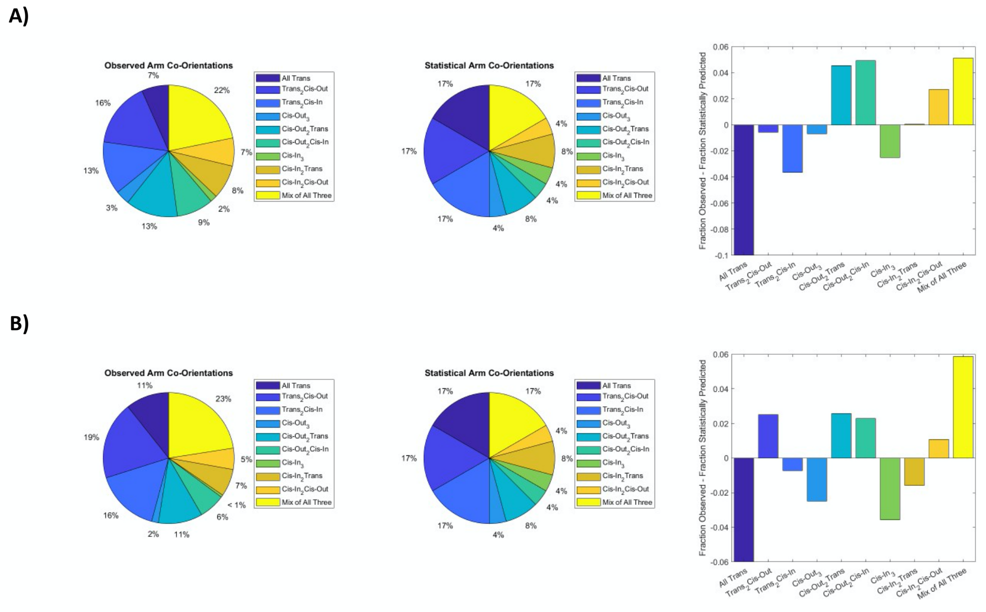

Figure S36. From left to right, a pie chart giving the populations of the state assignments for the co-conformations of the bowls' three arms, a pie chart showing the statistical populations expected based on equal energy conformations and the combinations of single arm orientations contributing to a state, and the difference between the observed and statistical populations: (a) bowl **1**$^{6+}$ and (b) bowl **2**$^{6+}$.

(Figure S37). *For both bowls, during the relatively short 200 ns simulation, there was no observed rotation about the methylene-phenyl dihedral*. However, it was noted that for bowl **1**$^{6+}$, the rotation was somewhat hindered when compared to bowl **2**$^{6+}$. Comparison of the standard deviations of the dihedral angle shows this difference (Table S1).

A) B)

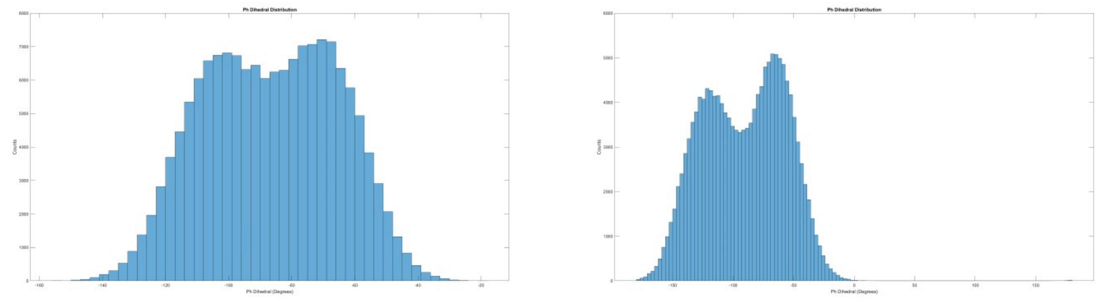

Figure S37. Histograms showing the distribution of the phenyl-methylene dihedral angle for (a) bowl $\mathbf{1}^{6+}$ and (b) bowl $\mathbf{2}^{6+}$.

Table S1. Tables describing the standard deviation of the dihedral angle around the Phenyl-Methylene bond.

Bowl $\mathbf{1}^{6+}$ Phenyl-Methylene Dihedrals			
	Ring 1	Ring 2	Ring 3
Average	-86.7547	-86.6646	-86.5153
STD	21.21173	21.34417	21.31354
		Cumulative Avg	-86.6449
		Cumulative STD	21.28981

Bowl $\mathbf{2}^{6+}$ Phenyl-Methylene Dihedrals			
	Ring 1	Ring 2	Ring 3
Average	-91.2115	-91.5093	-90.3982
STD	34.01659	33.74055	33.94829
		Cumulative Avg	-91.0397
		Cumulative STD	33.90181

DFT Optimization of Observed Bowl States: Following molecular dynamics studies with $\mathbf{1}^{6+}$ and $\mathbf{2}^{6+}$, a series of *ab initio* calculations were carried out in order to obtain optimized geometries for each of the observed 10 conformations. In addition, these quantum calculations were performed to give a source of comparison to the distribution of states found in the MD simulations. For bowl $\mathbf{2}^{6+}$, optimization and frequency calculations ran smoothly for 9/10 conformers and the resulting energies are summarized in Table S2. For bowl $\mathbf{1}^{6+}$, vibrational frequency calculations were noted to give imaginary vibrational frequencies during initial calculations. In an attempt to remedy this, we attempted to do optimization and frequency calculations using the Int=SuperFineGrid keyword in Gaussian, instead of the Int=Ultrafine that is recommended for most DFT calculations. Changing this grid did result in some structures without imaginary frequencies, but only for 3/10 structures (Table S3). Finally, despite the presence of imaginary vibrational frequencies, the reported free energies were taken, and the structures were weighted via a Boltzmann distribution to give their expected populations at 298.15 K. These weights are reported in Tables S4 and S5 for bowls $\mathbf{2}^{6+}$ and $\mathbf{1}^{6+}$, respectively. It was seen in the quantum mechanics calculations that for both bowls one state, the $trans_3$, was one of the most populated. This observation corroborates evidence provided in the crystal structure of bowl $\mathbf{1}^{6+}$ which shows the bowl in the $trans_3$ state.

Table S2. Reported energies after optimization of the 10 conformational states for bowl $\mathbf{2}^{6+}$.

		Energies for Bowl $\mathbf{2}^{6+}$ Calculations							
# imaginary frequencies	Conformer	EE	ZPE	H	G	Relative EE(kcal/mol)	Relative ZPE (kcal/mol)	Relative H (kcal/mol)	Relative G (kcal/mol)
0	Trans3	-3715.6757	-3714.4552	-3714.3823	-3714.5693	2.5301	2.2496	2.3475	0.9036
0	Trans2Cis-Out	-3715.6772	-3714.4562	-3714.3834	-3714.5686	1.6133	1.6441	1.6415	1.3297
0	Trans2Cis-In	-3715.6736	-3714.4528	-3714.3799	-3714.5662	3.8353	3.7725	3.8096	2.8545
0	Cis-Out3	-3715.6797	-3714.4588	-3714.3860	-3714.5708	0.0000	0.0000	0.0000	0.0000
0	Cis-Out2Trans	-3715.6785	-3714.4575	-3714.3847	-3714.5694	0.7762	0.8339	0.8107	0.8227
0	Cis-Out2Cis-In	-3715.6765	-3714.4556	-3714.3827	-3714.5692	2.0482	2.0243	2.0707	0.9488
2	Cis-In3	-3715.6696	-3714.4487	-3714.3776	-3714.5576	6.3603	6.3710	5.2534	8.2422
0	Cis-In2Trans	-3715.6717	-3714.4504	-3714.3777	-3714.5632	5.0256	5.2541	5.2271	4.7207
0	Cis-In2Cis-Out	-3715.6733	-3714.4517	-3714.3790	-3714.5642	4.0687	4.4954	4.4076	4.1446
1	Mixture	-3715.6752	-3714.4541	-3714.3823	-3714.5635	2.8357	2.9398	2.3054	4.5450

Table S3. Reported energies after optimization of the 10 conformational states for bowl $\mathbf{1}^{6+}$.

		Energies for Bowl $\mathbf{1}^{6+}$ Calculations							
# imaginary frequencies	Conformer	EE	ZPE	H	G	Relative EE(kcal/mol)	Relative ZPE (kcal/mol)	Relative H (kcal/mol)	Relative G (kcal/mol)
0	Trans3	-3715.670	-3714.450	-3714.377	-3714.567	1.0285	1.9572	3.9369	0.5102
4	Trans2Cis-Out	-3715.672	-3714.452	-3714.383	-3714.561	0.1669	0.3508	0.2949	3.7324
2	Trans2Cis-In	-3715.672	-3714.452	-3714.381	-3714.562	0.0000	0.5641	1.4564	3.1293
3	Cis-Out3	-3715.672	-3714.452	-3714.382	-3714.562	0.0998	0.3495	0.8471	3.3358
0	Cis-Out2Trans	-3715.672	-3714.452	-3714.379	-3714.567	0.0056	0.8045	2.8206	0.0000
1	Cis-Out2Cis-In	-3715.672	-3714.452	-3714.380	-3714.564	0.0515	0.7856	2.2182	2.1962
4	Cis-In3	-3715.669	-3714.448	-3714.376	-3714.564	2.0431	2.5175	3.9733	2.3732
1	Cis-In2Trans	-3715.672	-3714.452	-3714.380	-3714.566	0.0000	0.6306	2.1279	0.6539
0	Cis-In2Cis-Out	-3715.672	-3714.451	-3714.378	-3714.566	0.0301	0.9005	2.8840	0.8088
3	Mixture	-3715.672	-3714.452	-3714.382	-3714.561	0.0634	0.4869	0.8804	4.0336

NICS Calculations: A NICS calculation was carried out by calculating magnetic shielding values for a box of ghost atoms with lengths 20 Å x 20 Å x 13 Å centered on the bottom of the host and a 0.1 Å distance between ghost atoms. For each host, $\mathbf{1}^{6+}$ and $\mathbf{2}^{6+}$, the trans$_3$ conformation was chosen for the calculation since both DFT calculation's Boltzmann weights and the crystal structure of bowl $\mathbf{1}^{6+}$ had shown that conformation as a major conformer. To generate the ghost atom coordinates, a MATLAB[6] script was written which can determine a Z axis via the selection of 3 co-planar atoms at the bottom of the host. For both hosts, the optimized trans$_3$ structure was used, and in both cases this conformer had no imaginary vibrational frequencies after the analytical frequency calculation. Magnetic shielding values were computed at each of the ghost atom coordinates with B3LYP/6-31+G(d) level of theory with PCM water solvation using the GIAO method.[7-8] The resulting data were analyzed and plotted using MATLAB resulting in shielding maps of the two hosts (Figure S38 and S39).

A) B)

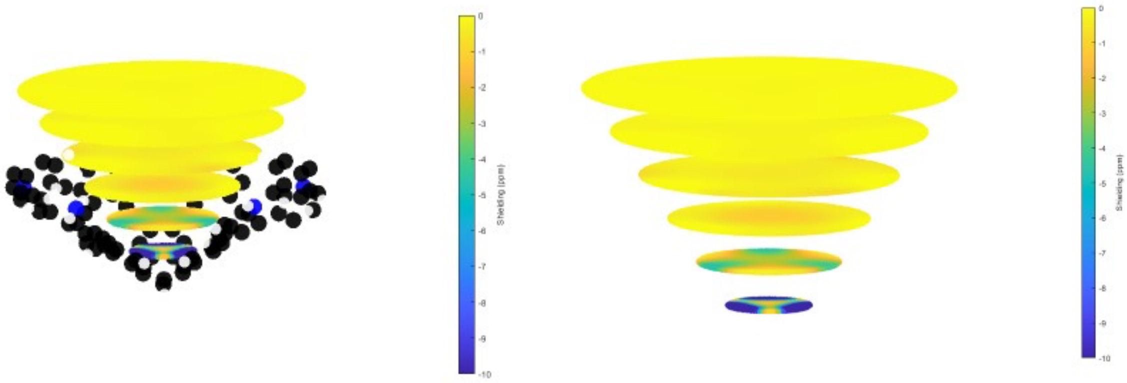

Figure S38. Depictions of the shielding environment for bowl $\mathbf{1}^{6+}$ (a) with host atoms shown (b) without host atoms shown.

Guest Δ*δ* Fitting - Concept: In NMR titrations for host-guest complexes, generally one observes a steady shift in the chemical shift of resonances corresponding to the host or guest which are then fit to complexation equilibria models to obtain binding constants. Intrinsic to this process is the generation of a parameter, $\Delta\delta$, which describes the change in chemical shift between the bound and free guest:

$$\Delta\delta = \delta_{bound} - \delta_{free}$$

Contributing to the observed chemical shift can be many equilibria. For instance, the δ_{free} state is the weighted average of the chemical shifts of all exchanging conformations of the molecule in solution. The bound state's chemical shift, δ_{bound}, is likewise comprised of the weighted average of the chemical shifts of all exchanging conformations of the host-guest complex. To estimate this experimentally observable parameter, the combination of two calculations was proposed: a Monte-Carlo Molecular Mechanics (MCMM) search with the host frozen (docking) and the previous NICS calculation. In this workflow, the

NICS calculation provides a map which estimates the "shielding" effect which is typically considered the main contribution to the change in chemical shift upon binding, $\Delta\delta$. With this map, one can take docked poses of the guest molecule in the host and assign a shielding value to each proton based on the closest surrounding points in the NICS map (< 0.1 Å). After each proton in each pose has been assigned an estimate of the $\Delta\delta$ value, one can then compare the assigned estimates to the observed $\Delta\delta$ values. Using a RMSE criteria, the pose which best reflected the observed $\Delta\delta$ was selected as a representative conformation for the host-guest complex.

Guest $\Delta\delta$ Fitting - Procedure: After generation of the NICS maps for the two hosts, a MCMM search was carried out for 1:1 host-to-guest complexes using MacroModel. The MCMM search was prepared such that the hosts' coordinates were frozen while the guest was allowed to freely move, rotate, and go through dihedral changes within the pocket of the host. This was done by performing manual set-up for the MCMM search within the MacroModel GUI as explained in MacroModel's manual.[9] From this calculation, essentially docking, many poses were generated for each host. Importantly, the hosts' coordinates were fixed in the same position as for the previous NICS calculation. This allowed the direct comparison of the coordinates of the hydrogens on the guest molecule to the coordinates of ghost atoms in the NICS map. By using a distance criterion, ghost atom coordinates within 0.1 Å of each proton were found and the average of all points within that distance criteria was assigned to the proton as the $\Delta\delta_{comp}$ value. For each pose a list of the $\Delta\delta_{comp}$ values were compared to the list of $\Delta\delta_{exp}$ values using a RMSE method:

$$RMSE = \sqrt{\sum_{i=1}^{n}\left(\Delta\delta_{comp,i} - \Delta\delta_{exp,i}\right)^2}$$

In order to carry out this calculation, a for loop was used which iterated over the entire list of poses and calculated the RMSE, as shown above, for each pose. Within this for loop, an if statement was used that compares the current RMSE to the lowest previously calculated value. If the current RMSE is the lowest calculated, that is updated in a variable which tracks the pose which had the lowest RMSE. The pose with the lowest RMSE was taken as the representative structure for the host guest complex (Figure S39 and S40). Future studies may require expanding the approach to an ensemble-weighted method in which all poses contribute to an averaged $\langle\Delta\delta_{comp}\rangle$ which may be compared to the observed $\Delta\delta_{exp}$ values. The MATLAB script used to do this analysis may be provided upon e-mail request to the corresponding author.

A) B)

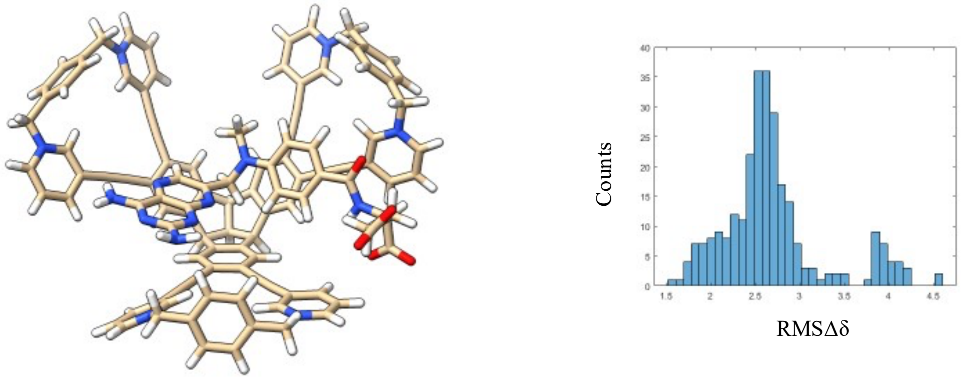

RMSΔδ = 1.5621

Figure S39. (a) Pose with the lowest RMSE for bowl **1**$^{6+}$. (b) Histogram showing the distribution of RMSE for all the MCMM poses.

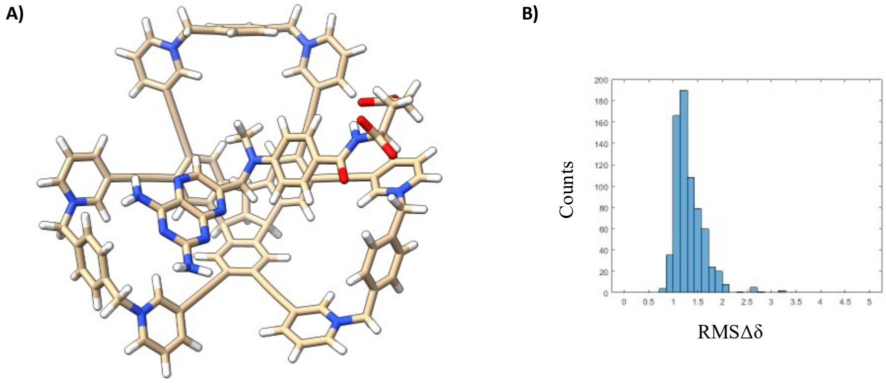

RMSΔδ = 0.7895

Figure S40. (a) Pose with the lowest RMSE for bowl **2**$^{6+}$. (b) Histogram showing the distribution of RMSE for all the MCMM poses.

Optimized Coordinates from DFT Calculations:

Bowl **1**$^{6+}$ - Trans$_3$

E(RB3LYP) = -3715.670251

Number of Imaginary Frequencies = 0

Cartesian Coordinates:

C 19.03381000 14.30907700 2.01818000

C 20.59664100 10.90082300 1.35504400

C 21.84016900 11.98993600 3.12191700

C 19.98865300 13.14039700 2.06136300

C 21.04431500 13.12558000 2.98463900

C 21.60253000 10.85669700 2.32959700

C 19.78971000 12.03289600 1.22743500

C 17.19133500 15.68841400 4.99070400

C 17.95130200 15.59545400 3.83389900

C 22.38965500 9.59501700 2.55740600

C 20.52912500 8.87700700 4.07810300

C 22.25478300 7.36288200 3.56208200

H 20.41809800 10.03972100 0.71633000

H 22.63664800 11.97758800 3.86108600

H 21.22767100 13.98827700 3.61977400

H 18.98923300 12.04225200 0.49259000

H 17.00275000 16.66561400 5.41931700

H 20.12646700 9.86905000 3.93468100

H 18.33507300 14.23010900 1.18242900

H 19.55816100 15.26278400 1.94533200

H 18.37259500 16.45901900 3.33575100

H 23.17518800 7.20671200 3.01324200

H 22.58781400 9.07223000 1.61999400

H 23.34050700 9.80158200 3.05330300

N 18.20509400 14.38738800 3.27923100

N 21.67646300 8.58254200 3.43814100

C 15.55879200 -1.81311900 2.18854800

C 11.91213000 -1.21321600 1.26852500

C 12.00543400 -2.93023900 2.97060400

C 14.05817700 -1.97000200 2.10804700

C 13.39736800 -2.88461500 2.94218400

C 11.25084800 -2.07131200 2.15651100

C 13.30761900 -1.15807700 1.24942000

C 17.36992900 -1.26809100 5.43751600

C 16.99880700 -1.77865800 4.20210700

C 9.75159500 -2.04155500 2.27149200

C 10.06319700 -0.21125800 3.95966000

C 7.89533200 -0.98792700 3.47769700

H 11.34005500 -0.56125300 0.61363200

H 11.50359500 -3.62184000 3.64212500

H 13.96679900 -3.54454500 3.59107100

H 13.80811500 -0.46298300 0.58073700

H 18.19025900 -1.73144700 5.97282200

H 11.12009200 -0.30314500 3.75742800

H 15.93799800 -1.14526400 1.41218900

H 16.07674100 -2.76931100 2.10750500

H 17.49115800 -2.62642000 3.74343700

H 7.30169100 -1.68370500 2.89791800

H 9.28289800 -1.76607500 1.32461300

H 9.35603200 -3.00710900 2.59235300

N 15.97108200 -1.22705100 3.51688900

N 9.23672100 -1.03408400 3.28756900

C 3.29061400 9.23230600 2.13046000

C 13.34539100 5.40613400 8.16948300

C 13.90200200 8.84978500 8.11275100

C 10.63721500 7.62629400 8.16074500

C 15.60968200 0.38391900 5.25031600

C 16.97437900 13.27765500 5.00722400

C 5.20702800 8.03420900 5.15273400

C 5.48459200 12.24624400 1.36170300

C 3.93221800 12.85092500 3.11675800
C 3.83785500 10.63908800 2.12895700
C 3.33583000 11.59533700 3.02486900
C 5.03200500 13.17532900 2.30723900
C 14.11901300 2.31300900 6.26023600
C 16.13720400 11.02245500 6.09159400
C 7.57642600 8.46681700 6.23069300
C 11.94250900 5.40694600 8.16062600
C 14.61159200 7.63943600 8.12605800
C 11.33058300 8.84612200 8.14803200
C 11.93878100 3.37309200 6.83455200
C 16.35360900 8.63142000 6.75661700
C 9.55586800 9.85492100 6.83369400
C 11.24302400 4.40389300 7.49892400
C 15.82592800 7.52817300 7.45800000
C 10.79743100 9.95178500 7.49485400
C 12.63780900 7.30988100 9.52118200
C 11.37745500 6.56865300 8.96153600
C 13.90229200 6.58141900 8.95527900
C 12.62731400 8.76214100 8.93606400
C 14.05274900 4.38943100 7.53618300
C 14.40551000 9.95266200 7.43107600
C 9.40604200 7.51044600 7.52445600
C 14.82088900 1.44665700 5.77353000
C 16.51528400 12.05857300 5.57804200
C 6.48870300 8.26319200 5.72517500
C 16.67077200 -0.19443100 5.97869300
C 16.70268500 14.53262700 5.59105700
C 4.25635700 7.18023600 5.74991400
C 13.36420700 3.35511400 6.87232800
C 15.63014800 9.86053500 6.74039600
C 8.84709000 8.61757400 6.85592000
C 4.89497700 10.98307400 1.27822900
C 15.29587200 -0.16454200 4.00441100
C 17.72194900 13.25265900 3.82672700
C 4.85685900 8.67119900 3.95902200
C 3.01341000 7.01064600 5.14850300
C 2.71609700 7.69220000 3.97755800
C 5.74254500 14.48870800 2.48571700
C 11.18364300 2.38365300 6.14285100
C 17.59829000 8.47534700 6.08341800
C 9.04509800 11.00870400 6.17401100
C 8.17455400 0.78143700 5.07745900
C 20.48221000 6.66115700 5.02201100
C 9.01321100 14.45846100 5.24756500
C 7.24513600 13.29165600 4.10052900
C 7.34417200 -0.08548200 4.37165700
C 21.67320800 6.38775000 4.35467200
C 8.61883100 15.62551700 4.59820800
C 10.45858100 1.60110700 5.55650000
C 18.65927000 8.25202400 5.53002800
C 8.68775500 12.03627500 5.62799000
C 7.55161300 15.59276300 3.71665300
C 9.56774300 0.72698500 4.87530800
C 19.88524000 7.93019300 4.88615200
C 8.31843800 13.25795100 5.00119200
H 6.31830000 12.49356800 0.70987500
H 3.55450200 13.57497600 3.83391400
H 2.49736700 11.35163600 3.67216500
H 10.15766200 4.38899300 7.48782000
H 16.39288200 6.60256100 7.47479100
H 11.31561200 10.90570000 7.49217500
H 15.13772100 4.36124700 7.56027700
H 13.87750900 10.90118100 7.42935800
H 8.84762800 6.57979700 7.54582300
H 16.93175900 0.19886200 6.95523200
H 16.12266700 14.58814600 6.50572100
H 4.49673500 6.66815500 6.67519900
H 5.27621700 10.25791000 0.56444500
H 14.48854300 0.22218600 3.39718700

H 17.96208100 12.32617900 3.32343400

H 5.53951400 9.33376100 3.44471700

H 2.26158700 6.36466300 5.58628400

H 7.75843400 1.49654500 5.77861100

H 20.01018100 5.90612300 5.64092600

H 9.84883300 14.46655200 5.93877300

H 6.67224400 12.40595300 3.86780400

H 6.26921900 -0.06685800 4.50724100

H 22.15399700 5.42019100 4.43847200

H 9.13359100 16.56369100 4.76898800

H 3.70029800 8.63950200 1.30962200

H 2.20218300 9.21647700 2.06260100

H 1.75979200 7.61323400 3.47655900

H 7.20097600 16.46564100 3.18000900

H 6.18835200 14.83265500 1.55038000

H 5.06667300 15.26063600 2.85863300

N 3.63531300 8.50725100 3.41012700

N 6.88846500 14.43362400 3.48351000

H 12.63996500 7.32103700 10.61186300

H 10.73245800 6.18845900 9.76331900

H 12.63769000 9.52566500 9.72358400

H 14.56532900 6.22221900 9.75175000

Bowl **1**$^{6+}$ - Trans$_2$Cis-in

E(RB3LYP) = -3715.671939

Number of Imaginary Frequencies = 2

Cartesian Coordinates:

C 1.32851800 0.53560100 4.06972800

C -0.19501400 -1.47813400 4.03568900

C -1.17717500 0.84851600 4.06716800

C 1.18684800 3.80416500 1.98726000

C -0.22864100 3.97843400 1.98046700

C -1.04459400 3.03877400 2.64240600

C -0.47387200 1.94463600 3.28357600

C 0.91853800 1.77203700 3.28783700

C 1.74382800 2.69404800 2.65370700

C -3.88890600 -0.86399500 1.88404700

C -3.33195200 -2.17697900 1.86327100

C -2.12498900 -2.42692000 2.54744900

C -1.47905500 -1.39985200 3.22644100

C -2.02416100 -0.10698300 3.24282700

C -3.21828100 0.16073400 2.58243000

C 2.67662400 -2.92494400 1.86589400

C 1.44552700 -2.87230700 2.55062600

C 1.06919200 -1.71713400 3.22690100

C 1.91354400 -0.59668500 3.24174800

C 3.13257400 -0.62591600 2.57359400

C 3.53077600 -1.78260700 1.87272100

C -0.01614500 -0.04009700 4.62781300

C 2.88895400 5.47955800 0.86486900

C 2.06707800 4.73337900 1.36368300

C -1.45723000 6.01476200 0.83606300

C -0.85013100 5.09194000 1.34683300

C -3.51114800 9.25117800 -0.86993800

N -2.28970800 8.88121000 -1.32418600

C -1.63447900 7.83344400 -0.78629900

C -2.18365100 7.09869200 0.27084700

C -3.45618700 7.47453300 0.74653200

C -4.11420800 8.55443900 0.16514100

C 5.71528200 8.09661300 -0.79581300

C 6.12004200 7.23778600 0.21320200

C 5.20266800 6.35735600 0.77991200

C 3.87170200 6.34867300 0.31695400

C 3.52794100 7.23331900 -0.71232100

N 4.43468200 8.07944000 -1.23832600

C -1.70284700 9.64657300 -2.49331300

C 4.07245400 9.01023000 -2.38063500

C -0.19926900 9.57188700 -2.54323400

C 0.43441800 8.71645600 -3.45212400

C 1.82267200 8.56337200 -3.42522800

C 2.59221100 9.26349000 -2.48856000

C 1.96233700 10.16979100 -1.62029200

C 0.57837900 10.32285800 -1.64752400

H -2.11852100 3.19662400 2.64930000

H 2.82377300 2.58503100 2.67023100

H 2.02478800 0.81147700 4.87115300

H -1.72248400 -3.43499000 2.53917100

H -3.66255900 1.15091500 2.60457000

H -1.78324800 1.29047700 4.86758100

H 0.81222000 -3.75395300 2.54945000

H 3.80061900 0.22965400 2.58626700

H -0.29040000 -2.24034800 4.81867000

H -0.01924800 -0.05586900 5.71839500

H -3.97227400 10.10263600 -1.35430800

H -3.91056000 6.92596000 1.56428500

H -5.09052100 8.87003700 0.51383700

H 6.38241300 8.80554700 -1.26981300

H 7.14875200 7.27091500 0.55230500

H 5.50190800 5.68414400 1.57573500

H 2.52693500 7.27068300 -1.11743800

H 4.62336100 9.93266600 -2.19109100

H 4.46790600 8.54461200 -3.28627500

H -0.15530100 8.14750900 -4.16577100

H 2.30065000 7.87637900 -4.11827700

H 2.55257800 10.74111700 -0.90856000

H 0.09982000 11.01216700 -0.95677500

H -2.05083500 10.67368100 -2.37550500

H -2.15582900 9.22405300 -3.39319800

H -0.66696500 7.59308600 -1.20380000

C -5.11624100 -0.55014700 1.23494000

C -6.15701100 -0.19448200 0.71373500

C -7.35752300 0.25647800 0.10040900

C -7.64505000 1.62761800 -0.05793300

C -8.29240500 -0.66959700 -0.37672300

C -8.82743600 2.01293800 -0.68257100

H -6.94233600 2.37135900 0.30146300

H -8.13755600 -1.73494900 -0.28104500

N -9.42410800 -0.26829400 -0.98906000

C -9.70560200 1.04715400 -1.14813600

H -9.07280200 3.05914500 -0.82237500

C -10.44215400 -1.27825700 -1.47834700

H -10.63445300 1.28762100 -1.64968200

C -9.82679900 -2.61567100 -1.79848900

H -11.19514000 -1.36145900 -0.69132000

H -10.90365800 -0.82596200 -2.35754200

C -9.01960000 -2.77187900 -2.93409900

C -10.01581700 -3.70344700 -0.93495200

C -8.35094500 -3.97484500 -3.15964500

H -8.88476600 -1.94492300 -3.62640300

C -9.36225600 -4.91282900 -1.17098400

H -10.65921300 -3.60072600 -0.06529800

C -8.49492900 -5.04411400 -2.26427200

H -7.70053000 -4.07484200 -4.02443400

H -9.50391000 -5.74277200 -0.48356600

C -7.68651700 -6.30161500 -2.44705100

H -8.29211600 -7.19524900 -2.28822900

H -7.24196300 -6.35169100 -3.44346300

N -6.53772000 -6.41134800 -1.46364500

C -6.01036300 -5.31379700 -0.88678300

C -6.03437300 -7.64416800 -1.21380000

C -4.93498600 -5.40756000 0.00457300

H -6.46053600 -4.36335600 -1.13526700

C -4.96100500 -7.80140400 -0.35160400

H -6.51305400 -8.47471700 -1.71711100

C -4.40356400 -6.68648500 0.26751700

C -4.41674200 -4.23696000 0.62207900

H -4.57652300 -8.79730000 -0.16539100

H -3.57013500 -6.79401300 0.95304000

C -3.95750200 -3.25738100 1.17972400

C 3.03149400 -4.13372200 1.20365800

C 3.25448600 -5.20571700 0.67243300

C 3.47779300 -6.46247300 0.04727700

C 2.44266000 -7.40317200 -0.12823600

C 4.75398100 -6.79480100 -0.42193900

C 2.71498300 -8.61244600 -0.76092200

H 1.44247900 -7.17734600 0.22504000

H 5.59239000 -6.12179600 -0.31211900

N 4.98695900 -7.96865500 -1.04164600

C 3.99678400 -8.87649800 -1.21662600

H 1.93872000 -9.35298300 -0.91354900

C 6.37702900 -8.33302300 -1.52292100

H 4.26500400 -9.79547200 -1.72226000

C 7.23528700 -7.12612000 -1.79933100

H 6.81345300 -8.96572400 -0.74683300

H 6.22693500 -8.93421300 -2.42111400

C 6.99381200 -6.32654000 -2.92553400

C 8.25390500 -6.76588300 -0.90653900

C 7.71091100 -5.14552100 -3.11434500

H 6.22506900 -6.60800100 -3.64053500

C 8.98306100 -5.59322500 -1.10484700

H 8.46618100 -7.39163500 -0.04390100

C 8.69106900 -4.75592400 -2.19031900

H 7.49422000 -4.51634800 -3.97345900

H 9.75796800 -5.31616100 -0.39489200

C 9.38626600 -3.42860500 -2.33556600

H 10.45117200 -3.50710800 -2.11073300

H 9.26856300 -3.02233000 -3.34245800

N 8.85162700 -2.36938500 -1.38994500

C 7.63886000 -2.48981800 -0.81623800

C 9.62511600 -1.27845700 -1.17095200

C 7.13854800 -1.49953200 0.03845800

H 7.07623800 -3.38493900 -1.03937800

C 9.18123700 -0.25896900 -0.34468200

H 10.58595400 -1.25782500 -1.66977500

C 7.93628900 -0.36078000 0.26962800

C 5.86549700 -1.66048900 0.64979900

H 9.81784400 0.60287000 -0.18259700

H 7.57849000 0.42453000 0.92642400

C 4.78439700 -1.76700000 1.19884500

Bowl **1**$^{6+}$ - Trans$_2$Cis-out

E(RB3LYP) = -3715.671673

Number of Imaginary Frequencies = 4

Cartesian Coordinates:

C 4.38483400 8.96328400 -2.39974200

C 6.99813500 6.25925900 -2.97943600

C 7.59198000 7.49028900 -0.98199700

C 5.62801400 8.12556900 -2.25419800

C 6.50226400 8.33780300 -1.17884400

C 7.83160400 6.42771000 -1.86507700

C 5.89938000 7.09783300 -3.16803800

C 1.29899600 9.20156100 -0.28241500

C 2.33942900 9.50391400 -1.14611500

C 8.94585300 5.45247900 -1.59481200

C 7.25485900 4.05266200 -0.39680700

C 9.32637900 3.10432300 -0.98520800

H 7.18908600 5.45599700 -3.68622600

H 8.24726400 7.64601500 -0.12920900

H 6.31851800 9.14833400 -0.47858800

H 5.24236600 6.93984500 -4.01902100

H 0.54402700 9.95304000 -0.08334600

H 6.62663200 4.93134000 -0.41632900

H 3.94384700 8.85820400 -3.39348500

H 4.58921100 10.01943200 -2.21715600

H 2.43761500 10.46308600 -1.63858100
H 10.28402000 3.26832200 -1.46335300
H 9.45486000 5.16243000 -2.51560000
H 9.68155000 5.86634700 -0.90165600
N 3.29842100 8.58457700 -1.41236000
N 8.46861200 4.15346500 -0.97150800
C 5.65405200 -8.17923000 -2.39096500
C 2.02416300 -9.14580700 -2.99035900
C 2.80374000 -10.27772500 -0.99840700
C 4.31963600 -8.86480800 -2.25705600
C 4.07667900 -9.74095600 -1.18991400
C 1.76111100 -9.96087700 -1.88049300
C 3.29248200 -8.59454800 -3.17190900
C 7.35695500 -5.68264300 -0.16969100
C 7.11867200 -6.71457200 -1.06336500
C 0.36338100 -10.45223900 -1.61116600
C -0.05417300 -8.28038500 -0.45335500
C -1.88763600 -9.55819400 -1.19140400
H 1.23197000 -8.91471300 -3.69764400
H 2.61736600 -10.93023200 -0.14968700
H 4.87163700 -9.98119700 -0.48885200
H 3.47761700 -7.93738200 -4.01738600
H 8.38045300 -5.41611100 0.06640700
H 1.02139000 -8.20059500 -0.38644300
H 5.77097500 -7.71750900 -3.37385000
H 6.48072200 -8.87259700 -2.22926400
H 7.91171500 -7.27319900 -1.54424800
H -2.20625700 -10.46135200 -1.69664300
H -0.11835300 -10.80914800 -2.52279700
H 0.35553100 -11.25650700 -0.87201000
N 5.84957700 -7.07123700 -1.37550200
N -0.55124700 -9.37087400 -1.06896800
C 1.21699800 -1.57234100 3.20763300
C 0.73600500 1.87981800 3.18957400
C -2.01883100 -0.26905700 3.16638800
C 4.96752500 -5.39702900 0.09085400
C 2.24988100 6.99973100 0.04350700
C 2.89040000 -4.21800300 1.21118300
C 2.23987100 4.62475600 1.19202900
C -0.11975000 -1.99852100 3.19411500
C 1.77361500 0.93552100 3.21390400
C -1.71473100 1.10064200 3.15739600
C 0.49805200 -3.93090800 1.85970500
C 3.17019100 2.41825300 1.89234100
C -3.67292600 1.53863700 1.79006500
C -0.47814200 -3.16750700 2.53184700
C 2.98096100 1.20070100 2.57748300
C -2.53128000 1.99788800 2.47797000
C -0.03998700 0.01885100 4.56442800
C -1.01539300 -1.06163200 3.98797100
C 1.39181700 -0.29731100 4.01592500
C -0.47281000 1.40490300 3.97856400
C 2.19727300 -2.31097100 2.55440200
C 0.90177500 3.08941600 2.52417000
C -3.14198400 -0.74398000 2.49859500
C 3.83622600 -4.76716100 0.67714000
C 2.25569100 5.71275400 0.64668000
C 6.28517300 -5.01718600 0.41801700
C 1.24575900 7.94905600 0.32228900
C 1.85574900 -3.49373400 1.86755100
C 2.11306700 3.37534900 1.86182300
C -3.98342300 0.14659500 1.80111100
C 4.79318000 -6.43560700 -0.83139800
C 3.26592500 7.36085700 -0.84942000
C 0.08948300 -5.12844500 1.20785300
C 4.42782200 2.65414900 1.26917500
C -2.29052800 -7.48013800 -0.05594500
C 7.71456600 1.76076500 0.18348400
C -2.77568800 -8.62291600 -0.68524800
C 8.96740900 1.89743900 -0.40787600

C -0.34870700 -6.13517000 0.68250200

C 5.53306100 2.76835700 0.77231900

C -0.89893000 -7.29144300 0.06537800

C 6.82797700 2.85613200 0.19295300

H -1.50448000 -3.52105600 2.53169000

H 3.79981200 0.48869500 2.60786400

H -2.31624600 3.06190200 2.47567800

H 3.23694200 -1.99938100 2.56774800

H 0.11331100 3.83539000 2.51010900

H -3.39909100 -1.79844300 2.51182400

H 6.45510700 -4.21579100 1.12867500

H 0.44398100 7.70063500 1.00900200

H 3.81014800 -6.77024200 -1.13122900

H 4.06129900 6.67798100 -1.11207600

H -2.97259600 -6.73377200 0.33618700

H 7.41518600 0.82012000 0.63263000

H -3.84012700 -8.79313900 -0.79602300

H 9.66932800 1.07223300 -0.43193700

H -0.05504200 0.02663000 5.65499100

H -1.53288700 -1.61752000 4.77937700

H -0.71370300 2.12951700 4.76606500

H 2.11744100 -0.46111700 4.82202500

C -5.12385200 -0.38415000 1.13491200

C -6.06909200 -0.92995400 0.59622200

C -7.15629700 -1.60670400 -0.02069200

C -7.21522700 -3.01247200 -0.09189400

C -8.21255000 -0.87486600 -0.57878000

C -8.30485900 -3.62287000 -0.70774800

H -6.41419400 -3.60874400 0.33105100

N -9.25300200 -1.49111300 -1.16981000

H -8.22901000 0.20467700 -0.55262800

C -9.31634700 -2.84326700 -1.24318600

H -8.37728900 -4.70179700 -0.77838000

C -10.41707700 -0.71356600 -1.76062000

H -10.18735600 -3.26038600 -1.73313700

H -10.54218900 -1.10097200 -2.77333400

H -11.28945100 -0.99238100 -1.16599500

C -10.20746500 0.77611600 -1.76472100

C -10.65334900 1.55509700 -0.68915000

C -9.53307100 1.39460400 -2.82894200

C -10.37417800 2.92224900 -0.64678600

H -11.19823700 1.09161500 0.12895300

C -9.25922100 2.76028800 -2.78940400

H -9.20731000 0.80475900 -3.68172200

C -9.65112700 3.52831200 -1.68214200

H -10.70370900 3.51235900 0.20412200

H -8.72213300 3.22534600 -3.61190600

C -9.26214700 4.97989100 -1.58666000

H -9.36193800 5.48536000 -2.54838600

H -9.86768600 5.50900900 -0.84745700

N -7.81807400 5.18296600 -1.17031600

C -7.13766100 4.21921000 -0.52042600

C -7.24953100 6.38373400 -1.43654200

H -7.65744300 3.28995700 -0.33697800

C -5.81382200 4.41598300 -0.10938100

H -7.86342400 7.10488700 -1.96131400

C -5.94705500 6.64492100 -1.04303800

C -5.21643400 5.66377000 -0.37861900

C -5.11226000 3.37165900 0.55264100

H -5.51296400 7.61220300 -1.26741300

H -4.19283300 5.84960300 -0.07230100

C -4.48300400 2.49172400 1.11036900

Bowl $\mathbf{1}^{6+}$ - Cis-in$_3$

E(RB3LYP) = -3715.67169

Number of Imaginary Frequencies = 1

Cartesian Coordinates:

C -8.56225200 3.31997200 -2.76421000
C -5.53503800 5.40561000 -3.75793300
C -7.00023500 6.76821700 -2.39837600
C -7.64351700 4.50086200 -2.97086100
C -7.92451700 5.72907800 -2.35023500
C -5.77806200 6.59889400 -3.06958200
C -6.46381300 4.36115000 -3.70910800
C -9.54812500 2.07306400 0.64740400
C -9.61212600 2.51726300 -0.66542600
C -4.71069800 7.66213500 -2.98135300
C -3.83165000 6.57565200 -0.93861600
C -3.80655100 8.92594300 -1.05427300
H -4.60541300 5.27105800 -4.30449500
H -7.21718900 7.70298200 -1.88791900
H -8.85406500 5.86400300 -1.80351900
H -6.24559900 3.42548500 -4.21708900
H -10.46906700 1.84220100 1.16977100
H -4.08941800 5.65208300 -1.43844200
H -8.30143500 2.48262700 -3.41487700
H -9.60832000 3.57908500 -2.93134400
H -10.54597900 2.65040700 -1.19621300
H -4.04832300 9.80014000 -1.64498600
H -3.89129200 7.47375100 -3.67860900
H -5.10969500 8.65857400 -3.17373900
N -8.47846100 2.81565200 -1.34108400
N -4.09960600 7.72180500 -1.59834300
C -1.89483100 0.68891100 3.58535300
C 0.35207700 -1.98233300 3.58889800
C 1.54470800 1.29714900 3.58642200
C -7.13707300 2.26318200 0.56452300
C -4.72849600 2.10347700 1.63397900
C -0.98611300 1.75756000 3.58127300
C -1.02786800 -1.73067300 3.58746900
C 2.01822900 -0.02315900 3.59153100
C -2.52323300 3.07404000 2.24424200
C -1.40166400 -3.72088300 2.25233000
C 3.93552700 0.64737000 2.26537800
C -1.29509600 2.94232500 2.92085700
C -1.90063400 -2.59238000 2.93091200
C 3.20577500 -0.34799900 2.94343900
C -0.00049300 0.00358500 4.96195400
C 0.26112800 1.43648600 4.38816100
C -1.37302500 -0.48841800 4.39215800
C 1.11156700 -0.94132300 4.39503000
C -3.11902300 0.80208400 2.93386400
C 0.86515700 -3.09838700 2.93536200
C 2.25621200 2.29837400 2.93303400
C -5.83988700 2.16609300 1.14362400
C -8.31330900 1.95056600 1.27668600
C -3.44821800 1.98876700 2.25109600
C 0.00111200 -3.97700100 2.25396700
C 3.45368200 1.98943400 2.25980500
C -7.26636500 2.67496500 -0.76416000
C -2.81838400 4.29666300 1.57337900
C -2.95847700 7.83766800 0.91458700
C -3.22834100 9.00336700 0.20429700
C -3.02218300 5.35586600 1.01058900
C -3.26793000 6.58752000 0.34078700
H -0.60940800 3.78402300 2.92479900
H -2.97245600 -2.42059600 2.93978000
H 3.59612100 -1.36079300 2.95918700
H -3.84290700 -0.00683600 2.95378200
H 1.92784200 -3.31976300 2.95086800
H 1.91405000 3.32852100 2.94235200
H -8.24911100 1.62306000 2.30853500
H -6.40539300 2.92405300 -1.36936900
H -2.51877900 7.88346600 1.90489900
H -3.00537700 9.97825200 0.62166900
H -0.00147700 0.00670200 6.05267100
H 0.39514400 2.18180000 5.18181300

H 1.68526200 -1.43026100 5.19190400
H -2.08419400 -0.74296400 5.18757100
C 0.54423800 -5.13128800 1.61710300
C 1.05019200 -6.10814200 1.09802600
C 1.62060700 -7.25803000 0.48209900
C 2.52695900 -8.10838300 1.14833600
C 1.28655300 -7.56431100 -0.83960800
C 3.04328700 -9.21572600 0.48246700
H 2.81068800 -7.89847500 2.17386300
N 1.78113100 -8.65972800 -1.45302100
H 0.60189700 -6.95223500 -1.41064100
C 2.65123500 -9.48116200 -0.82168000
H 3.73911300 -9.88891200 0.96914900
C 1.35569900 -8.96589300 -2.87097500
H 3.00685000 -10.33718100 -1.38057800
C -0.13415200 -8.78517700 -3.03901900
H 1.92243300 -8.29827100 -3.52376400
H 1.67056800 -9.99211700 -3.06304000
C -1.02720700 -9.67322600 -2.41729200
C -0.63958800 -7.68665000 -3.74177700
C -2.39514900 -9.41762800 -2.43039600
H -0.65060900 -10.54963400 -1.89624500
C -2.01420300 -7.43006900 -3.75571400
H 0.03818900 -7.00594700 -4.24983800
C -2.89547400 -8.26911200 -3.06582900
H -3.07209300 -10.09716100 -1.91905400
H -2.39048200 -6.55248200 -4.27481000
C -4.35358600 -7.90163200 -2.93148000
H -4.63046500 -7.08613900 -3.60303300
H -5.01067400 -8.75019900 -3.12450500
N -4.67578200 -7.44015400 -1.52767400
C -3.82499300 -6.60756800 -0.89212700
C -5.81785900 -7.85824500 -0.93460200
H -2.93572300 -6.31237500 -1.43204100
C -4.07223400 -6.17266000 0.41286500
H -6.45013100 -8.52336000 -1.50855900
C -6.13174000 -7.44208800 0.35106000
C -5.26081100 -6.60224000 1.03819100
C -3.13148100 -5.31993800 1.05684000
H -7.05028700 -7.79181300 0.80733100
H -5.48524300 -6.28145600 2.04966100
C -2.31483800 -4.59880700 1.59812500
C 4.18392200 3.03677600 1.62523500
C 4.77812500 3.96455200 1.10963300
C 5.49069000 5.03457600 0.49788300
C 5.76878700 6.24426000 1.16690300
C 5.93413900 4.89839900 -0.82009800
C 6.47591100 7.24486200 0.50755700
H 5.43632800 6.38479900 2.18961900
N 6.64129700 5.87434700 -1.42719100
H 5.75020600 3.99970100 -1.39276200
C 6.91292600 7.03812600 -0.79288800
H 6.70658700 8.18375600 0.99686700
C 7.13330000 5.66124900 -2.84113600
H 7.48111600 7.77437700 -1.34669100
C 7.71636300 4.27867200 -3.00916600
H 6.27970400 5.82575000 -3.50215700
H 7.87066700 6.44396100 -3.02178300
C 8.93082300 3.94396400 -2.38791600
C 7.01337500 3.29471000 -3.71190300
C 9.38736200 2.62929800 -2.40112600
H 9.50584000 4.70546700 -1.86743800
C 7.47200600 1.97376700 -3.72489200
H 6.08628800 3.54535400 -4.22064600
C 8.63832600 1.62521400 -3.03611600
H 10.31314300 2.37787100 -1.89018400
H 6.89688000 1.21156900 -4.24384300
C 9.04272400 0.17705700 -2.90183100
H 8.46956400 -0.46812600 -3.57135600
H 10.10493900 0.02762600 -3.09813800

N 8.80583700 -0.32826500 -1.49649900

C 7.65772400 -0.00853500 -0.86373800

C 9.74029000 -1.10346600 -0.89941200

H 6.95674000 0.61016200 -1.40716700

C 7.40337700 -0.43719300 0.44196900

H 10.63370200 -1.31855700 -1.47134600

C 9.53596500 -1.57982500 0.38740100

C 8.37085100 -1.24718700 1.07143100

C 6.19138500 -0.05138100 1.08154000

H 10.29900000 -2.19728000 0.84640300

H 8.20478000 -1.59949800 2.08360600

C 5.15590600 0.29503900 1.61793200

Bowl **1**$^{6+}$ - Cis-in$_2$Trans

E(RB3LYP) = -3715.67189

Number of Imaginary Frequencies = 1

Cartesian Coordinates:

C -1.33404100 0.45826000 4.11784800
C 1.15018400 0.91331400 4.08898100
C 0.30027700 -1.46536700 4.06444200
C -3.45224300 -2.00268900 1.99632400
C -2.53053300 -3.09067000 1.96918500
C -1.28959800 -2.96281300 2.62545500
C -0.96431600 -1.78192600 3.28365100
C -1.87210900 -0.71237300 3.31242300
C -3.10630600 -0.81996800 2.68098800
C 3.44286100 -1.98092000 1.84836800
C 3.92713700 -0.63946900 1.86945800
C 3.21053000 0.34308800 2.58259400
C 2.04006300 0.00750800 3.25378000
C 1.56436200 -1.31241500 3.23427700
C 2.25646500 -2.29889800 2.54028000
C 0.00618000 3.96949700 1.99126400
C 0.87926600 3.08645100 2.65798900
C 0.37793900 1.96350100 3.30733300
C -1.00133400 1.70688400 3.31801900
C -1.88202000 2.56908500 2.67428300
C -1.39535700 3.70559500 1.99750100
C 0.04752300 -0.03969500 4.66061300
C 1.10407600 6.07635600 0.84191700
C 0.55643300 5.11759600 1.35364600
C -3.19773000 5.25446500 0.84998700
C -2.33135500 4.56970700 1.36150400
C -5.84201100 -2.05926000 0.87854200
C -4.73111600 -2.07782200 1.37534200
C -3.01861000 -5.40848400 0.80555500
C -2.83387500 -4.32288300 1.32363200
C 2.95379900 9.42916300 -0.87198900
C 3.59830000 8.77465700 0.16546700
C 3.00858800 7.65723000 0.74946200
C 1.76172400 7.20237800 0.27492400
C 1.16789200 7.89913800 -0.78389700
N 1.75786200 8.98284500 -1.32574800
C -3.71357700 -9.17956100 -0.89602300
N -4.38308400 -8.08409200 -1.32857400
C -4.15104100 -6.86939100 -0.79328800
C -3.22199000 -6.69671900 0.23933700
C -2.51795700 -7.83077000 0.69223100
C -2.77104200 -9.07128600 0.11399700
C -9.69363800 -2.01529900 -0.78204100
C -9.38588300 -1.09078700 0.20257200
C -8.11399300 -1.08251100 0.76853100
C -7.15287700 -2.01505700 0.32999800
C -7.52285900 -2.91719700 -0.67523000
N -8.76270800 -2.90637300 -1.20141000
C -6.17507900 7.65778900 -0.86948000
N -4.89427900 7.71160300 -1.30816500
C -3.93879800 6.93284600 -0.76421900
C -4.23072100 6.04930700 0.28181400
C -5.56154800 5.98434800 0.74104400
C -6.52969300 6.79465100 0.15474900
C -5.36547900 -8.25746000 -2.47011600
C -9.15619000 -3.85534900 -2.31844800
C -6.41507500 -7.17776800 -2.50679300
C -6.31142600 -6.12481100 -3.42345900
C -7.22225300 -5.06648700 -3.38672000
C -8.24578100 -5.04857000 -2.43172200
C -8.38709700 -6.13528700 -1.55404900
C -7.47992700 -7.19152300 -1.59180700
C 1.12853500 9.70735500 -2.49937300
C -3.12492400 9.00323700 -2.55428100
C -2.29081100 8.35414700 -3.47218600
C -0.91767900 8.61060000 -3.48026000
C -0.36385400 9.51887400 -2.57050800
C -1.20923300 10.21712300 -1.69366300
C -2.57821000 9.96174200 -1.68575800
C -4.58370000 8.64053100 -2.46648700
H -0.60692100 -3.80673600 2.61577200
H -3.82687200 -0.00862100 2.71412200
H -2.03080300 0.70769700 4.92749800
H 3.59949200 1.35627500 2.60405800
H 1.90958500 -3.32745400 2.53055500
H 0.45644800 -2.21885100 4.84620100
H 1.94147300 3.31003300 2.66175300
H -2.95268500 2.39201200 2.68967800
H 1.73829300 1.39098200 4.88213300
H 0.06290500 -0.05687800 5.75108600
H 3.36159400 10.30574700 -1.35935700
H 4.55311300 9.15125800 0.51325900
H 3.49634400 7.13964200 1.56819100
H 0.21638300 7.59872500 -1.19936500
H -3.95686900 -10.11836500 -1.37731900
H -1.79085700 -7.73132300 1.49072400
H -2.24938300 -9.96144800 0.44544800
H -10.66572300 -2.07205300 -1.25569300
H -10.14685100 -0.38904900 0.52325400
H -7.85989100 -0.36951200 1.54513700
H -6.83109300 -3.65255600 -1.06011300
H -6.88317600 8.31483600 -1.35852600
H -5.82132300 5.30972000 1.54942200
H -7.55975800 6.77018600 0.49034100

H -10.18225500 -4.15389500 -2.09811300
H -9.15237200 -3.26014700 -3.23436700
H -5.50509200 -6.11465100 -4.15179500
H -7.11690900 -4.24246300 -4.08728700
H -9.19546800 -6.14626100 -0.82750100
H -7.58810500 -8.01795700 -0.89417500
H -2.70542200 7.62703000 -4.16511200
H -0.27621600 8.08134400 -4.17963300
H -0.79408700 10.94623800 -1.00276600
H -3.22020800 10.49405200 -0.98874800
H -4.92462400 8.12573400 -3.36767400
H -5.20718400 9.52190700 -2.30918300
H 1.62456600 9.32518600 -3.39456800
H 1.39529800 10.75748700 -2.37244700
H -5.80919400 -9.24382700 -2.32721600
H -4.76950900 -8.27048200 -3.38556600
H -2.94051300 7.02421800 -1.16780300
H -4.71828600 -6.04064600 -1.19249300
C 4.12716500 -3.02305200 1.16150800
C 4.64444500 -3.97215600 0.60215300
C 5.20742300 -5.09474000 -0.06384300
C 4.46988900 -6.27385500 -0.29396600
C 6.52973000 -5.04762500 -0.52066200
C 5.06714300 -7.33652300 -0.96569800
H 3.44304600 -6.34340600 0.04787100
H 7.15199300 -4.17671000 -0.37166500
N 7.07922100 -6.08734300 -1.17857000
C 6.37602800 -7.22271600 -1.40635500
H 4.52352700 -8.25345500 -1.16078100
C 8.52065400 -6.04041300 -1.64466900
H 6.88923800 -8.01261200 -1.94021300
C 9.02537900 -4.63514200 -1.84377400
H 9.10350400 -6.57366700 -0.89028800
H 8.54537200 -6.61115600 -2.57423000
C 8.60754600 -3.88365600 -2.95137600
C 9.88284800 -4.05133800 -0.90120400
C 8.98601400 -2.54761400 -3.07639500
H 7.96433100 -4.33248300 -3.70356300
C 10.27248200 -2.71817500 -1.03353500
H 10.23334500 -4.63200700 -0.05221700
C 9.79801200 -1.94697100 -2.10345600
H 8.63421000 -1.96578600 -3.92409000
H 10.92426600 -2.27228100 -0.28680000
C 10.12001200 -0.47917400 -2.18549700
H 11.14584900 -0.27698900 -1.87242800
H 9.97837200 -0.09424600 -3.19775100
N 9.24694600 0.37980500 -1.28795800
C 8.11455000 -0.09783100 -0.73763200
C 9.63746500 1.66169100 -1.08570700
C 7.30635000 0.70779400 0.07471300
H 7.86285200 -1.12789500 -0.94471900
C 8.87520900 2.51178600 -0.30172100
H 10.55920000 1.96757800 -1.56458900
C 7.70447200 2.04268300 0.28774500
C 6.12967200 0.17306300 0.66592500
H 9.20793100 3.53239100 -0.15367700
H 7.10088400 2.69318200 0.91109800
C 5.12302400 -0.25142200 1.20246900

Bowl 1^{6+} - Cis-in$_2$Cis-out

E(RB3LYP) = -3715.671842

Number of Imaginary Frequencies = 0

Cartesian Coordinates:

C -1.35812300 0.07271600 4.16693600

C 0.89198700 1.21679000 4.06483700

C 0.75575500 -1.30426100 4.05692900

C -2.73551900 -2.88217900 2.06506300

C -1.53655000 -3.65057500 1.98762500

C -0.36909400 -3.17748200 2.61959800

C -0.38342200 -1.96336500 3.29723100

C -1.56049500 -1.20289400 3.36602400

C -2.72895900 -1.65887700 2.76532400

C 3.87736200 -0.91673500 1.78714800

C 3.95953600 0.50697900 1.79901300

C 3.00266000 1.24946500 2.51993800

C 1.98861500 0.59819200 3.21357000

C 1.91004600 -0.80263300 3.20522100

C 2.84272100 -1.55561700 2.50012200

C -1.13030600 3.79295800 1.98626500

C -0.02300200 3.20059400 2.62590200

C -0.16795300 1.99305500 3.30039200

C -1.41811600 1.35938200 3.36010400

C -2.52722000 1.93239200 2.74754700

C -2.40177200 3.15005800 2.04911500

C 0.12364200 -0.00781100 4.66613900

C -0.70623100 6.09517800 0.76702700

C -0.94493000 5.03520500 1.31507000

C -4.60213500 4.13236900 0.97226400

C -3.56376200 3.71410900 1.44956000

C -5.03574000 -3.63843000 1.01451800

C -3.95523900 -3.32536000 1.47890700

C -1.35365600 -5.97232600 0.74642300

C -1.48288700 -4.89984300 1.30664000

C 0.05969600 9.77515600 -1.09823700

C 0.86183300 9.39124900 -0.03498600
C 0.63129300 8.17483000 0.60080400
C -0.41510200 7.34382300 0.15166300
C -1.18231200 7.78290500 -0.93303800
N -0.94226500 8.96990400 -1.52511100
C -0.96610200 -9.69177000 -1.15612900
N -1.90192400 -8.79710700 -1.55407700
C -2.01976100 -7.59775400 -0.95026600
C -1.18971500 -7.23663100 0.11673100
C -0.21039200 -8.16050800 0.53453900
C -0.10621500 -9.38832600 -0.11223600
C -8.77713000 -4.75002000 -0.52281800
C -8.73380000 -3.85275100 0.53186300
C -7.50480000 -3.46590500 1.05885100
C -6.31775300 -3.99066500 0.50980600
C -6.42828300 -4.88623600 -0.56091400
N -7.63141600 -5.24876800 -1.04677800
C -8.17938600 5.63703300 -0.62113800
N -6.98043500 5.97687400 -1.15260200
C -5.83134100 5.48661900 -0.64786900
C -5.83243800 4.61704400 0.44907800
C -7.07599900 4.25420700 1.00426100
C -8.24753300 4.77116800 0.45850100
C -2.77953000 -9.15358400 -2.73667200
C -7.75233500 -6.19036700 -2.23127600
C -4.11748600 -8.46186500 -2.69322100
C -4.37202500 -7.36414800 -3.52377700
C -5.56781700 -6.65169900 -3.40390700
C -6.52024100 -7.02728400 -2.44904000
C -6.29272400 -8.16783500 -1.66245200
C -5.10222200 -8.87999100 -1.78403400
C -1.75592300 9.41106100 -2.72474900
C -5.67146000 7.61426700 -2.54846200
C -4.72943900 7.16149700 -3.47952400
C -3.47449900 7.76994700 -3.56398800
C -3.14934300 8.83816300 -2.72023200
C -4.11812800 9.33493700 -1.83351600
C -5.36796500 8.72718300 -1.74789200
C -6.97581100 6.88385400 -2.36828400
H 0.53007900 -3.78372400 2.57243200
H -3.65308500 -1.09315300 2.83284600
H -2.07408700 0.11504800 4.99682300
H 3.08650300 2.33158500 2.53242400
H 2.80185300 -2.64045800 2.49675400
H 1.13068400 -1.98425700 4.83175800
H 0.93383600 3.71172400 2.58738200
H -3.50436400 1.46321100 2.80427600
H 1.33327300 1.85216300 4.84255200
H 0.17663000 -0.01444500 5.75552900
H 0.18958000 10.71159500 -1.62587800
H 1.65560300 10.05292800 0.29126800
H 1.24461800 7.86524400 1.43990800
H -1.99670600 7.19282800 -1.32937500
H -0.93363900 -10.63239400 -1.69106100
H 0.44954000 -7.91257000 1.35859900
H 0.63278000 -10.12065400 0.19094400
H -9.70276400 -5.08931800 -0.97045200
H -9.66316700 -3.46927300 0.93636800
H -7.45407700 -2.77023900 1.88931500
H -5.55721800 -5.31943500 -1.03157000
H -9.05579200 6.07429300 -1.08255600
H -7.11237400 3.58188300 1.85445100
H -9.21756500 4.51565400 0.86848200
H -8.62448900 -6.81123100 -2.02096200
H -7.96984500 -5.55979700 -3.09632700
H -3.62777900 -7.04595800 -4.24894500
H -5.74256300 -5.78620000 -4.03744100
H -7.03953500 -8.48703100 -0.94031200
H -4.92956800 -9.74963800 -1.15518100
H -4.96036500 6.31539600 -4.12114900

H -2.74014200 7.39181900 -4.27010100

H -3.88788800 10.18248600 -1.19316300

H -6.10281100 9.10522200 -1.04192400

H -7.20191000 6.24497000 -3.22497800

H -7.80573200 7.57596600 -2.21878800

H -1.19528800 9.09669900 -3.60804200

H -1.76761700 10.50110700 -2.68718200

H -2.88546900 -10.23896000 -2.70795200

H -2.21424000 -8.88060500 -3.63074600

H -4.91281400 5.79737800 -1.12492400

H -2.78673500 -6.93412600 -1.32361400

C 4.81190800 -1.72244500 1.07639900

C 5.56295400 -2.48085300 0.49166800

C 6.41327900 -3.38743700 -0.19910900

C 7.71394500 -3.00165800 -0.54536400

C 5.98985400 -4.68255100 -0.55863200

N 8.53459700 -3.83364300 -1.21558600

H 8.10294700 -2.02575200 -0.29236000

C 6.86178600 -5.52352000 -1.24449100

H 4.98891000 -5.01363800 -0.30441600

C 9.95790700 -3.43045100 -1.55237900

C 8.13322400 -5.07874900 -1.56879700

H 6.56250200 -6.52292700 -1.53776900

C 10.16190100 -1.93901600 -1.56281200

H 10.16151900 -3.86955300 -2.53009400

H 10.59349600 -3.91757200 -0.80963600

H 8.85174500 -5.68617800 -2.10465400

C 10.73406300 -1.29609900 -0.45867700

C 9.73943200 -1.17415300 -2.66161900

C 10.82293300 0.09758100 -0.42307000

H 11.08734600 -1.87880600 0.38784100

C 9.83031200 0.21499600 -2.62724700

H 9.32108900 -1.66439100 -3.53666100

C 10.34314000 0.86317100 -1.49228500

H 11.24404900 0.58653300 0.45127100

H 9.48305300 0.79750100 -3.47655200

C 10.33049300 2.36657300 -1.40463900

N 8.95615900 2.92680600 -1.09262100

H 10.62606000 2.82579600 -2.34912400

H 10.99303400 2.72919500 -0.61542500

C 8.03512000 2.18353200 -0.44848000

C 8.70212100 4.21257200 -1.43608300

C 6.77900300 2.70712800 -0.12121100

H 8.30917200 1.16818100 -0.19946400

C 7.48118600 4.79186100 -1.12956700

H 9.49370800 4.74327800 -1.95012300

C 5.82222200 1.89199400 0.54342700

C 6.50813900 4.04518900 -0.47139700

H 7.30034600 5.82135100 -1.41534300

C 4.98467500 1.21217000 1.10675000

H 5.54493900 4.48186900 -0.23120700

Bowl **1**$^{6+}$ - Cis-out$_3$

E(RB3LYP) = -3715.671731

Number of Imaginary Frequencies = 3

Cartesian Coordinates:

C -1.49621700 -10.57163000 -1.36049600

C -4.71180300 -8.86873600 -2.46725800

C -5.01110500 -9.68430800 -0.20823700

C -2.92057700 -10.08299400 -1.37184300

C -3.72944900 -10.23954900 -0.24018200

C -5.49759400 -8.96729500 -1.30774400

C -3.43414400 -9.42154900 -2.49887300

C 1.74845700 -8.68934800 -1.33830800

C 0.77739400 -9.65198900 -1.56335600

C -6.82835100 -8.26622900 -1.22868400

C -5.60604000 -6.25986100 -0.39489400

C -7.72388600 -5.97797400 -1.38073800

H -5.09083400 -8.34106800 -3.33856000

H -5.61941800 -9.78785000 0.68622200

H -3.35177300 -10.77031100 0.62961100

H -2.82653100 -9.32101500 -3.39444900

H 2.75800900 -8.86728700 -1.68963500

H -4.82628600 -6.95393500 -0.11546300

H -1.21961400 -11.02753000 -2.31234200

H -1.32649700 -11.29698500 -0.56161800

H 0.97850500 -10.58410300 -2.07602200

H -8.56258000 -6.46581900 -1.86106200

H -7.39419800 -8.37145300 -2.15552800

H -7.43429100 -8.64651600 -0.40307100

N -0.49325000 -9.45643400 -1.13651900

N -6.69446300 -6.77286700 -1.00144300

C -0.10692600 -2.01106100 2.99064500

C 1.81107400 0.90178600 2.97292800

C -1.67373700 1.10712000 3.00113900

C 0.08718300 -7.31276900 -0.24179200

C -0.63981500 -5.09141700 0.98342800

C -1.39167000 -1.44713300 3.00141400

C 1.96811800 -0.49242200 2.98089300

C -0.54300700 1.93751800 2.99079700

C -2.22810000 -3.29564300 1.66887400

C 3.98254300 -0.28471700 1.64238200

C -1.74450900 3.57734700 1.66402100

C -2.44400300 -2.08064100 2.34951600

C 3.04240700 -1.08293600 2.32440700

C -0.57463500 3.16114800 2.33100200

C 0.02074500 0.00148400 4.36505200

C -1.43276600 -0.15910000 3.80652200

C 0.87916400 -1.17556700 3.79155700

C 0.60265600 1.33498300 3.78648600

C 0.12912500 -3.20908200 2.32476400

C 2.72365300 1.70860300 2.30204300

C -2.83855800 1.49698800 2.34920400

C -0.31762800 -6.12385000 0.42536500

C 1.41286800 -7.51127300 -0.67722800

C -0.92199500 -3.86801700 1.65564800

C 3.81913200 1.13186200 1.62845700

C -2.89305000 2.73199800 1.67237500

C -0.84403400 -8.32851200 -0.48793000

C -3.33190300 -3.91508500 1.01658700

C -6.56170200 -4.05427000 -0.53143300

C -7.67674500 -4.61234700 -1.14999800

C -4.32372300 -4.37497200 0.48201600

C -5.49315000 -4.88755000 -0.14392800

H -3.44619500 -1.66375600 2.36418100

H 3.18681200 -2.15861100 2.33875300

H 0.28778600 3.82041000 2.32884900

H 1.11458300 -3.66415800 2.31878500

H 2.62080100 2.78921200 2.29754800

H -3.72498900 0.87060600 2.36033100

H 2.15948700 -6.74477100 -0.50068300

H -1.87392300 -8.24386600 -0.17164200

H -6.50675700 -2.98634200 -0.35085800

H -8.51124700 -3.99617200 -1.46351100

H 0.02923000 0.00471200 5.45572500

H -2.17744300 -0.24057300 4.60778300

H 0.91440300 2.02776000 4.57778300

H 1.32830200 -1.78530900 4.58510100

C 4.73417100 1.99054000 0.95501000

C 5.46500300 2.79049500 0.40097900

C 6.29592400 3.74132000 -0.25303500

C 5.80522000 4.98641900 -0.69485800

C 7.64661200 3.45080200 -0.47628700

C 6.66437200 5.87288700 -1.33793400

H 4.76353300 5.24325000 -0.53655100

N 8.45478900 4.32501000 -1.10770800

H 8.08757100 2.51781600 -0.15515400

C 7.98966700 5.52173800 -1.53962800
H 6.31433800 6.83489300 -1.69356300
C 9.92716100 4.02153500 -1.30555700
H 8.70148600 6.16776600 -2.03775800
H 10.19843100 4.49398800 -2.25073000
H 10.45389100 4.53015200 -0.49493600
C 10.22189400 2.54449100 -1.31816400
C 10.76019400 1.92260000 -0.18562100
C 9.91220200 1.76897200 -2.44727200
C 10.92606300 0.53566400 -0.15536700
H 11.02594700 2.51417100 0.68628700
C 10.07831400 0.38679000 -2.41728300
H 9.52179800 2.24407900 -3.34347200
C 10.55608000 -0.24394900 -1.25733200
H 11.31963100 0.06136400 0.73950800
H 9.81727200 -0.20540200 -3.29050500
C 10.62329300 -1.74674700 -1.18166300
H 11.01112500 -2.17909300 -2.10514000
H 11.24760300 -2.07998400 -0.34944400
N 9.26437200 -2.38557100 -0.97333300
C 8.26378600 -1.70448100 -0.38117500
C 9.10392500 -3.67599400 -1.35278400
H 8.46589800 -0.68076100 -0.09972400
C 7.01906000 -2.30005800 -0.14673500
H 9.95551900 -4.15363600 -1.82045500
C 7.89850300 -4.32512300 -1.13807500
C 6.84495400 -3.64363600 -0.53547400
C 5.97873300 -1.55010500 0.46776700
H 7.79292000 -5.35704100 -1.45160000
H 5.89314700 -4.13549300 -0.36742200
C 5.07575900 -0.92588000 0.99302600
C -4.10278300 3.09531500 1.01476100
C -5.16754000 3.32751100 0.47326400
C -6.41174200 3.56923100 -0.17179700
C -7.24666000 2.52013500 -0.60675200
C -6.83719700 4.88315600 -0.39680300
C -8.44654400 2.81870500 -1.24565100
H -6.94713300 1.49010600 -0.44714000
N -8.00043300 5.14395700 -1.02575200
H -6.24820200 5.73276500 -0.08165200
C -8.80598600 4.14144100 -1.45108300
H -9.10618300 2.03351300 -1.59583300
C -8.46808800 6.57156800 -1.22433700
H -9.72309200 4.43212000 -1.94750700
H -9.05341800 6.56376700 -2.14483600
H -9.13394400 6.79411900 -0.38746800
C -7.32539400 7.55034800 -1.29970900
C -7.01608400 8.36102800 -0.20128300
C -6.52921300 7.62146000 -2.45380800
C -5.88814900 9.18497500 -0.22923000
H -7.63854100 8.33276400 0.68898200
C -5.40467600 8.44216100 -2.48141400
H -6.77690300 7.01917600 -3.32388500
C -5.05743700 9.20628900 -1.35570600
H -5.64335200 9.79034600 0.63931400
H -4.78334600 8.47361300 -3.37257700
C -3.77896200 10.00080700 -1.34268500
H -3.59711500 10.48230000 -2.30472500
H -3.78791100 10.76591600 -0.56303200
N -2.55269900 9.14716100 -1.07954000
C -2.65085500 7.95537900 -0.45918200
C -1.34884500 9.64692000 -1.44938500
H -3.64248500 7.62147800 -0.18889800
C -1.51445700 7.18560200 -0.18220200
H -1.35542400 10.61105500 -1.94209700
C -0.18657200 8.93736600 -1.19441200
C -0.25821200 7.70032300 -0.56009100
C -1.64977300 5.92412100 0.45989500
H 0.76463200 9.35650500 -1.50079400
H 0.64177000 7.12926500 -0.35992200

C -1.73824900 4.83871200 1.00322700

Bowl **1**$^{6+}$ - Cis-out$_2$Trans

E(RB3LYP) = -3715.671881

Number of Imaginary Frequencies = 0

Cartesian Coordinates:

C 10.71739800 0.72519400 -1.22444500
C 9.66893500 -2.70235400 -2.49839000
C 10.52302000 -2.94590500 -0.24685300
C 10.51122700 -0.76443600 -1.30783300
C 10.81832500 -1.58095800 -0.21280900
C 9.91622900 -3.50902600 -1.37610400
C 9.96431600 -1.34172800 -2.46494400
C 8.25019800 3.54783000 -1.09641700
C 9.38478300 2.79089800 -1.34135300
C 9.49252000 -4.95367100 -1.37035700
C 7.28661100 -4.18300900 -0.48556000
C 7.45725500 -6.32949400 -1.43416100
H 9.22682200 -3.13474100 -3.39224600
H 10.74268100 -3.56470800 0.61899600
H 11.26468700 -1.14961700 0.67904200
H 9.74959000 -0.72397400 -3.33308400
H 8.23814600 4.59017900 -1.39251400
H 7.79653600 -3.26318700 -0.23764000
H 11.12570900 1.12799900 -2.15257400
H 11.38513000 0.99247400 -0.40221600
H 10.27073400 3.19271500 -1.81635200
H 8.10860500 -7.04752300 -1.91644000
H 9.66839300 -5.42616400 -2.33805200
H 10.01788000 -5.52309700 -0.60039800
N 9.42755300 1.48512700 -0.98339300
N 8.01248400 -5.14161600 -1.09261500
C 2.01887000 0.25713900 3.02464600
C -1.23371300 1.55276100 3.10341100
C -0.72664000 -1.89528500 3.01559200
C 7.19734700 1.59965400 -0.12040100
C 5.14651900 0.40012600 1.02768000
C 1.72441300 -1.11458200 3.00538400
C 0.10365200 1.97647200 3.07152600
C -1.76970500 -0.95797100 3.06013400
C 3.70081500 -1.53197600 1.65847100
C -0.53256500 3.92512100 1.77000900
C -3.15831400 -2.42213700 1.70993500
C 2.55393500 -2.00270100 2.32950400
C 0.45391800 3.14952600 2.41229800
C -2.97493000 -1.21609600 2.41636200
C 0.04256700 -0.05546600 4.42130500
C 0.47902200 -1.43187500 3.81624600
C 1.00878500 1.03602600 3.85016600
C -1.39466200 0.26084500 3.88779100
C 3.14447400 0.74392300 2.36928200
C -2.22525300 2.30781200 2.48667100
C -0.88442600 -3.09107700 2.32344500
C 6.09919300 0.94256100 0.49940100
C 7.14627000 2.95990700 -0.48561800
C 4.00005900 -0.13779100 1.67860100
C -1.89340800 3.49998400 1.81119200
C -2.09615700 -3.37261600 1.66116700
C 8.37587400 0.89234500 -0.38487700
C 4.53214800 -2.47402000 0.98839300
C 5.34653500 -5.60836900 -0.54232400
C 6.12226400 -6.58276500 -1.16432200
C 5.18847300 -3.34162800 0.44276300
C 5.93089800 -4.37482800 -0.19229100
H 2.34632200 -3.06812100 2.32031500
H 1.48201800 3.49711300 2.39232200
H -3.79602400 -0.50723800 2.45611800

H 3.39541800 1.79976600 2.39203100
H -3.26684000 2.00428800 2.52265000
H -0.09070800 -3.83112900 2.29213900
H 6.24865800 3.53741300 -0.29343900
H 8.48341000 -0.15072000 -0.12344900
H 4.29867200 -5.78934000 -0.32922900
H 5.69934400 -7.53920600 -1.44853800
H 0.06663000 -0.07552400 5.51157000
H 0.71626400 -2.16858100 4.59358300
H -2.11764900 0.40048800 4.70072100
H 1.52315300 1.58891400 4.64587200
C -2.21863700 -4.60719200 0.96247900
C -2.24001400 -5.67725100 0.38345500
C -2.23953800 -6.91848200 -0.31020600
C -1.05250900 -7.50689900 -0.79188500
C -3.44562000 -7.58896500 -0.54120200
C -1.11656000 -8.71385300 -1.48161600
H -0.10070700 -7.01348400 -0.62826500
N -3.47994900 -8.75167100 -1.22260900
H -4.38973900 -7.19660000 -0.19004300
C -2.34460000 -9.32095900 -1.69282700
H -0.22190700 -9.18720500 -1.86870900
C -4.78934800 -9.48249400 -1.43325200
H -2.45606400 -10.25404700 -2.23019000
H -4.66993100 -10.03836300 -2.36438400
H -4.88038000 -10.19432500 -0.60966600
C -5.96917400 -8.54696900 -1.48877000
C -6.84212100 -8.44951000 -0.39900100
C -6.17450400 -7.72921700 -2.61110600
C -7.87060200 -7.50433400 -0.40235900
H -6.70648600 -9.09359200 0.46559100
C -7.19795000 -6.78458300 -2.61295700
H -5.52028800 -7.81461700 -3.47492100
C -8.03484900 -6.64360800 -1.49437200
H -8.52654200 -7.42152600 0.46003400
H -7.33514400 -6.14154200 -3.47837800
C -9.06753800 -5.54990400 -1.45033200
H -9.52010700 -5.38706400 -2.42984500
H -9.85767800 -5.77470300 -0.73068300
N -8.50189500 -4.20240500 -1.03693400
C -7.29014700 -4.09296800 -0.45991100
C -9.28425400 -3.11346800 -1.23469300
H -6.72397000 -5.00383700 -0.32933900
C -6.79082000 -2.85032700 -0.04909400
H -10.24467400 -3.28653600 -1.70385700
C -8.84989300 -1.85768400 -0.84392000
C -7.59960600 -1.71394600 -0.24836500
C -5.50484400 -2.76136600 0.55092000
H -9.49227900 -1.00147500 -1.01305000
H -7.24302500 -0.73706700 0.05957500
C -4.40909600 -2.64968500 1.06883200
C -0.13006800 5.12011600 1.10980500
C 0.30428000 6.11983000 0.56832600
C 0.85244100 7.26507100 -0.07077500
C 2.23876800 7.40744000 -0.28230400
C 0.01066100 8.29019500 -0.51875400
C 2.72148700 8.54277000 -0.92665000
H 2.91824300 6.63147900 0.05287800
N 0.50445200 9.37362800 -1.14954800
H -1.06044900 8.24672600 -0.38216000
C 1.83541600 9.51652000 -1.35850500
H 3.78151400 8.67759600 -1.10676700
C -0.40573100 10.49665300 -1.60800300
H 2.15147400 10.41582500 -1.87193500
C -1.83491900 10.05734700 -1.78854900
H -0.31890000 11.28458400 -0.85633100
H 0.02423100 10.85640500 -2.54422800
C -2.20346200 9.28629200 -2.90012500
C -2.80009300 10.37661000 -0.82327200
C -3.49934000 8.78184400 -3.00615800

H -1.47220700 9.05492300 -3.67014700

C -4.10068700 9.88554200 -0.93787800

H -2.53223000 10.99489800 0.02925000

C -4.44932700 9.05431000 -2.01142200

H -3.76655300 8.15936100 -3.85589500

H -4.83486000 10.12780800 -0.17401900

C -5.81488200 8.42319000 -2.07304000

H -6.59608500 9.12858800 -1.78547500

H -6.03588300 8.04298700 -3.07285200

N -5.97034700 7.24359100 -1.13194300

C -4.89797900 6.58834200 -0.64679600

C -7.22996500 6.84719100 -0.82820100

H -3.92415500 6.95943300 -0.93249500

C -5.04502200 5.48225100 0.19944300

H -8.03707300 7.42688800 -1.25829500

C -7.44140100 5.75165600 -0.00699100

C -6.35216800 5.06054700 0.51580300

C -3.89818100 4.82562300 0.72268100

H -8.45770000 5.45390600 0.22303700

H -6.50086000 4.20654300 1.16743100

C -2.94121900 4.24845800 1.20493900

Bowl **1**$^{6+}$ - Cis-out$_2$Cis-in

E(RB3LYP) = -3715.671808

Number of Imaginary Frequencies = 1

Cartesian Coordinates:

C -1.32703200 0.48684200 3.98734000
C 1.17926100 0.79398100 3.97895300
C 0.19214800 -1.52900100 3.90021400
C -3.53843600 -1.77102300 1.73861600
C -2.68121500 -2.90984200 1.69049500
C -1.44769800 -2.87647800 2.37143800
C -1.07244900 -1.74336500 3.08491600
C -1.91603000 -0.62353100 3.13292700
C -3.13672900 -0.63327100 2.46703800
C 3.33474800 -2.19407300 1.72690000
C 3.88627900 -0.87931000 1.76118000
C 3.21481700 0.13430500 2.47430800
C 2.02336300 -0.14609900 3.13418500
C 1.47747200 -1.43800800 3.09490500
C 2.12504900 -2.45469600 2.40162600
C 0.22836000 3.96490100 1.95673400
C 1.04531900 3.01014600 2.59525700
C 0.47487400 1.90691700 3.22063400
C -0.91804100 1.73948000 3.23123400
C -1.74451900 2.67700100 2.62181200
C -1.18793300 3.79724100 1.97207800
C 0.01889500 -0.10427900 4.52560800
C 1.45736300 6.01361600 0.83502100
C 0.85007300 5.08585000 1.33643900
C -2.88980300 5.50421900 0.89716200
C -2.06870000 4.74321900 1.37450600
C 3.49118000 9.27807300 -0.84091600
C 4.09042600 8.58823100 0.20112100
C 3.43975400 7.49898000 0.77307800
C 2.17858900 7.10646500 0.28033700
C 1.63384600 7.83392900 -0.78372800
N 2.28136400 8.89182000 -1.31162900
C -5.71028300 8.17578200 -0.68648300
N -4.43913800 8.14355600 -1.15462100
C -3.53473600 7.27914800 -0.65470000
C -3.87073300 6.39091000 0.37412200
C -5.19213500 6.41496900 0.86339400
C -6.10748300 7.31383900 0.32295800
C 1.69497500 9.64891700 -2.48530900
C -2.60384400 9.31678900 -2.42403600
C -1.85612300 8.61808500 -3.37894400
C -0.46663700 8.75548600 -3.42419800
C 0.18968000 9.59401500 -2.51597200
C -0.56643800 10.34495100 -1.60171700
C -1.95139800 10.20733000 -1.55602300
C -4.08469000 9.07733200 -2.29667400
H -0.81017500 -3.75430800 2.33624500
H -3.80364600 0.22231500 2.50499900
H -2.01978000 0.74491000 4.79765200
H 3.65480000 1.12623000 2.50662500
H 1.72380000 -3.46312100 2.37950000
H 0.28226000 -2.30961400 4.66553300
H 2.12002800 3.16275600 2.59623000
H -2.82478700 2.57164100 2.64548900
H 1.78819700 1.21912000 4.78631800
H 0.02781800 -0.14464400 5.61554100
H 3.94633600 10.13701100 -1.31744900
H 5.05760000 8.91738000 0.56261400
H 3.89079600 6.95605800 1.59643100
H 0.67572300 7.57952100 -1.21474900
H -6.37629000 8.89847000 -1.14110000
H -5.48535000 5.73931700 1.65939900
H -7.12867600 7.35889000 0.68271600
H -2.35164700 7.94381000 -4.07221400
H 0.10614800 8.18736900 -4.15209500
H -0.07049400 11.02150700 -0.91059600
H -2.52465100 10.77798900 -0.82998500
H -4.49683200 8.62073200 -3.19942900
H -4.62405800 10.00377800 -2.09460900
H 2.13153600 9.20762600 -3.38426100
H 2.05913900 10.67234900 -2.38616800
H -2.54163300 7.30602100 -1.07998400
C 3.97118300 -3.26334000 1.03463200
C 4.44798100 -4.22870800 0.46745000
C 4.98137000 -5.36502900 -0.20071000
C 6.35409400 -5.44003800 -0.46281000

C 4.16841000 -6.43759400 -0.61979500
N 6.88597200 -6.49570100 -1.11037400
H 7.03328700 -4.65539300 -0.16099100
C 4.74603800 -7.51880400 -1.27887000
H 3.10069200 -6.41192000 -0.43152100
C 8.37970200 -6.58561500 -1.35097100
C 6.11082200 -7.52838900 -1.51964800
H 4.14580600 -8.35540900 -1.61672500
C 9.03924000 -5.23321300 -1.42709800
H 8.49419500 -7.14320500 -2.28169700
H 8.78550500 -7.18437800 -0.53236500
H 6.61307800 -8.33967700 -2.03091200
C 9.79057800 -4.75480200 -0.34738700
C 8.86726000 -4.42131700 -2.55993100
C 10.30929100 -3.45795400 -0.36959700
H 9.94969800 -5.38254600 0.52517900
C 9.38132500 -3.12718400 -2.58031400
H 8.31147700 -4.79235600 -3.41730700
C 10.07920400 -2.62454000 -1.47037600
H 10.86894300 -3.08785600 0.48526500
H 9.22321500 -2.49857000 -3.45284900
C 10.53240900 -1.18938900 -1.44346900
N 9.41552200 -0.21870100 -1.10749500
H 10.91218300 -0.87088800 -2.41544300
H 11.30876400 -1.02738500 -0.69242000
C 8.30788800 -0.62471400 -0.45677700
C 9.59147600 1.08144100 -1.44590800
C 7.29227600 0.27809600 -0.11948400
H 8.23673300 -1.67495900 -0.21248700
C 8.62929500 2.02575300 -1.12681500
H 10.50829400 1.32600300 -1.96723900
C 6.12072200 -0.18337600 0.54127600
C 7.47062800 1.63319300 -0.46254400
H 8.79139900 3.05954200 -1.40855900
C 5.09939000 -0.55005000 1.09220700
H 6.70349800 2.35836700 -0.21416800
C -4.79765400 -1.74301600 1.07425100
C -5.88375100 -1.63558200 0.53580000
C -7.14987800 -1.48141400 -0.09285700
C -7.91339700 -2.61052300 -0.41214500
C -7.67753900 -0.21401100 -0.41158300
N -9.11236500 -2.49554400 -1.01609400
H -7.56852100 -3.61026000 -0.18987100
C -8.92118000 -0.12512900 -1.03050200
H -7.11160300 0.68101000 -0.17748500
C -9.95799200 -3.71798300 -1.31641200
C -9.62501700 -1.28097300 -1.32804200
H -9.35088800 0.83494600 -1.29125500
C -9.14509900 -4.98082700 -1.42357800
H -10.47714700 -3.49234500 -2.24912800
H -10.69426600 -3.78298400 -0.51203200
H -10.59463800 -1.27375000 -1.80940200
C -9.10265100 -5.88276100 -0.35405600
C -8.38460100 -5.24171900 -2.57488800
C -8.26190900 -6.99729100 -0.40483700
H -9.70470100 -5.70343900 0.53259500
C -7.54652400 -6.35276200 -2.62521200
H -8.43132100 -4.56453400 -3.42388000
C -7.45427100 -7.22136100 -1.52585400
H -8.21775200 -7.67551500 0.44301300
H -6.94672100 -6.53439000 -3.51309000
C -6.46027900 -8.35294800 -1.53221400
N -5.03893300 -7.88944800 -1.28076900
H -6.43562200 -8.86098300 -2.49722300
H -6.68295500 -9.08766600 -0.75522800
C -4.79142000 -6.75786800 -0.59194600
C -4.02765700 -8.67259700 -1.72709400
C -3.48189800 -6.34167400 -0.32562900
H -5.64697700 -6.18614800 -0.26148100
C -2.70946900 -8.32226900 -1.48222200
H -4.30886600 -9.56431900 -2.27278000
C -3.25239300 -5.12877300 0.38047300
C -2.42412300 -7.15350900 -0.78229500
H -1.91693400 -8.96329900 -1.85001500
C -3.03084700 -4.08912200 0.97292100
H -1.39746100 -6.86042400 -0.59211700

Bowl $\mathbf{1}^{6+}$ - Mixed (TransCis-outCis-in)

E(RB3LYP) = -3715.671789

Number of Imaginary Frequencies = 3

Cartesian Coordinates:

C 1.07161900 0.99409800 3.98614500

C 0.42255400 -1.44600800 3.94360300

C -1.36598100 0.33454300 4.02987000

C -0.34019500 3.97395600 1.94384800

C -1.71550100 3.59689900 1.96004400

C -2.10076700 2.41487600 2.62441100

C -1.14545600 1.61783600 3.24569500

C 0.20825100 1.98609900 3.22482800

C 0.60921800 3.15537500 2.58805000

C -3.27997500 -2.26169100 1.87527200

C -2.26567200 -3.26293700 1.81610700

C -1.03674900 -3.04143300 2.47114300

C -0.81270500 -1.85299800 3.15731400

C -1.80771000 -0.86490900 3.20802600

C -3.03122100 -1.06552400 2.57834700

C 3.59105100 -1.68298200 1.72022400

C 2.44041600 -2.10434800 2.41624200

C 1.66883200 -1.18247000 3.11516500

C 2.02834900 0.17348200 3.13655500

C 3.15951100 0.61163000 2.45630800

C 3.95501300 -0.30427900 1.73866400

C 0.05659800 -0.05264700 4.55651900

C 0.57352300 6.18207700 0.82436500

C 0.10822900 5.17345400 1.32160200

C -3.64502200 5.01348500 0.84852200

C -2.72475200 4.39093400 1.34514300

C -6.81008500 7.20162400 -0.82200900

N -5.54010300 7.36083500 -1.26600300

C -4.52381400 6.65181200 -0.73666000

C -4.73998100 5.73441800 0.29832700

C -6.05904100 5.56027600 0.76334500

C -7.09042000 6.30032200 0.19251200

C 2.15951500 9.70568500 -0.80867700

C 2.87473300 9.05437600 0.18312100

C 2.37287800 7.88072400 0.73872300

C 1.14248800 7.36706700 0.28235200

C 0.47426400 8.06496700 -0.73081000

N 0.97991900 9.20225500 -1.24620600

C -5.30677500 8.31953000 -2.41723400

C 0.27412600 9.93827700 -2.36977600

C -3.88932600 8.82494900 -2.48203600

C -2.99041200 8.28768200 -3.41111300

C -1.64916000 8.67739500 -3.40042900

C -1.19187900 9.60816200 -2.45997200

C -2.10692300 10.19484900 -1.57119200

C -3.44457900 9.80696600 -1.58239400

H -3.15365900 2.15220100 2.64947100

H 1.64947700 3.46565200 2.58587600

H 1.62434900 1.51053100 4.78054100

H -0.28201800 -3.82093400 2.43607400

H -3.81843700 -0.31977800 2.63029400

H -2.07581700 0.51214100 4.84704600

H 2.18223300 -3.15856900 2.40438800

H 3.45752400 1.65519600 2.47536600

H 0.63764000 -2.19439100 4.71622000

H 0.08341200 -0.07896900 5.64658500

H -7.57038100 7.80695700 -1.29904900

H -6.26095800 4.85698700 1.56379100

H -8.11353900 6.19167000 0.53268900

H 2.49620500 10.62399900 -1.27317000

H 3.81478300 9.47630000 0.51874200

H 2.91644500 7.36403000 1.52209800

H -0.46916400 7.72123700 -1.13034700

H 0.43471700 10.99852400 -2.16916700

H 0.80381200 9.66998700 -3.28673600

H -3.32810700 7.54444600 -4.12823800

H -0.95503200 8.23450200 -4.10955400

H -1.76893600 10.94062400 -0.85627900

H -4.13990100 10.25351300 -0.87626400

H -6.01681800 9.13412900 -2.26789900

H -5.58144700 7.77675800 -3.32469300

H -3.53816300 6.82641900 -1.14378900

C -4.55330600 -2.43601800 1.26386400

C -5.66607700 -2.50153500 0.77531100

C -6.96123600 -2.54151900 0.19154100

C -7.75180900 -1.38285800 0.04806300

C -7.48592500 -3.75496600 -0.26790300

C -9.00807100 -1.47824500 -0.54235200

H -7.37413200 -0.42663100 0.39326400

H -6.93461200 -4.68078700 -0.18363900

N -8.70135200 -3.81637100 -0.84762700

C -9.46612400 -2.70738100 -0.98995400

H -9.63633100 -0.60437300 -0.66893500

C -9.27184200 -5.13948600 -1.31559300

H -10.43049900 -2.83962600 -1.46395200

C -8.20454100 -6.14335100 -1.66664500

H -9.91232500 -5.50041300 -0.50774300

H -9.89718400 -4.90012000 -2.17691600

C -7.43053700 -5.98020000 -2.82451000

C -7.94471900 -7.22429400 -0.81328400

C -6.36472400 -6.84131600 -3.08329900

H -7.63817500 -5.16274900 -3.51020200

C -6.89058500 -8.09742800 -1.08268100

H -8.55371600 -7.37488600 0.07402600

C -6.06920300 -7.88906900 -2.19914900

H -5.74988600 -6.68531700 -3.96577700

H -6.68953100 -8.92163900 -0.40320000

C -4.85248700 -8.74864600 -2.41764500

H -5.06269500 -9.79967500 -2.21387100

H -4.47355800 -8.65827500 -3.43800500

N -3.69619200 -8.38410000 -1.50532500

C -3.62825000 -7.17753400 -0.91049700

C -2.71417200 -9.30304200 -1.34008300

C -2.54912600 -6.83347700 -0.08694500

H -4.44557600 -6.49425100 -1.09164600

C -1.61412100 -9.01814000 -0.54745600

H -2.84346300 -10.24791100 -1.85282200

C -1.52263400 -7.78358600 0.08846600

C -2.51281900 -5.56395600 0.55037100

H -0.84224000 -9.76930400 -0.42777700

H -0.67278200 -7.54884600 0.72000300

C -2.44899000 -4.49120200 1.12187800

C 4.35737600 -2.65768700 1.02014300

C 4.95419500 -3.55161900 0.44942500

C 5.62606800 -4.61327800 -0.21679500

C 6.98908100 -4.49966300 -0.51420100

C 4.96271300 -5.79757100 -0.59578500

N 7.64942200 -5.48452600 -1.15428700

H 7.55749900 -3.62064400 -0.24574900

C 5.67145600 -6.80104000 -1.25024700

H 3.90662300 -5.91754500 -0.38034600

C 9.13653700 -5.37598700 -1.43201600

C 7.01835900 -6.62490700 -1.52395600

H 5.18712100 -7.72029500 -1.55795200

C 9.62428300 -3.95182000 -1.47249800

H 9.29320600 -5.88181200 -2.38589200

H 9.63366800 -5.94818600 -0.64545600

H 7.61904100 -7.36886900 -2.03193700

C 10.29480900 -3.40459100 -0.37246100

C 9.37071600 -3.14626300 -2.59451000

C 10.64758800 -2.05265000 -0.36362700

H 10.51977400 -4.02414200 0.49150900

C 9.72278400 -1.79907200 -2.58568600

H 8.87925600 -3.56778400 -3.46762900

C 10.33389500 -1.23400800 -1.45469000

H 11.14337900 -1.63231000 0.50716400

H 9.50372900 -1.18053900 -3.45214600

C 10.60243100 0.24711100 -1.39454100

N 9.35067300 1.06008500 -1.12903700

H 11.00021900 0.62280500 -2.33839300

H 11.30309200 0.49601400 -0.59432000

C 8.30463800 0.52297000 -0.47070100

C 9.33648900 2.35446000 -1.52799100

C 7.15975000 1.27693000 -0.18818900

H 8.38915500 -0.51387400 -0.17764900

C 8.23777700 3.15701800 -1.26510100

H 10.21547400 2.71065000 -2.05005900

C 6.06129800 0.67526200 0.48549500

C 7.13924300 2.62613100 -0.59536900

H 8.24854700 4.18917400 -1.59491700

C 5.10337800 0.18238800 1.05148900

H 6.26832300 3.23898400 -0.39029700

Bowl **2**$^{6+}$ - Trans$_3$

E(RB3LYP) = -3715.675716

Number of Imaginary Frequencies = 0

Cartesian Coordinates:

C -0.08117300 -1.57481200 3.80301100

C -1.25788200 -0.29817000 5.63653400
C 2.89493100 -0.48691300 1.57271100
C 3.64159600 0.21829000 2.56093700
C 3.15741000 0.28056800 3.88146800
C 1.94980700 -0.32700000 4.21077600
C 1.20809600 -1.00864600 3.23475600
C 1.67792500 -1.10064600 1.92972900
C 0.85958200 3.25208300 6.87864500
C 1.50633000 2.07205800 6.46158600
C 0.75706800 0.96937500 6.06704300
C -0.64446600 1.01376100 6.09937300
C -1.30338400 2.16550700 6.51813500
C -0.56480500 3.29967500 6.90508700
C -3.74919100 0.44846000 2.76309000
C -3.20638100 0.45023600 4.06322000
C -2.01043900 -0.21065600 4.31992400
C -1.34782700 -0.90372800 3.29629200
C -1.86735200 -0.92007800 2.00559700
C -3.06611600 -0.23992700 1.71854500
C -0.02987300 -1.21902700 5.32717500
H 3.74565100 0.80356700 4.62882500
H 1.12467200 -1.64216300 1.16870100
H -0.13262700 -2.65671900 3.62990500
H -3.74137500 0.97327900 4.84975300
H -1.36741000 -1.45555000 1.20477400
H -1.90342100 -0.71305000 6.42018200
H -0.03861100 -2.11678100 5.94635400
H 2.59151300 2.04703900 6.45542800
H -2.38702800 2.21072100 6.55803600
C 1.26510200 -0.37168200 5.56696500
H 1.92885600 -0.82921000 6.31073200
C -1.25941800 4.47436500 7.31495300
C -1.90052000 5.45325600 7.64616500
C -2.65172000 6.59039800 8.05591700
C -2.38171600 7.26741400 9.26334900
C -3.69735500 7.06222000 7.25649300
C -3.15715600 8.36361600 9.62461300
H -1.57626000 6.92501900 9.90368200
N -4.43452200 8.12905200 7.63265000
H -3.95767400 6.59817800 6.31390300
C -4.18535000 8.78020600 8.79296300
H -2.97719800 8.89934000 10.54919000
C -5.50082800 8.65655500 6.71364600
H -4.82081000 9.62539500 9.02308300
C -6.41316400 7.59059300 6.15005800
H -4.98619000 9.19407000 5.91366500
H -6.06451700 9.38392500 7.30067200
C -7.11179500 6.70773400 6.98481900
C -6.61879000 7.52480200 4.76576700
H -6.96903600 6.74005400 8.06183600
C -7.99144700 5.77127000 6.44058100
C -7.52149100 6.60859700 4.22385500
H -6.08265700 8.20029100 4.10464000
C -8.21296400 5.72079400 5.05738500
H -8.51704500 5.08576300 7.09982300
H -7.67420000 6.58959700 3.14809500
C -9.24380500 4.75636300 4.50764900
N -8.77545100 4.01938000 3.28965500
H -9.51657100 4.00479900 5.25208100
H -10.15467300 5.28157200 4.21351800
C -9.49051700 4.08841300 2.14260500
C -7.65290100 3.27394700 3.37192500
C -9.07618700 3.39000700 1.01735500
H -10.37988000 4.70470400 2.15810800
C -7.18342000 2.54930500 2.27190300
H -7.13959600 3.27250100 4.32487700
C -7.92196700 2.61720400 1.07090200
H -9.66258200 3.46118300 0.10897000
C -5.99154900 1.78766200 2.39633500
H -7.58437300 2.06742700 0.19923300

C -4.96171800 1.15309300 2.52635400
C 4.87089100 0.86413000 2.24085200
C 5.91727200 1.44307900 2.01983100
C 7.15431800 2.09910200 1.76537600
C 8.22573000 1.44611600 1.12129000
C 7.33607200 3.42714000 2.16329900
C 9.41836200 2.12880500 0.90898400
H 8.11375200 0.41579900 0.80188500
N 8.50539900 4.06544500 1.94404200
H 6.55717000 3.99303100 2.65782100
C 9.53985000 3.44399800 1.33066100
H 10.26082500 1.65136000 0.42268500
C 8.64050200 5.51648300 2.31359900
H 10.44273900 4.02409800 1.19084600
C 8.14446800 5.85174800 3.70206000
H 8.09510200 6.08364600 1.55583600
H 9.70154900 5.75058300 2.21171900
C 8.64892100 5.20154100 4.83658700
C 7.21270700 6.88507100 3.86780400
H 9.37364500 4.39806600 4.73401800
C 8.22077800 5.57533600 6.11087300
C 6.80690900 7.27879000 5.14391600
H 6.81095000 7.39796000 2.99806000
C 7.30609500 6.62440600 6.27705900
H 8.61369900 5.05505700 6.98025400
H 6.09713100 8.09536200 5.24535900
C 6.93864200 7.06892900 7.67769300
N 5.46740300 7.28116300 7.87013900
H 7.25502700 6.33236500 8.41930700
H 7.41603700 8.01901500 7.92523700
C 5.01411000 8.47621100 8.31544800
C 4.62165100 6.25859100 7.62057600
C 3.65676400 8.67676800 8.52151000
H 5.75619300 9.24310100 8.49504200
C 3.24261600 6.40101200 7.80466900
H 5.06456400 5.33554200 7.26849100
C 2.76117200 7.64476100 8.26748200
H 3.31547400 9.64210200 8.87635400
C 2.37960600 5.31199900 7.51306700
H 1.69689100 7.78639400 8.41969200
C 1.64914000 4.37826400 7.23996500
C -3.57218500 -0.25602100 0.38670300
C -3.95284900 -0.29195900 -0.76779800
C -4.41690100 -0.36122900 -2.11114400
C -5.74946000 -0.70371800 -2.42140200
C -3.54164200 -0.09612500 -3.16900700
C -6.14783500 -0.77751700 -3.75148200
H -6.45080800 -0.91356500 -1.62131800
N -3.95595800 -0.17574300 -4.45160200
H -2.50766800 0.18009200 -3.00655100
C -5.23154100 -0.51102500 -4.75727300
H -7.16275700 -1.04528700 -4.02077400
C -3.00992600 0.18767900 -5.56255200
H -5.48512900 -0.55308500 -5.80844800
C -1.63990100 -0.44042200 -5.43882300
H -2.94121400 1.27795800 -5.56983000
H -3.50164500 -0.12516700 -6.48534900
C -1.47653200 -1.82738500 -5.31978300
C -0.50246000 0.37431100 -5.51427400
H -2.34278700 -2.48161400 -5.26307900
C -0.19794200 -2.38366500 -5.26534900
C 0.77608700 -0.18501300 -5.49211400
H -0.61119100 1.45167400 -5.60561500
C 0.93920200 -1.56983200 -5.36207100
H -0.08834100 -3.45970700 -5.16079500
H 1.64042300 0.46880400 -5.57122000
C 2.31010200 -2.21422500 -5.39296700
N 3.30983800 -1.52362400 -4.51541900
H 2.26353200 -3.25552600 -5.06642100
H 2.72899600 -2.19880000 -6.40104100

C 4.46138500 -1.05089000 -5.04692500

C 3.04779000 -1.40945800 -3.19585300

C 5.40185200 -0.42956100 -4.23765200

H 4.59816400 -1.18308200 -6.11223600

C 3.95515700 -0.79095600 -2.32985900

H 2.10456200 -1.81314900 -2.85046300

C 5.15771800 -0.29301600 -2.87614600

H 6.31534900 -0.05574300 -4.68484200

C 3.63691700 -0.67538200 -0.95103700

H 5.88139200 0.19277300 -2.23099900

C 3.33466000 -0.57390800 0.22303100

Bowl **2**$^{6+}$ - Trans$_2$Cis-in

E(RB3LYP) = -3715.673636

Number of Imaginary Frequencies = 0

Cartesian Coordinates:

C 0.04989900 -1.62129200 3.88504300

C -1.14766100 -0.32237300 5.68931800

C 2.96907300 -0.49421400 1.60440400

C 3.70456000 0.25108800 2.56858200

C 3.23507800 0.32326400 3.89495400

C 2.05111600 -0.31434700 4.25113900

C 1.32282800 -1.03913300 3.29739500

C 1.78047400 -1.14215700 1.98813400

C 0.90370100 3.28818900 6.86630100

C 1.57214600 2.11431700 6.46637600

C 0.84364900 0.99059800 6.09308400

C -0.55849100 1.00804200 6.12986700

C -1.23854600 2.15340000 6.53217400

C -0.52117600 3.30813100 6.89767600

C -3.62115200 0.36036500 2.78228400

C -3.09379900 0.38104800 4.08919600

C -1.89737200 -0.26994400 4.36852200

C -1.22502500 -0.97935100 3.36325600

C -1.73242900 -1.01944300 2.06836400

C -2.92467000 -0.34085200 1.75650400

C 0.09782900 -1.22695900 5.39926300

H 3.81525300 0.88052700 4.62367100

H 1.23560800 -1.71568300 1.24484000

H 0.01763700 -2.70753500 3.73670700

H -3.63864700 0.91507100 4.86144400

H -1.22161200 -1.56383100 1.28074400

H -1.78810900 -0.73517400 6.47823400

H 0.10844900 -2.10847600 6.04131500

H 2.65759500 2.11028800 6.45663900

H -2.32276300 2.17782600 6.57560400

C 1.37677300 -0.34796500 5.61372600

H 2.05474900 -0.77812200 6.36101900

C -3.39227900 -0.35527200 0.40874800

C -3.71207600 -0.38634000 -0.76379400

C -4.07933300 -0.43028300 -2.13963200

C -4.71532300 -0.45974500 -4.83802700

C -5.14139600 -1.23214100 -2.60537500

C -3.37280800 0.33342500 -3.07273700

N -3.69787000 0.30799900 -4.38301500

C -5.45094900 -1.24226700 -3.96109700

H -5.70684700 -1.83653700 -1.90462000

H -2.54618100 0.97187500 -2.78802900

H -6.25874300 -1.85153300 -4.34885300

H -4.91566700 -0.42309300 -5.90080000

C 3.38606900 -0.58499200 0.24245600

C 3.64851100 -0.69883100 -0.93914900

C 3.92526400 -0.84652900 -2.32909000

C 4.36423800 -1.08343700 -5.05679300

C 3.28936600 -0.01494500 -3.25430900

C 4.81280300 -1.82667300 -2.81813200

C 5.02456100 -1.93918900 -4.18810200

N 3.51528800 -0.14416700 -4.57902600
H 2.59464700 0.75795300 -2.95140100
H 5.32021900 -2.48764900 -2.12410900
H 5.69745700 -2.68545800 -4.59356300
H 4.49465700 -1.12176000 -6.13036100
H -1.43950400 -0.93245900 -6.16818700
C -0.83893800 -0.10532900 -5.79891100
C 0.73174000 2.02259200 -4.90716800
C 0.54994200 -0.19674400 -5.84624100
C -1.45963000 1.05583400 -5.30809900
C -0.66289200 2.11422300 -4.85897500
C 1.34977000 0.87074600 -5.40471400
H 1.01050600 -1.09382700 -6.25138700
H -1.12526600 3.02445300 -4.48675900
H 1.33370100 2.86277100 -4.57109900
C 2.85589500 0.81652400 -5.53324100
H 3.30275900 1.79496900 -5.34503500
H 3.15215300 0.49455600 -6.53225400
C -2.96423900 1.20754800 -5.34243000
H -3.26128800 2.22913400 -5.09562700
H -3.35798300 0.97069500 -6.33161900
C -1.23691800 4.47592000 7.29066000
C -1.89487500 5.44831300 7.60755800
C -2.66311700 6.57953500 8.00187000
C -2.40990000 7.26968800 9.20540100
C -3.70817500 7.03226300 7.19074100
C -3.20073100 8.35970000 9.55175700
H -1.60511100 6.94214000 9.85425700
N -4.46052500 8.09346300 7.55244800
H -3.95559200 6.55728300 6.25010600
C -4.22746300 8.75712600 8.70910100
H -3.03377600 8.90520800 10.47305800
C -5.52608200 8.60164800 6.62115900
H -4.87416200 9.59687400 8.92748200
C -6.41549400 7.52007500 6.05102100
H -5.01174800 9.14527700 5.82514900
H -6.10723400 9.32130600 7.20052300
C -7.12029300 6.63818300 6.88167400
C -6.59368700 7.43763900 4.66379700
H -6.99826400 6.68336000 7.96076500
C -7.97983800 5.68708500 6.33078100
C -7.47582000 6.50594100 4.11449900
H -6.05279000 8.11269100 4.00612800
C -8.17441000 5.62015300 4.94423800
H -8.51123100 5.00318200 6.98702700
H -7.60755400 6.47336800 3.03625800
C -9.18664400 4.64175400 4.38516300
N -8.68889200 3.89344100 3.18578400
H -9.47028600 3.89733300 5.13264500
H -10.09446300 5.15723200 4.06575400
C -9.38912900 3.93408400 2.02828200
C -7.55734900 3.16522500 3.29554600
C -8.94972500 3.22389400 0.92010300
H -10.28718800 4.53779400 2.02210800
C -7.06347400 2.42902700 2.21396200
H -7.05702700 3.18670300 4.25520500
C -7.78603000 2.46801300 1.00194500
H -9.52451900 3.27256600 0.00287900
C -5.86520400 1.68281500 2.36671300
H -7.42914100 1.90870900 0.14412200
C -4.83316700 1.05720200 2.52049700
C 4.90933500 0.92812300 2.21932800
C 5.93633800 1.53141600 1.97364600
C 7.15495800 2.20971800 1.69071800
C 8.22391800 1.57335000 1.02627200
C 7.32235700 3.54178800 2.08180600
C 9.40113800 2.27463200 0.79012900
H 8.12237300 0.54078000 0.71078900
N 8.47689300 4.19834700 1.83933200
H 6.54400000 4.09584100 2.59025400

C 9.50956300 3.59216400 1.20786700

H 10.24179000 1.80988900 0.28864500

C 8.59682600 5.65264100 2.20193400

H 10.40058500 4.18605400 1.05089200

C 8.10751500 5.98911900 3.59245700

H 8.03919200 6.20970600 1.44558500

H 9.65424600 5.89899800 2.09122800

C 8.62745500 5.35014500 4.72636400

C 7.16653400 7.01378400 3.76023500

H 9.35973800 4.55373800 4.62230000

C 8.20541200 5.72616900 6.00204800

C 6.76663300 7.40993400 5.03745100

H 6.75289100 7.51811800 2.89105700

C 7.28119900 6.76654800 6.17002100

H 8.61048900 5.21447700 6.87094300

H 6.04947900 8.21985700 5.14027800

C 6.91900700 7.21407600 7.57106900

N 5.44638700 7.40598600 7.77425400

H 7.25183700 6.48640700 8.31433700

H 7.38440600 8.17242700 7.80940200

C 4.97882500 8.59715500 8.21518900

C 4.61415000 6.36896600 7.53968800

C 3.62050900 8.77828200 8.43256800

H 5.71063100 9.37657400 8.38251000

C 3.23493700 6.49120100 7.73658100

H 5.06769900 5.45023600 7.18994800

C 2.73863100 7.73063700 8.19508200

H 3.26765500 9.74079100 8.78371800

C 2.38738200 5.38554100 7.46313600

H 1.67386000 7.85666200 8.35723600

C 1.67262300 4.43498500 7.20696000

Bowl $\mathbf{2}^{6+}$ - $Trans_2Cis$-out

E(RB3LYP) = -3715.677177

Number of Imaginary Frequencies = 0

Cartesian Coordinates:

C -0.01199900 -1.64257700 4.05904800
C -1.20105300 -0.29083200 5.83069700
C 2.90273800 -0.54248000 1.75452600
C 3.64056000 0.21648500 2.70927200
C 3.17547500 0.30153600 4.03526200
C 1.99285000 -0.33358800 4.40166200
C 1.26174900 -1.07161200 3.45953400
C 1.71684100 -1.19111100 2.15120400
C 0.88776600 3.31056700 6.96992400
C 1.54369000 2.12299200 6.59077400
C 0.80298000 1.00422500 6.22679900
C -0.59902000 1.03967900 6.25346000
C -1.26690700 2.19891000 6.63614400
C -0.53687100 3.34908400 6.99084900
C -3.65896100 0.35700300 2.90220600
C -3.13026800 0.40606700 4.20747400
C -1.94554500 -0.25808900 4.50652000
C -1.28477200 -1.00836500 3.52261200
C -1.79938300 -1.08699200 2.23335800
C -2.97994500 -0.39509600 1.89902700
C 0.03521400 -1.21575900 5.56458000
H 3.75640200 0.86751700 4.75648700
H 1.17138900 -1.77377400 1.41546900
H -0.04406300 -2.73159000 3.93343600
H -3.66462800 0.97244800 4.96366300
H -1.30194600 -1.66754900 1.46320800
H -1.85019700 -0.68255200 6.62335700
H 0.03312100 -2.08293200 6.22590700
H 2.62901600 2.10454800 6.58960300
H -2.35090200 2.23787800 6.67319700
C 1.32211700 -0.34609300 5.76580200
H 1.99856700 -0.77276700 6.51646800
C -3.44073800 -0.44568200 0.55416200
C -3.74510000 -0.51405800 -0.62186400
C -4.06846500 -0.58561100 -2.00317300
C -4.60861300 -0.79554100 -4.71743500
C -4.97775900 0.30421100 -2.61431800
C -3.46481100 -1.55981200 -2.80512800
N -3.74147800 -1.64771900 -4.12316000
C -5.23839300 0.19298600 -3.97506100
H -5.46276000 1.07065700 -2.02001100
H -2.75846500 -2.27711400 -2.40735600
H -5.92767400 0.86541600 -4.47195000
H -4.77662900 -0.93154500 -5.77776000
C 3.31506700 -0.63669500 0.39649200
C 3.59432000 -0.73653300 -0.78340900
C 3.89886100 -0.84901400 -2.16593300
C 4.40853100 -1.14288000 -4.87786000
C 3.19221200 -1.76010100 -2.95795200
C 4.89511100 -0.06559100 -2.78672700
C 5.14018700 -0.21842300 -4.14637500
N 3.45532100 -1.89068400 -4.27483300
H 2.41365100 -2.39286100 -2.55172300
H 5.45955900 0.65080600 -2.20009400
H 5.89603100 0.37178900 -4.65090400
H 4.56178000 -1.30786700 -5.93624400
H -1.45848300 -4.61094300 -3.81898300

C -0.91079200 -3.80459000 -4.29949800
C 0.52066400 -1.76511300 -5.55668700
C 0.48668200 -3.84935400 -4.33344500
C -1.60106300 -2.74532500 -4.89745300
C -0.87084100 -1.72107700 -5.52390000
C 1.21335600 -2.83470200 -4.96463800
H 1.00530300 -4.68894100 -3.87811200
H -1.38759300 -0.89639000 -6.00796800
H 1.06639600 -0.97381300 -6.06425800
C 2.71856200 -2.93766700 -5.06758100
H 3.05074100 -2.82042600 -6.09983800
H 3.07218700 -3.90544600 -4.70508300
C -3.11369400 -2.75405800 -4.92735400
H -3.48974400 -2.63546400 -5.94429500
H -3.50936500 -3.69046700 -4.52851500
C -1.23874800 4.53186800 7.36329600
C -1.88200500 5.51934300 7.66301700
C -2.63540100 6.66773400 8.03563800
C -2.38916000 7.36376700 9.23718500
C -3.65916700 7.13162200 7.20410600
C -3.16588500 8.47061100 9.56148100
H -1.60085400 7.02760700 9.90167900
N -4.39861100 8.20865400 7.54488600
H -3.90061200 6.65255100 6.26400200
C -4.17225100 8.87837100 8.69937700
H -3.00388600 9.02113200 10.48068300
C -5.44150100 8.72433100 6.59254400
H -4.80755700 9.73101200 8.90058600
C -6.34168600 7.65065600 6.02441100
H -4.90678400 9.24878600 5.79711600
H -6.01805300 9.46131000 7.15453000
C -7.07087200 6.79182900 6.85799500
C -6.50443300 7.55250600 4.63626300
H -6.96046000 6.84903800 7.93774900
C -7.94076100 5.84896900 6.30944300
C -7.39642400 6.62907900 4.08877400
H -5.94403900 8.20930100 3.97642500
C -8.12097100 5.76750800 4.92180900
H -8.49208300 5.18360700 6.96831300
H -7.51678900 6.58463000 3.00963900
C -9.14794500 4.80373000 4.36490000
N -8.65091300 4.01557900 3.19074500
H -9.46490500 4.08426600 5.12291200
H -10.03584700 5.33550000 4.01773600
C -9.33046700 4.05422700 2.02086300
C -7.54379600 3.25588200 3.33453600
C -8.89609200 3.30803300 0.93476200
H -10.20870700 4.68558100 1.98745900
C -7.05349000 2.48632400 2.27450800
H -7.05991400 3.27984300 4.30265300
C -7.75666100 2.52067600 1.05096200
H -9.45459200 3.35461700 0.00741200
C -5.87513900 1.71483600 2.45477500
H -7.40322700 1.93373200 0.21056000
C -4.85701900 1.07000500 2.62202400
C 4.84456200 0.88836400 2.34951500
C 5.87260200 1.48458200 2.09124900
C 7.09306000 2.15185300 1.79066800
C 8.15164000 1.50095700 1.12357000
C 7.27292700 3.48683700 2.16580000
C 9.33117400 2.19179400 0.86884400
H 8.04041600 0.46566100 0.82040000
N 8.42956800 4.13279500 1.90573300
H 6.50310000 4.05172500 2.67533900
C 9.45219800 3.51300600 1.27117100
H 10.16401000 1.71602000 0.36466700
C 8.56333400 5.58994900 2.25240100
H 10.34560600 4.09931200 1.09971600
C 8.08572900 5.94408100 3.64250600
H 8.00513300 6.14354600 1.49390200
H 9.62195500 5.82645800 2.13246300
C 8.61438600 5.31832800 4.77982800
C 7.14667200 6.97119100 3.80557100
H 9.34595400 4.52076900 4.67962100
C 8.20214900 5.70925300 6.05417100
C 6.75695700 7.38261600 5.08112700
H 6.72659700 7.46557000 2.93376500
C 7.27954400 6.75188300 6.21711300
H 8.61362000 5.20752600 6.92585900
H 6.04152000 8.19455500 5.17973300
C 6.92790700 7.21490400 7.61581700
N 5.45717600 7.41264500 7.82693000
H 7.26390600 6.49405100 8.36432200
H 7.39727000 8.17447800 7.84099800
C 4.99639900 8.60777600 8.26433000
C 4.62008600 6.37705600 7.60359800
C 3.64016200 8.79459900 8.48950900
H 5.73188500 9.38566300 8.42247900
C 3.24263200 6.50493400 7.80878500
H 5.06828000 5.45493500 7.25589800
C 2.75337500 7.74853000 8.26357700
H 3.29270200 9.76021400 8.83750400
C 2.38917400 5.40100100 7.54668500
H 1.69009600 7.87898300 8.43189000
C 1.66781600 4.45304800 7.29964000

Bowl **2**$^{6+}$ - Cis-in$_3$

E(RB3LYP) = -3715.669612

Number of Imaginary Frequencies = 2

Cartesian Coordinates:

C -4.25585200 36.81298000 -28.80422400

C -2.44029500 38.31267300 -29.71826800

C -4.30551500 39.29155700 -28.32466400

C -5.66523200 36.64971400 -25.19714800

C -5.69624700 38.04612400 -24.92879900

C -5.29014400 38.95306900 -25.92594200

C -4.83551700 38.48221600 -27.15333400

C -4.80661400 37.10628000 -27.41866700

C -5.23075400 36.19517700 -26.45680100

C -1.40378600 41.48783900 -26.98897200

C -0.35815600 40.95149100 -27.79092800

C -0.64989300 39.93769500 -28.72261600
C -1.94692100 39.44735400 -28.83593100
C -2.97908900 39.98297400 -28.05383800
C -2.71626900 41.00375600 -27.14562700
C -0.24516300 35.22436400 -28.90432000
C -0.58055900 36.50426800 -29.38426100
C -1.89664800 36.95060200 -29.31890700
C -2.90225800 36.12119800 -28.80392100
C -2.59509300 34.84327100 -28.34734400
C -1.26488900 34.38392400 -28.37786100
C -3.97622200 38.22475800 -29.42414100
C 2.30795300 34.55042100 -28.97004300
C 1.12199300 34.81528600 -28.93382000
C -0.78752600 31.99533200 -27.35580400
C -0.96907600 33.09200400 -27.84812600
C -6.31158200 34.81148000 -23.41374700
C -6.03765900 35.68887700 -24.20949400
C -6.41050100 39.08041300 -22.60579300
C -6.10389700 38.55966600 -23.66074600
C -0.99717000 43.30330300 -25.11363300
C -1.14871900 42.48292300 -25.99798200
C 2.16494200 41.70409300 -27.57532300
C 0.99175800 41.39508600 -27.65436000
C 6.25193500 42.61770900 -27.28666000
N 5.72635100 41.54941100 -26.64353200
C 4.41135700 41.25648600 -26.73356100
C 3.54536200 42.04775500 -27.49212200
C 4.08416600 43.16414900 -28.16390000
C 5.44310600 43.44017100 -28.05644900
C 6.43461300 33.78977500 -29.06382300
C 5.64626000 33.29171100 -30.09035100
C 4.27492800 33.52261000 -30.07896400
C 3.70268100 34.26430700 -29.02518800
C 4.54887800 34.73992100 -28.02047100
N 5.87710700 34.49927300 -28.05551600
C -0.53484400 46.00860700 -21.93684100
C -0.61692500 46.51166600 -23.22674900
C -0.76439100 45.63775000 -24.29862400
C -0.83496400 44.24973800 -24.06076600
C -0.74974500 43.80477100 -22.73931500
N -0.60239400 44.67506000 -21.71755400
C -7.30036400 41.02168300 -18.99147500
N -6.03172400 40.66959000 -19.30353700
C -5.74641400 40.03778500 -20.46211600
C -6.75081200 39.72408000 -21.38076800
C -8.07644300 40.08290500 -21.06148900
C -8.34139700 40.73533700 -19.86207800
C -7.08281700 31.70813200 -20.69501100
C -8.15551100 32.29586000 -21.34835800
C -7.92939200 33.33019500 -22.25038400
C -6.61080800 33.76671500 -22.49204300
C -5.57209900 33.13341000 -21.80471000
N -5.82017300 32.13231300 -20.93353800
C -0.24453600 28.27419400 -25.49031800
N -0.35291400 29.41201400 -24.76616400
C -0.52405400 30.60938200 -25.36584300
C -0.59427200 30.71689500 -26.75698300
C -0.48092200 29.53656500 -27.51971000
C -0.30791100 28.31627400 -26.87510600
C -4.91705600 40.93488700 -18.32679600
C -0.45991400 44.13652900 -20.32004200
C -3.78872200 41.75846000 -18.90708900
C -3.96486900 43.12217300 -19.19595100
C -2.90194800 43.88538900 -19.67236300
C -1.63963300 43.30187300 -19.87164500
C -1.46537800 41.94347300 -19.58796500
C -2.53237400 41.17734800 -19.10772200
C 6.73779600 34.98192300 -26.91901800
C 6.57176400 39.22957100 -26.10992300
C 7.12791700 38.75884700 -27.31128300

C 7.15977200 37.39440400 -27.58716500
C 6.63607500 36.47004900 -26.66758700
C 6.07505800 36.93941300 -25.47547600
C 6.04233900 38.31008700 -25.19868800
C 6.60461900 40.70145000 -25.76295100
C -4.67455100 31.52546500 -20.16727500
C -1.41056500 29.95711700 -22.53779200
C -2.66774000 29.33010800 -22.56269000
C -3.72900200 29.84675400 -21.82355100
C -3.55724700 31.00253700 -21.04277000
C -2.30764900 31.63091300 -21.02523200
C -1.24223000 31.11269200 -21.76811900
C -0.22817500 29.35848700 -23.26755300
H -5.33137100 40.01616800 -25.71112900
H -5.22755400 35.12776500 -26.65298000
H -4.98061200 36.23625400 -29.39179700
H 0.15526300 39.54461500 -29.33504000
H -3.50579900 41.43359700 -26.53751300
H -5.05496600 40.01857500 -28.66060400
H 0.20719300 37.13045000 -29.79081800
H -3.36319500 34.18596400 -27.95251200
H -2.22679800 38.52076900 -30.77374800
H -4.56600600 38.38763800 -30.32696400
H 7.31388600 42.78657700 -27.16429400
H 4.06898400 40.38366900 -26.19251600
H 3.43692800 43.79700700 -28.76109000
H 5.88644000 44.28862200 -28.56419000
H 7.50524700 33.63782000 -29.02039200
H 6.11509000 32.72912500 -30.88899600
H 3.64321300 33.14144300 -30.87376900
H 4.17976900 35.31676500 -27.18187100
H -0.41339200 46.64014300 -21.06637700
H -0.56304800 47.58313200 -23.37855800
H -0.82734400 46.01410000 -25.31375900
H -0.79589400 42.75479800 -22.48089300
H -7.44897500 41.52632400 -18.04561000
H -4.70736800 39.79195700 -20.63896900
H -8.87852200 39.85178600 -21.75384700
H -9.34927400 41.02788400 -19.59211700
H -7.19996300 30.90480200 -19.97947400
H -9.15738000 31.93705000 -21.14404800
H -8.75646000 33.80169300 -22.76969400
H -4.53605700 33.41542700 -21.94081100
H -0.10690400 27.35470300 -24.93613200
H -0.60344300 31.47166400 -24.71637000
H -0.53188200 29.58784000 -28.60174100
H -0.22100300 27.39237700 -27.43464000
H 0.46341600 43.55397100 -20.30444800
H -0.32219700 45.00552900 -19.67515900
H -4.92859200 43.59691600 -19.03123800
H -3.05374500 44.94310600 -19.87138400
H -0.49313200 41.47792400 -19.72545100
H -2.37412700 40.12704800 -18.87714400
H 6.43586700 34.41201500 -26.03774600
H 7.75873300 34.69500300 -27.17333100
H 7.55633100 39.45626600 -28.02635500
H 7.61361600 37.04953500 -28.51258900
H 5.67397900 36.23803500 -24.74869000
H 5.61547200 38.65596700 -24.26085700
H -2.81623900 28.42294100 -23.14283800
H -4.68627500 29.33234300 -21.83881100
H -2.15276900 32.51922900 -20.41844200
H -0.27457200 31.60557200 -21.72912300
H -0.10147400 28.30497200 -23.01534000
H 0.69696200 29.88022500 -23.01369100
H -5.10848600 30.72718400 -19.56405800
H -4.31093700 32.30124100 -19.49002300
H -4.55983500 39.95845800 -17.99391800
H -5.37686700 41.43575300 -17.47408700
H 6.27204100 40.87104200 -24.73653400

H 7.61166200 41.10799800 -25.86391300

Bowl $\mathbf{2}^{6+}$ - Cis-in$_2$Trans

E(RB3LYP) = -3715.671739

Number of Imaginary Frequencies = 0

Cartesian Coordinates:

C 0.18492400 -1.70598000 3.94353900

C -0.96893800 -0.37782000 5.75610200

C 3.05426200 -0.55964400 1.60640400

C 3.80993800 0.17714800 2.56021700

C 3.37767100 0.22588800 3.89921700

C 2.20295500 -0.41671700 4.27628400

C 1.45055700 -1.12928000 3.33281700

C 1.87822200 -1.21809300 2.01223400

C 1.16257200 3.20963200 6.86367500

C 1.80327400 2.01758700 6.47893700

C 1.04862500 0.90302600 6.12833800

C -0.35185500 0.94768200 6.17599500

C -1.00418500 2.11240300 6.56797900

C -0.25925700 3.25872400 6.90784600

C -3.48320000 0.30406400 2.88376300

C -2.93242800 0.33410900 4.18078400

C -1.73997400 -0.32958400 4.44753900

C -1.09463400 -1.05969700 3.43916100

C -1.62763100 -1.11231100 2.15498800

C -2.81624000 -0.42202400 1.85561500

C 0.25814900 -1.30516600 5.45559300

H 3.97267600 0.77848600 4.61934200

H 1.31668800 -1.78343900 1.27514600

H 0.14659200 -2.79270000 3.80034000

H -3.45589500 0.88614100 4.95514500

H -1.13926500 -1.67512800 1.36608000

H -1.60220900 -0.77368300 6.55939500

H 0.26103500 -2.18372500 6.10177300

H 2.88820400 1.99096200 6.45963300

H -2.08721500 2.16059500 6.62137300

C 1.55322000 -0.44650800 5.65026300

H 2.23722100 -0.88510200 6.38700400

C -3.30906500 -0.44808000 0.51691300

C -3.65195800 -0.48971300 -0.64876300

C -4.04455200 -0.54382400 -2.01728900

C -4.73061600 -0.59004600 -4.70314700

C -5.09917600 -1.36716200 -2.46177300

C -3.37092100 0.23188900 -2.96460200

N -3.72004600 0.19828200 -4.26847900

C -5.43393600 -1.38551500 -3.81144900

H -5.63932700 -1.98134700 -1.74963800

H -2.55135200 0.88612100 -2.69598600

H -6.23664200 -2.01130800 -4.18305500

H -4.95152400 -0.55944700 -5.76200900

C 3.44010400 -0.63276400 0.23413800

C 3.68067000 -0.73168800 -0.95343900

C 3.93424500 -0.86778000 -2.34896800

C 4.32560100 -1.08592800 -5.08559200

C 3.28170300 -0.03065500 -3.25728100

C 4.81502400 -1.84290700 -2.86007400

C 5.00309800 -1.94571600 -4.23421800

N 3.48371000 -0.15133000 -4.58675800

H 2.59229900 0.73994900 -2.93704600

H 5.33553100 -2.50768500 -2.17948000

H 5.67021400 -2.68785300 -4.65646700

H 4.43621800 -1.11776400 -6.16159200

H -1.47634800 -1.00109800 -6.10082700

C -0.88298400 -0.16555700 -5.73904500

C 0.66770000 1.98354400 -4.86347000

C 0.50619500 -0.23521000 -5.80776600

C -1.51409500 0.98419000 -5.23497200

C -0.72731900 2.05357300 -4.79433000
C 1.29584100 0.84293000 -5.37408900
H 0.97445600 -1.12389500 -6.22246700
H -1.19826000 2.95510200 -4.41167800
H 1.26200000 2.83131600 -4.53266500
C 2.80093000 0.81120600 -5.52222400
H 3.23641300 1.79352200 -5.32810100
H 3.08954700 0.50477300 -6.52838100
C -3.02133300 1.11009000 -5.24258500
H -3.33159300 2.12644500 -4.99069000
H -3.42955200 0.86544400 -6.22399400
C 4.98704400 0.89352300 2.18880500
C 5.98005200 1.54925900 1.93960600
C 7.14564100 2.31922100 1.65972500
C 8.40396900 1.72141600 1.44277600
C 7.06011900 3.71278500 1.60020700
C 9.50980900 2.52366700 1.18266600
H 8.50115600 0.64207600 1.48334600
N 8.14942200 4.46762200 1.34133600
H 6.12734200 4.23980500 1.75530900
C 9.36119400 3.90149900 1.13373900
H 10.49012300 2.09295300 1.01679100
C 7.99663000 5.95938500 1.20753500
H 10.18469700 4.57354300 0.93040300
C 7.32661800 6.62259800 2.39042700
H 7.42984000 6.13103800 0.28976800
H 9.00446100 6.34948000 1.05994400
C 7.96453300 6.69190700 3.64059100
C 6.08867600 7.25172800 2.22328500
H 8.93393800 6.22311900 3.78819400
C 7.37761900 7.38786500 4.69455900
C 5.50049000 7.95239500 3.28094800
H 5.58498000 7.21573800 1.26111100
C 6.14070900 8.03188300 4.52190200
H 7.89812800 7.44904100 5.64658200
H 4.54768300 8.45167600 3.12646900
C 5.53984000 8.87095600 5.62751800
N 5.04219800 8.07085000 6.80223100
H 4.68680400 9.44793400 5.26357400
H 6.27367000 9.56913400 6.03199300
C 5.43243700 8.40926400 8.05303000
C 4.16484300 7.06663900 6.59131400
C 4.93504700 7.72586600 9.15223100
H 6.13660400 9.22647400 8.13936200
C 3.62795400 6.34131900 7.65804000
H 3.90416200 6.85588500 5.56205800
C 4.02759300 6.68867900 8.96469300
H 5.26442700 8.01224700 10.14410000
C 2.71939400 5.27416800 7.40121600
H 3.62861200 6.14307100 9.81274900
C 1.96434200 4.34845800 7.17541300
C -0.94679500 4.44862800 7.28663600
C -1.58177200 5.43962500 7.59264000
C -2.32692600 6.58965300 7.97710600
C -2.06471900 7.28038900 9.17845700
C -3.35921700 7.05980400 7.15966200
C -2.83526800 8.38715800 9.51714800
H -1.26913400 6.93982900 9.83191200
N -4.09225900 8.13699000 7.51419700
H -3.61295200 6.58596200 6.22010100
C -3.85110000 8.80078700 8.66901400
H -2.66115400 8.93321100 10.43678500
C -5.14453100 8.65959100 6.57683800
H -4.48241400 9.65361900 8.88162500
C -6.06571600 7.59420300 6.02655200
H -4.61742100 9.17578500 5.77101900
H -5.70437300 9.40472000 7.14503500
C -6.78037700 6.73598800 6.87307300
C -6.26486200 7.50646800 4.64236500
H -6.64217000 6.78516000 7.95004500

C -7.67207200 5.80378700 6.34102700

C -7.17825900 6.59410300 4.11226200

H -5.71600200 8.16267100 3.97228200

C -7.88830100 5.73274200 4.95802800

H -8.21188900 5.13881500 7.00978200

H -7.32679900 6.55773800 3.03636300

C -8.93371900 4.77863500 4.41906000

N -8.45979500 3.98138200 3.24119900

H -9.24757400 4.06513200 5.18392800

H -9.82050100 5.31826100 4.08143300

C -9.15261300 4.02292800 2.07930300

C -7.35683900 3.21377800 3.37342800

C -8.73564600 3.27233500 0.98926400

H -10.02686300 4.66025600 2.05520100

C -6.88337300 2.44024800 2.30882500

H -6.86176200 3.23554400 4.33591000

C -7.59987700 2.47785500 1.09305500

H -9.30457100 3.32146700 0.06841000

C -5.70655900 1.66376800 2.47635800

H -7.25886100 1.88867000 0.24890000

C -4.68652000 1.01884900 2.63048500

Bowl **2^{6+}** - Cis-in$_2$Cis-out

E(RB3LYP) = -3715.673264

Number of Imaginary Frequencies = 0

Cartesian Coordinates:

C 0.10587600 -1.65620600 4.00979400
C -1.03527500 -0.30775000 5.81588300
C 3.01723700 -0.60576200 1.68083000
C 3.77916700 0.13169200 2.62930200
C 3.33787000 0.20932100 3.96385000
C 2.14734700 -0.40379000 4.34090000
C 1.38866400 -1.11597300 3.40206000
C 1.82534800 -1.23457600 2.08674400
C 1.12611500 3.28687700 6.83759000
C 1.75745300 2.08125400 6.48059000
C 0.99481800 0.96091800 6.16559000
C -0.40492600 1.01244000 6.23023800
C -1.04734400 2.18511700 6.61122700
C -0.29536300 3.34071100 6.90379600
C -3.48457800 0.47169500 2.91285000
C -2.95978900 0.46347500 4.22072100
C -1.79294400 -0.23947900 4.49983100
C -1.15243100 -0.97696600 3.49411200
C -1.66633600 -1.00065400 2.20108900
C -2.82489900 -0.26611500 1.88854600
C 0.18196400 -1.24868000 5.52013900
H 3.93886200 0.76037200 4.68007000
H 1.25882800 -1.80061700 1.35408100
H 0.03861100 -2.74226200 3.87251500
H -3.47804100 1.02512300 4.99149500
H -1.18345200 -1.57138600 1.41443900
H -1.67965400 -0.69547200 6.61403500
H 0.17745400 -2.12403300 6.17061200
H 2.84191600 2.05012300 6.45071600
H -2.12915900 2.23882300 6.67871300
C 1.48643900 -0.40157800 5.70973500
H 2.15912900 -0.83651800 6.45908100
C -3.29767300 -0.26562400 0.54234200
C -3.62603900 -0.29251200 -0.62796300
C -4.00642200 -0.34025800 -2.00023700
C -4.66405200 -0.38729100 -4.69357300
C -5.08188700 -1.13331300 -2.45033300
C -3.29881900 0.40667000 -2.94581100
N -3.63431100 0.37266100 -4.25337700
C -5.40218300 -1.15249100 -3.80346000
H -5.64877200 -1.72455200 -1.73957800
H -2.46169500 1.03707800 -2.67408500
H -6.22004000 -1.75587800 -4.17909300
H -4.87131800 -0.35923500 -5.75533000
C 3.41548100 -0.70625200 0.31398300
C 3.67343400 -0.82106500 -0.86845000
C 3.95167000 -0.96445400 -2.25844200
C 4.40435200 -1.18180800 -4.98541100
C 3.32527500 -0.12211200 -3.18033800
C 4.83546900 -1.94642000 -2.75048900
C 5.05367500 -2.04943400 -4.12023700
N 3.55864500 -0.24112300 -4.50460700
H 2.63264200 0.65159500 -2.87492500
H 5.33533900 -2.61581600 -2.05906300
H 5.72374700 -2.79691000 -4.52815100
H 4.54104200 -1.21155700 -6.05847300
H -1.41811400 -0.93662800 -6.02610000
C -0.79857300 -0.11460300 -5.67733700
C 0.82239100 1.99681600 -4.83687900
C 0.58763700 -0.23539800 -5.73327300
C -1.39134700 1.06792400 -5.20317600
C -0.56976300 2.11762600 -4.77936400
C 1.41251600 0.82327500 -5.31735200
H 1.02648400 -1.14940300 -6.12455400
H -1.01112800 3.04276400 -4.41862600
H 1.44406800 2.83040900 -4.52094600
C 2.91603500 0.73517200 -5.45410300
H 3.38559100 1.70311900 -5.26664700
H 3.20029900 0.40827800 -6.45508300
C -2.89271000 1.25080100 -5.22602500
H -3.16595200 2.28001000 -4.98354300
H -3.30078200 1.01607300 -6.20991900
C 4.96981200 0.82228400 2.25254800
C 5.97245500 1.45723100 1.98869100
C 7.14500700 2.20735600 1.68495200
C 8.39649600 1.59219800 1.47762300

C 7.07203500 3.59951600 1.58792700
C 9.50791400 2.37679400 1.18859000
H 8.48437100 0.51353100 1.54724100
N 8.16635400 4.33675500 1.30156000
H 6.14529600 4.13945000 1.73425800
C 9.37152800 3.75402500 1.10196400
H 10.48323600 1.93268000 1.02869000
C 8.02638500 5.82540100 1.12687000
H 10.19975500 4.41237100 0.87429600
C 7.35459600 6.52415700 2.28786600
H 7.46761300 5.97720500 0.20060200
H 9.03838000 6.20337800 0.97651700
C 7.98680800 6.61992900 3.53920100
C 6.12243700 7.15831300 2.09830400
H 8.95094300 6.14587400 3.70382000
C 7.40077700 7.34806700 4.57158000
C 5.53503200 7.89105100 3.13453200
H 5.62273800 7.10128200 1.13508500
C 6.17034200 7.99781700 4.37598300
H 7.91725700 7.43066600 5.52409500
H 4.58674800 8.39352500 2.96299700
C 5.57273800 8.87117200 5.45640900
N 5.05854700 8.10750100 6.64820800
H 4.72910800 9.44913200 5.07272200
H 6.31252000 9.57039600 5.84796200
C 5.44254200 8.47687600 7.89220000
C 4.17179600 7.10726400 6.45843600
C 4.92690400 7.83225600 9.00629700
H 6.15614200 9.28759700 7.96128100
C 3.61706800 6.41974800 7.54092500
H 3.91755600 6.86952900 5.43344200
C 4.00825500 6.80128400 8.84063500
H 5.25092100 8.14355700 9.99239600
C 2.69914900 5.35502500 7.30842200
H 3.59444400 6.28603100 9.70051500
C 1.93638000 4.42912200 7.11107400
C -0.98361900 4.54295900 7.22743600
C -1.63943000 5.53953900 7.46599000
C -2.41836100 6.69830200 7.72533300
C -1.86172600 7.99504100 7.74291800
C -3.79016400 6.56976400 7.96656400
C -2.68044000 9.09025800 7.99258900
H -0.80114800 8.12779600 7.55939300
N -4.55971800 7.65080100 8.21160300
H -4.28666500 5.60794800 7.96755400
C -4.03411500 8.89789300 8.22831800
H -2.28139200 10.09753500 8.00790800
C -6.01753600 7.45326300 8.53256400
H -4.71654700 9.71188900 8.43448900
C -6.77271500 6.67405600 7.47913000
H -6.43470200 8.45321400 8.65597000
H -6.05705900 6.94826600 9.50027200
C -7.27757100 5.40418600 7.77680600
C -7.03530900 7.23559000 6.21822800
H -7.09325700 4.95830200 8.75065400
C -8.03953600 4.70578900 6.83464000
C -7.79369700 6.54001200 5.28005400
H -6.66512100 8.22803700 5.97438700
C -8.30676600 5.26726500 5.58186500
H -8.43702000 3.72683300 7.08914200
H -8.00347800 7.00009000 4.31802800
C -9.20216400 4.53863000 4.60410400
N -8.49676200 4.08608300 3.35398300
H -9.63405000 3.64392300 5.05741400
H -10.02112600 5.17725200 4.27081400
C -8.99025000 4.43435700 2.14277700
C -7.41485000 3.28749500 3.46581000
C -8.38742300 3.97111900 0.98207200
H -9.86274100 5.07453000 2.13711200
C -6.76601100 2.78489200 2.33305900
H -7.08069700 3.05751300 4.46932600
C -7.27508900 3.14158600 1.06601400
H -8.79835800 4.26420700 0.02315100
C -5.62912900 1.94859500 2.49147100
H -6.79472300 2.76839000 0.16818300
C -4.64801000 1.24609500 2.64689600

Bowl **2**$^{6+}$ - Cis-out$_3$

E(RB3LYP) = -3715.679748

Number of Imaginary Frequencies = 0

Cartesian Coordinates:

C 0.12497800 -1.79434500 4.12071600
C -0.98872700 -0.52634800 5.99943400
C 2.94914600 -0.58778000 1.75395600
C 3.74434200 0.09571200 2.72000600
C 3.33940500 0.10281000 4.06952400
C 2.15821200 -0.52585900 4.44769900
C 1.37646500 -1.19920000 3.49756000
C 1.77312200 -1.24574800 2.16561600
C 1.14083100 3.07624000 7.05964000
C 1.78239600 1.88614300 6.66337500
C 1.03047000 0.76332800 6.33520400
C -0.36863300 0.79303300 6.42987700
C -1.02055500 1.94615400 6.85294000
C -0.28118900 3.10655000 7.15586000
C -3.56122600 0.25775000 3.20610900
C -2.98519600 0.23707600 4.49185500
C -1.79119700 -0.44111800 4.71165700
C -1.16966400 -1.14057500 3.66678000
C -1.73710600 -1.15965100 2.39742100
C -2.92694200 -0.44926700 2.14290800
C 0.23401800 -1.43806700 5.64234000
H 3.96204600 0.61429800 4.79653200
H 1.18685900 -1.77602000 1.42187600
H 0.08813400 -2.87679100 3.94756600
H -3.48795500 0.76739400 5.29433100
H -1.27406700 -1.70638700 1.58229500
H -1.60053400 -0.94531200 6.80756000
H 0.25651800 -2.33566400 6.26127400
H 2.86630800 1.87304300 6.60938900
H -2.10124800 1.98037400 6.94556600
C 1.53074900 -0.57968300 5.83049500
H 2.22608900 -1.02672300 6.55129700
C -3.44690700 -0.44459400 0.81866100
C -3.80730500 -0.47194200 -0.34315500
C -4.19534700 -0.50511700 -1.70929600
C -4.86249200 -0.64632800 -4.40029800
C -5.11534200 0.41301300 -2.25923100
C -3.64658800 -1.47125600 -2.55894300
N -3.98402000 -1.52526000 -3.86458900
C -5.44015400 0.33572500 -3.60853700

H -5.55845600 1.17496400 -1.62789000
H -2.93520400 -2.20830300 -2.20917900
H -6.13939900 1.03075300 -4.05807400
H -5.08143100 -0.75564700 -5.45442900
C 3.29650600 -0.61446300 0.37480100
C 3.50768100 -0.66761100 -0.82224100
C 3.71961400 -0.74440300 -2.22466700
C 4.04150200 -0.97971000 -4.97191900
C 2.99929600 -1.67722500 -2.97789800
C 4.63186200 0.09094800 -2.90401700
C 4.78277600 -0.03245100 -4.28034700
N 3.17106600 -1.77938300 -4.31258900
H 2.28271300 -2.35078400 -2.52587400
H 5.20533000 0.82544500 -2.35002400
H 5.47233700 0.59865800 -4.82841200
H 4.12180000 -1.12261100 -6.04159500
H -1.72920900 -4.51940800 -3.71915500
C -1.19239100 -3.70721200 -4.20214100
C 0.21046200 -1.65064600 -5.46405500
C 0.20279700 -3.75982000 -4.28383300
C -1.89444000 -2.62958100 -4.75161100
C -1.17854100 -1.59731500 -5.38144100
C 0.91471600 -2.73629000 -4.91698700
H 0.73123200 -4.61045300 -3.86150200
H -1.70582900 -0.75733600 -5.82639300
H 0.74461000 -0.85189200 -5.97206900
C 2.41645300 -2.84332000 -5.06324400
H 2.72106300 -2.74826800 -6.10628500
H 2.78038900 -3.80367800 -4.69181900
C -3.40695600 -2.62037900 -4.72044200
H -3.82279500 -2.47286000 -5.71792900
H -3.79868200 -3.56072200 -4.32683300
C 4.93493600 0.78654500 2.36151500
C 5.95276400 1.41367300 2.13539700
C 7.14004000 2.15453800 1.89242600
C 7.56727100 2.49551800 0.59141700
C 7.92692600 2.57195800 2.97113400
C 8.73833500 3.22479800 0.42154400
H 6.97830400 2.18894600 -0.26556300
N 9.06185200 3.27518200 2.77595700
H 7.66301100 2.34944500 3.99703800
C 9.47836100 3.60607900 1.53148000
H 9.08848300 3.50349400 -0.56538900
C 9.90975200 3.63747600 3.96596600
H 10.39990200 4.16856000 1.45860300
C 9.16183200 4.39267000 5.04148000
H 10.73897500 4.22805600 3.57474900
H 10.31494200 2.70053100 4.35404600
C 8.95780700 3.80261100 6.29289600
C 8.72484600 5.71073300 4.82737100
H 9.29805600 2.78726200 6.47857900
C 8.33303800 4.51734900 7.31984500
C 8.10283200 6.42239600 5.84992400
H 8.88883400 6.19174700 3.86663200
C 7.90396400 5.83158000 7.10915800
H 8.19527000 4.04826700 8.29047200
H 7.79018500 7.44773100 5.67042700
C 7.30063900 6.61826200 8.25176900
N 5.85159700 6.96666600 8.04590600
H 7.81980200 7.56653000 8.39650000
H 7.35501800 6.05715300 9.18699300
C 5.45715200 8.26013700 8.09837300
C 4.96115400 5.96950700 7.86187500
C 4.11588200 8.58860000 7.96339300
H 6.23110200 9.00107100 8.25033200
C 3.59523500 6.23624500 7.72137300
H 5.35685000 4.96231700 7.83140000
C 3.17509200 7.58233100 7.77734800
H 3.82289000 9.63102900 8.00628800
C 2.69015300 5.15982600 7.52172900
H 2.12329200 7.82281100 7.67124900
C 1.93850000 4.22208100 7.33216500
C -0.98460200 4.28518100 7.52910900
C -1.66127800 5.25537300 7.81381900
C -2.47011500 6.38012000 8.12700100
C -1.96935200 7.69954000 8.11547600
C -3.81761800 6.19328400 8.45203600
C -2.81682600 8.75900400 8.41756700
H -0.92904500 7.87787700 7.86746000
N -4.61609100 7.24091000 8.74501100
H -4.27226700 5.21133500 8.48227500
C -4.14476000 8.50928500 8.73269900
H -2.46054700 9.78233400 8.41159600
C -6.04393600 6.97818100 9.14056000
H -4.84840800 9.29365000 8.97899400
C -6.82874400 6.21801300 8.09452000
H -6.48649400 7.95587900 9.33478400
H -6.00915300 6.42934800 10.08385900
C -7.27835400 4.92163600 8.36466100
C -7.16788300 6.81810200 6.87018900
H -7.03395400 4.44548200 9.31054400
C -8.06091600 4.23428700 7.43131200
C -7.94613600 6.13326000 5.94051400
H -6.84159900 7.83097500 6.64832800
C -8.40314400 4.83322300 6.21461800
H -8.41500000 3.23366700 7.66489200
H -8.21407100 6.62228800 5.00767900
C -9.31510700 4.11016000 5.24830400
N -8.64483500 3.71859900 3.95843100
H -10.16499600 4.73356400 4.96735400
H -9.70239600 3.18886700 5.68831300
C -9.18540500 4.10239700 2.77852000
C -7.54341600 2.94002300 4.00348700
C -8.61380600 3.69646200 1.58132600
H -10.06884800 4.72539400 2.82586100
C -6.92260600 2.49634500 2.83098000
H -7.17062700 2.68014100 4.98584900
C -7.48287700 2.88862200 1.59657800
H -9.06258100 4.01720000 0.64860700
C -5.76032900 1.68440200 2.91623400
H -7.02590200 2.56073200 0.66971700
C -4.75510000 1.00491600 3.00668200

Bowl **2**$^{6+}$ - Cis-out$_2$Trans

E(RB3LYP) = -3715.678511

Number of Imaginary Frequencies = 0

Cartesian Coordinates:

C 0.07217100 -1.74944800 4.01073400

C -1.06982900 -0.40365800 5.81669300

C 2.95912700 -0.60954100 1.68723800
C 3.73251300 0.09407300 2.65655700
C 3.29939800 0.12602300 3.99680300
C 2.10884900 -0.49309600 4.36113200
C 1.34450200 -1.17939200 3.40630600
C 1.77166300 -1.25520200 2.08508200
C 1.11324700 3.14873900 6.93575800
C 1.73720400 1.94130400 6.56570500
C 0.96725200 0.84070300 6.20656800
C -0.43314500 0.91180100 6.23702200
C -1.06989600 2.08911800 6.61798400
C -0.30976100 3.22332900 6.96140000
C -3.57587900 0.29508700 2.94098400
C -3.02097400 0.32917500 4.23587100
C -1.83741700 -0.34899800 4.50662000
C -1.20301900 -1.09709300 3.50364800
C -1.74399200 -1.16138100 2.22447000
C -2.92450100 -0.45599700 1.91900700
C 0.14251200 -1.35256500 5.52419000
H 3.90571500 0.65247400 4.72692600
H 1.20064300 -1.79831000 1.33873600
H 0.03093500 -2.83573200 3.86519300
H -3.53562900 0.89417500 5.00661900
H -1.26853300 -1.74256600 1.44098000
H -1.71232000 -0.78655600 6.61906300
H 0.12365900 -2.23223600 6.16851700
H 2.82165300 1.89594800 6.56035800
H -2.15237300 2.15532500 6.66023400
C 1.45120300 -0.51827100 5.73111000
H 2.12257600 -0.97032600 6.47110200
C -3.41425400 -0.49299600 0.58406800
C -3.74602900 -0.54973300 -0.58512800
C -4.10195800 -0.60633200 -1.95898300
C -4.70498900 -0.78545500 -4.66203800
C -5.02740400 0.28825300 -2.53791300
C -3.51486600 -1.56928800 -2.78632600
N -3.82183200 -1.64200300 -4.09851100
C -5.31985700 0.19230800 -3.89335200
H -5.49942300 1.04641800 -1.92299000
H -2.79720200 -2.28881500 -2.41367300
H -6.02216200 0.86886700 -4.36578100
H -4.89733500 -0.90891700 -5.71976100
C 3.34238300 -0.66905000 0.31883700
C 3.59124300 -0.75179900 -0.86923700
C 3.86189700 -0.85601600 -2.25960500
C 4.30941900 -1.14073400 -4.98361900
C 3.13893300 -1.76629100 -3.03791600
C 4.84145900 -0.06860300 -2.90123700
C 5.05533300 -0.21649600 -4.26663300
N 3.37206400 -1.89270500 -4.36099300
H 2.37221500 -2.40286900 -2.61548200
H 5.41830300 0.64664500 -2.32591800
H 5.79809700 0.37701400 -4.78648800
H 4.43896300 -1.30225900 -6.04562800
H -1.54806700 -4.61179200 -3.86164300
C -1.00320800 -3.80187300 -4.33939200
C 0.42105900 -1.75302300 -5.58994600
C 0.39370500 -3.85003400 -4.38846400
C -1.69645200 -2.73405600 -4.91843500
C -0.96979100 -1.70500700 -5.54118600
C 1.11666300 -2.83095500 -5.01671700
H 0.91478400 -4.69560000 -3.94722800
H -1.48946700 -0.87317000 -6.00968100
H 0.96353700 -0.95798000 -6.09506700
C 2.62013000 -2.93981300 -5.13909800
H 2.93852000 -2.82865300 -6.17645200
H 2.97475900 -3.90758900 -4.77765500
C -3.20920100 -2.73590500 -4.93110800
H -3.59626300 -2.59612300 -5.94118200
H -3.60489300 -3.67785100 -4.54533100

C 4.92356300 0.79065100 2.30984000
C 5.93392000 1.43430300 2.09707600
C 7.10503100 2.20802600 1.87829700
C 7.49789500 2.64606000 0.59546000
C 7.90610400 2.56741400 2.96709000
C 8.64826000 3.41321800 0.45377800
H 6.89817700 2.38585700 -0.26951100
N 9.02000600 3.31061700 2.79907300
H 7.66977700 2.26721700 3.97990700
C 9.40187000 3.73674900 1.57303500
H 8.97155000 3.76680000 -0.51823200
C 9.86972700 3.62621300 3.99938900
H 10.30723800 4.32718300 1.52141500
C 9.13464300 4.40327400 5.06932000
H 10.72764200 4.18447400 3.62253400
H 10.23126800 2.67092700 4.38537200
C 8.91218400 3.82311000 6.32209600
C 8.72241600 5.72766400 4.84563000
H 9.23366500 2.80308000 6.51536400
C 8.29201900 4.55342000 7.34091000
C 8.10406600 6.45440900 5.85976100
H 8.90131700 6.20170800 3.88395300
C 7.88487000 5.87292200 7.11994500
H 8.13950800 4.09157400 8.31278200
H 7.80865200 7.48360600 5.67332900
C 7.27856400 6.67381700 8.25059500
N 5.82909800 7.01796300 8.03570600
H 7.79619200 7.62453200 8.38388100
H 7.33012200 6.12489100 9.19328200
C 5.42841400 8.30891800 8.10026100
C 4.94456400 6.01926300 7.83286100
C 4.08654100 8.63301100 7.95951600
H 6.19779100 9.05149200 8.26666500
C 3.57876900 6.28171300 7.68406500
H 5.34527100 5.01443000 7.79254400
C 3.15156300 7.62509200 7.75397700
H 3.78865300 9.67357400 8.01278300
C 2.67916500 5.20580700 7.45944100
H 2.09911100 7.86177700 7.64308500
C 1.92565300 4.27587300 7.24120000
C -0.98041900 4.42395600 7.33420400
C -1.59948600 5.42501500 7.63996400
C -2.32607100 6.58540000 8.02827300
C -2.03960900 7.27887400 9.22255100
C -3.36538200 7.06190900 7.22360000
C -2.79444300 8.39456000 9.56725000
H -1.23815100 6.93327600 9.86614600
N -4.08332100 8.14704800 7.58438900
H -3.63778200 6.58606000 6.29033600
C -3.81903000 8.81365100 8.73240400
H -2.60178900 8.94313700 10.48167800
C -5.14784300 8.67303700 6.66286300
H -4.43964200 9.67303100 8.95020600
C -6.08007200 7.60936600 6.12834800
H -4.63157400 9.18685600 5.84856700
H -5.69632000 9.42011800 7.23955100
C -6.78248200 6.75362900 6.98761600
C -6.30015400 7.51865200 4.74763000
H -6.62862700 6.80567200 8.06234900
C -7.68119500 5.81949900 6.47125800
C -7.22145600 6.60498300 4.23347300
H -5.76137900 8.17306400 4.06766000
C -7.91749500 5.74461000 5.09166400
H -8.21047500 5.15586400 7.14972900
H -7.38665000 6.56691500 3.16013300
C -8.97009300 4.78765600 4.57188600
N -8.52011300 3.99745200 3.38004000
H -9.26261400 4.06926500 5.34070000
H -9.86718600 5.32450300 4.25763100
C -9.23561000 4.04633000 2.23220700

C -7.41602900 3.22743100 3.48611400

C -8.84153000 3.30117700 1.13012300

H -10.10942100 4.68465300 2.22887400

C -6.96559800 2.45768800 2.40867300

H -6.90233500 3.24309100 4.43887100

C -7.70578900 2.50367100 1.20753100

H -9.42838500 3.35607800 0.22092700

C -5.78964500 1.67464900 2.55170600

H -7.38393900 1.91784100 0.35379700

C -4.77344900 1.02023100 2.69116200

Bowl **2**$^{6+}$ - Cis-out$_2$Cisin

E(RB3LYP) = -3715.676484

Number of Imaginary Frequencies = 0

Cartesian Coordinates:

C 0.13139700 -1.80867000 4.03548400

C -1.00093500 -0.49973300 5.87377200

C 2.98448400 -0.61296900 1.69938100

C 3.76154300 0.08476400 2.66994300

C 3.34363800 0.09268800 4.01541400

C 2.16322700 -0.54316500 4.38404000

C 1.39476500 -1.22299700 3.42779600

C 1.80824300 -1.27633300 2.10120600

C 1.15640500 3.06771900 6.98811300

C 1.79019400 1.86634900 6.61241000

C 1.02904600 0.75845000 6.25651400

C -0.37108900 0.81420500 6.30568800

C -1.01651500 1.98349500 6.69587200

C -0.26619300 3.12743500 7.02434800

C -3.51262100 0.27373600 3.02667900

C -2.95572800 0.27320200 4.31880800

C -1.77672300 -0.42166900 4.57016900

C -1.14984100 -1.15029500 3.54947300

C -1.69917200 -1.18607300 2.27278200

C -2.87630100 -0.46482100 1.98868000

C 0.21522200 -1.43419800 5.55441400

H 3.95263500 0.61520000 4.74614000

H 1.23361300 -1.81385900 1.35359200

H 0.09262300 -2.89269600 3.87369600

H -3.46135100 0.82692600 5.10344600

H -1.23267100 -1.75434600 1.47447400

H -1.63527200 -0.90010200 6.67408800

H 0.21099300 -2.32357500 6.18554700

H 2.87502800 1.83305700 6.59735000

H -2.09906000 2.03873600 6.74699500

C 1.51952000 -0.59135200 5.76002500

H 2.20144200 -1.04756800 6.48773400

C -3.38177200 -0.47348100 0.65880100

C -3.73927400 -0.50541100 -0.50369600

C -4.13147400 -0.54175900 -1.86822600

C -4.81350000 -0.69005500 -4.55433500

C -5.09594200 0.33932900 -2.40233000

C -3.54582800 -1.47452700 -2.73072100

N -3.89182000 -1.53309800 -4.03361900

C -5.42775700 0.25888900 -3.74983500

H -5.56864200 1.07390400 -1.75964700

H -2.79967300 -2.18208600 -2.39271400

H -6.16119200 0.92535300 -4.18804900

H -5.03707300 -0.80152600 -5.60718800

C 3.35111800 -0.64879300 0.32566000

C 3.58332500 -0.71309700 -0.86692000

C 3.83103300 -0.80013700 -2.26266900

C 4.23244900 -1.05507400 -4.99686600

C 3.09427900 -1.70118100 -3.03877800

C 4.80036400 -0.00627200 -2.91185100

C 4.99097000 -0.13923000 -4.28224400

N 3.30496200 -1.81333800 -4.36699600

H 2.33470900 -2.34205200 -2.61010200
H 5.38788100 0.70184600 -2.33844600
H 5.72556500 0.45933200 -4.80787500
H 4.34426400 -1.20483000 -6.06259800
H -1.63611900 -4.50653300 -3.86575700
C -1.08723400 -3.69425300 -4.33487500
C 0.34674400 -1.63932700 -5.56454800
C 0.30902000 -3.75211400 -4.39106300
C -1.77504300 -2.61326300 -4.89590500
C -1.04342800 -1.58102800 -5.50748000
C 1.03669300 -2.73041800 -5.00966700
H 0.82594600 -4.60703300 -3.96309500
H -1.55964900 -0.73874100 -5.96087200
H 0.89275300 -0.84188200 -6.06200900
C 2.53857000 -2.85118000 -5.14305000
H 2.85040600 -2.73959400 -6.18237800
H 2.88742800 -3.82329900 -4.78773800
C -3.28742400 -2.60306700 -4.90332200
H -3.67533000 -2.42703200 -5.90736200
H -3.68915100 -3.55415800 -4.54698400
C 4.93929700 0.80128600 2.31884800
C 5.93764100 1.46196600 2.10167800
C 7.09418200 2.25553400 1.87698200
C 7.46618600 2.70988000 0.59358500
C 7.90130300 2.61865300 2.96003800
C 8.60256800 3.49648600 0.44599600
H 6.86145000 2.44678600 -0.26703600
N 9.00184000 3.38019900 2.78618100
H 7.68029600 2.30678700 3.97273500
C 9.36338800 3.82239800 1.55967200
H 8.90966700 3.86309200 -0.52644800
C 9.86140700 3.69666200 3.97924900
H 10.25908900 4.42697000 1.50290200
C 9.12849500 4.45018800 5.06744300
H 10.70557800 4.27241900 3.59800700
H 10.24293900 2.74295200 4.34961000
C 8.93233500 3.85237100 6.31632000
C 8.69308700 5.77072300 4.86487100
H 9.27159600 2.83519400 6.49346400
C 8.31623900 4.56164100 7.35219900
C 8.07800400 6.47632400 5.89594400
H 8.85221700 6.25865300 3.90659500
C 7.88645200 5.87730200 7.15244700
H 8.18468100 4.08660500 8.32078300
H 7.76353800 7.50291900 5.72624600
C 7.28842200 6.65686000 8.30175900
N 5.83002500 6.98130000 8.11955600
H 7.79447900 7.61393700 8.43450200
H 7.36656900 6.09933700 9.23767800
C 5.40681500 8.25957800 8.25395800
C 4.96068100 5.97631900 7.88520800
C 4.05665000 8.56376300 8.15412200
H 6.16523000 9.00779000 8.44344600
C 3.58722300 6.21867200 7.77662200
H 5.37928900 4.98250700 7.79050000
C 3.13659100 7.54861600 7.91879400
H 3.74041000 9.59449500 8.26319600
C 2.70207000 5.13670300 7.52597100
H 2.07768900 7.76930900 7.84098100
C 1.95867900 4.20124300 7.29688100
C -0.95686400 4.32791300 7.36677900
C -1.61427400 5.31830100 7.62157200
C -2.39813300 6.47014500 7.91902500
C -2.59644800 6.92097200 9.24010300
C -3.00578800 7.18274700 6.88203900
C -3.38140400 8.04541400 9.47154500
H -2.13805700 6.38726400 10.06543300
N -3.76158200 8.27278100 7.13519100
H -2.89514500 6.89605100 5.84400200
C -3.95688200 8.71193700 8.40037100

H -3.55415700 8.41359200 10.47592300

C -4.33712600 9.05228600 5.98295100

H -4.57141900 9.59460600 8.51987200

C -5.16950900 8.22233200 5.03075200

H -3.49101000 9.51003700 5.46595000

H -4.93165100 9.84948500 6.43101400

C -6.40470500 7.68263500 5.42778300

C -4.74671300 8.05198200 3.70845900

H -6.75919500 7.81591200 6.44654600

C -7.20092600 6.99669600 4.51392300

C -5.54676500 7.36413000 2.79069300

H -3.79838200 8.47022300 3.38198900

C -6.78085400 6.83553800 3.18276800

H -8.16325400 6.60620100 4.83400900

H -5.20954200 7.25773200 1.76313300

C -7.67581600 6.17145000 2.16026900

N -7.84931800 4.69005900 2.36974800

H -8.68060200 6.59511200 2.18362700

H -7.27846800 6.29570900 1.15057400

C -9.09548400 4.16448400 2.41753300

C -6.75521600 3.90278100 2.44719900

C -9.27542600 2.79573100 2.54737000

H -9.92076500 4.86093800 2.34714700

C -6.87164000 2.51671600 2.57931000

H -5.79356100 4.39789900 2.40320100

C -8.16629400 1.96037500 2.62644300

H -10.28243700 2.39767300 2.58577000

C -5.69857400 1.71428600 2.68074000

H -8.28800600 0.88754300 2.72833400

C -4.69989100 1.03028100 2.79388400

Bowl **2**$^{6+}$ - Mixed (TransCis-outCis-in)

E(RB3LYP) = -3715.675229

Number of Imaginary Frequencies = 1

Cartesian Coordinates:

C 1.22530000 -1.84522900 2.98808200

C -0.86026800 -1.48217600 4.36365200

C 0.20166100 0.45842200 3.14600900

C 1.97093500 -0.66193300 -0.62815200

C 1.39109000 0.63779500 -0.53878000

C 0.82499600 1.06090400 0.67992300

C 0.80540000 0.20458400 1.77516200

C 1.36920700 -1.07685200 1.68549000

C 1.95899000 -1.50466500 0.50114600

C -3.51200900 1.42105100 2.58658400

C -4.10996400 0.31512600 3.25602500

C -3.28971300 -0.64316400 3.87908300

C -1.90476000 -0.52739300 3.80941000

C -1.31523900 0.55480400 3.14144600

C -2.10768300 1.53262300 2.55061300

C -1.69257200 -5.02482400 3.04001300

C -1.83852400 -3.83054100 3.76905400

C -0.85832500 -2.84637700 3.69673600

C 0.29176100 -3.04367300 2.91967700

C 0.46325200 -4.22494300 2.20484500

C -0.52775900 -5.22526800 2.24701100

C 0.51112300 -0.84365800 3.95938100

C -3.69419300 -6.74211900 3.17448100

C -2.74193500 -5.99076000 3.09343100

C -0.15106800 -7.41983100 0.82707500

C -0.34580300 -6.42130100 1.49332200

C 3.07227300 -1.61767600 -2.82577000

C 2.56057800 -1.13864900 -1.83122900

C 1.22454600 2.31700000 -2.56534200

C 1.33877800 1.51757700 -1.65521800

C -4.90177800 3.26944800 1.31756000

C -4.29612200 2.40621800 1.92429900

C -6.71722900 -0.09774400 3.34093200

C -5.52529400 0.13681200 3.29216900

C -10.80306300 -1.05389900 3.45107800

N -9.97102300 -1.63973600 2.55902700

C -8.65673100 -1.33224800 2.52160400

C -8.10975000 -0.39421500 3.40077800

C -8.96980200 0.22192600 4.33289100

C -10.31836100 -0.11768900 4.35216800

C -7.11608200 -9.16041700 3.41662700

C -6.07409600 -9.39979200 4.30012600

C -4.92468000 -8.61928100 4.23686000

C -4.83307300 -7.59252100 3.27505300

C -5.91502700 -7.40148800 2.41243200

N -7.02001800 -8.17276700 2.49683100

C -6.84036800 6.30617100 -0.83122900

C -7.57390000 5.18996800 -0.45538100

C -6.95889500 4.16962100 0.26075400

C -5.59165400 4.27808600 0.59368900

C -4.90561600 5.42590100 0.18308700

N -5.52978500 6.40219700 -0.50821200

C 0.72421100 5.17443000 -5.59572700

N 0.51708700 5.48683900 -4.29524900

C 0.67669500 4.56808600 -3.31987100

C 1.06808800 3.25837300 -3.61716800

C 1.28643800 2.92425100 -4.97083800

C 1.10887800 3.89044700 -5.95418200

C 4.80942600 -3.42052900 -6.19224700

C 4.42930800 -2.08892500 -6.27642800

C 3.85351000 -1.46575700 -5.17572500

C 3.66186200 -2.19411600 -3.98197000

C 4.06889700 -3.53216700 -3.95770200

N 4.62231200 -4.11275800 -5.04401500

C 0.62686400 -10.85440800 -1.45348400

N 0.72225000 -9.61868400 -1.99820600

C 0.46402600 -8.51150400 -1.27045900

C 0.09344800 -8.60272900 0.07463000

C -0.01500800 -9.88680000 0.64766300

C 0.25590800 -11.00970100 -0.12664100

C 0.16120200 6.89820900 -3.91301000

C -4.76184800 7.64582900 -0.86845300

C -1.11578900 7.00703200 -3.10964500

C -1.06902700 7.46235100 -1.78826100

C -2.24846700 7.64374600 -1.05887500

C -3.49069800 7.37094100 -1.64069200

C -3.53726800 6.90279300 -2.96473200

C -2.36293200 6.72378900 -3.69150800

C -8.14353100 -7.94621500 1.52081300

C -9.84883600 -3.96782100 1.58637400

C -10.02247000 -4.83181800 2.68061400

C -9.45640800 -6.10396600 2.67169700

C -8.70554600 -6.54208100 1.56798600

C -8.52966300 -5.68033500 0.48038600

C -9.09629400 -4.40153500 0.49009100

C -10.52187000 -2.61340700 1.55193800

C 5.07846900 -5.53833100 -4.95213600

C 2.06839800 -8.41836700 -3.77466800

C 3.34266400 -8.43053600 -3.19102000

C 4.28907700 -7.46909100 -3.54673500

C 3.99194300 -6.49825300 -4.51366400

C 2.72053400 -6.48938000 -5.10092900

C 1.76385100 -7.43282100 -4.72282600

C 1.04733700 -9.48828000 -3.46084900

H 0.40034000 2.05811500 0.73797200

H 2.41378800 -2.48700100 0.42219400

H 2.21068100 -2.15189600 3.35999300

H -3.75902700 -1.47164500 4.39952800

H -1.66623600 2.38439500 2.04299900

H 0.64423700 1.35209800 3.60227800

H -2.72780400 -3.69685400 4.37681300

H 1.35403600 -4.39816700 1.60911700
H -0.97065700 -1.58294100 5.45019900
H 1.12624300 -0.63114700 4.83456800
H -11.84267800 -1.35250700 3.41657700
H -8.05714300 -1.84683500 1.78147500
H -8.57390300 0.95340800 5.02878900
H -11.00272400 0.33584800 5.05933500
H -8.03208200 -9.73661400 3.41973100
H -6.17489900 -10.19435300 5.02996000
H -4.10137500 -8.79108000 4.92160200
H -5.91417400 -6.63189500 1.65126700
H -7.26866500 7.13215600 -1.38355100
H -8.62122600 5.13304700 -0.72760300
H -7.51923100 3.29189700 0.56363100
H -3.85649100 5.57875200 0.40125700
H 0.57903900 5.96744900 -6.31751000
H 0.49064600 4.89779300 -2.30567500
H 1.58764300 1.91651300 -5.23445700
H 1.26602800 3.66130100 -7.00157900
H 5.26142100 -3.95547200 -7.01726700
H 4.58721300 -1.55465500 -7.20584800
H 3.55096900 -0.42584600 -5.22560800
H 3.95653200 -4.15330800 -3.07816900
H 0.84919500 -11.69117200 -2.10286800
H 0.56044600 -7.56092100 -1.77878200
H -0.30137800 -9.98867700 1.68869000
H 0.18862500 -12.00839400 0.28825200
H -5.44886900 8.26601400 -1.44558800
H -4.54791800 8.16048400 0.07081000
H -0.11352400 7.69368900 -1.32519800
H -2.19377000 8.01437300 -0.03877100
H -4.49336500 6.69623200 -3.43874200
H -2.42168400 6.38191300 -4.72158200
H -7.74801400 -8.17963700 0.53019800
H -8.90596600 -8.68736400 1.76341500
H -10.61746400 -4.52125500 3.53553100
H -9.61914200 -6.76372300 3.51976400
H -7.96223900 -6.00528500 -0.38776600
H -8.96015900 -3.75055900 -0.36950700
H 3.60345800 -9.18638600 -2.45460000
H 5.26971200 -7.48728200 -3.07881500
H 2.46325700 -5.74881900 -5.85337300
H 0.78002500 -7.40843200 -5.18373800
H 1.40421500 -10.47125500 -3.77350300
H 0.10333900 -9.29183400 -3.97427600
H 5.92176800 -5.55732300 -4.25833800
H 5.45454700 -5.80085500 -5.94287500
H 0.08690400 7.45330700 -4.84907900
H 1.01211200 7.29140400 -3.35264400
H -10.41246600 -2.14301200 0.57262500
H -11.58751200 -2.69697900 1.76929100

1. Schrödinger Release 2022-2: MacroModel, Schrödinger, LLC, New York, NY, 2021.

2. Frisch, M.J. et al. Gaussian 16, Revision C.01, Gaussian, Inc., Wallingford CT, 2016.

3. D.A. Case, I.Y. Ben-Shalom, S.R. Brozell, D.S. Cerutti, T.E. Cheatham, III, V.W.D. Cruzeiro, T.A. Darden, R.E. Duke, D. Ghoreishi, M.K. Gilson, H. Gohlke, A.W. Goetz, D. Greene, R Harris, N. Homeyer, Y. Huang, S. Izadi, A. Kovalenko, T. Kurtzman, T.S. Lee, S. LeGrand, P. Li, C. Lin, J. Liu, T. Luchko, R. Luo, D.J. Mermelstein, K.M. Merz, Y. Miao, G. Monard, C. Nguyen, H. Nguyen, I. Omelyan, A. Onufriev, F. Pan, R. Qi, D.R. Roe, A. Roitberg, C. Sagui, S. Schott-Verdugo, J. Shen, C.L. Simmerling, J. Smith, R. Salomon Ferrer, J. Swails, R.C. Walker, J. Wang, H. Wei, R.M. Wolf, X. Wu, L. Xiao, D.M. York and P.A. Kollman (2018), AMBER 2018, University of California, San Francisco.
4. B.H. Besler, K.M. Merz and P.A. Kollman, J. Comput. Chem., 1990, 11, 431-439.
5. J. Wang; R. M. Wolf; J. W. Caldwell; P. A. Kollamn; D. A. Case. Development and testing of a general Amber force field. J. Comput. Chem., 2004, 25, 1157–1174.
6. MATLAB. (2010). *version R2021a*. Natick, Massachusetts: The MathWorks Inc.
7. Ditchfield, R. Self-consistent perturbation theory of diamagnetism. Mol. Phys. 1974, 27, 789-807.
8. Wolinski, K.; Hinton, J. F.; Pulay, P. Efficient Implementation of the Gauge-Independent Atomic Orbital Method for NMR Chemical Shift Calculations. J. Am. Chem. Soc. 1990, 112, 8251-8260.
9. http://gohom.win/ManualHom/Schrodinger/Schrodinger_2015-2_docs/macromodel/macromodel_user_manual.pdf

X-Ray Studies

The single crystal X-ray diffraction studies were carried out on a Bruker Kappa Photon III CPAD diffractometer equipped with Mo K_{α} radiation (λ = 0.71073 Å). A 0.452 x 0.232 x 0.228 mm piece of a light-yellow block was mounted on a MiTeGen MicroMount with Paratone 24EX oil. Data were collected in a nitrogen gas stream at 100(2) K using ϕ and ϖ scans. Crystal-to-detector distance was 50 mm using variable exposure time (5s-30s) depending on θ with a scan width of 1.0°. Data collection was 99.6% complete to 20.868° in θ (1.0Å). A total of 41774 reflections were collected covering the indices, -25<=h<=25, -25<=k<=25, -14<=l<=15. 5758 reflections were found to be symmetry independent, with a R_{int} of 0.1244. Indexing and unit cell refinement indicated a primitive, trigonal lattice. The space group was found to be *P*31c. The data were integrated using the Bruker SAINT software program and scaled using the SADABS software program. Solution by dual-space method (SHELXT) produced a complete phasing model for refinement.

All nonhydrogen atoms were refined anisotropically by full-matrix least-squares (SHELXL-2014). All hydrogen atoms were placed using a riding model. Their positions were constrained relative to their parent atom using the appropriate HFIX command in SHELXL-2014. Due to unmodelable solvent disorder, Platon SQUEEZE was used to remove the electron density from the lattice due to the disordered solvent contribution. Solvent appeared to be Acetonitrile. One void was found with approximately 148 electrons, which matches well to approx. 6 acetonitrile molecules in the void. TFAs were added at chemically reasonable locations via quasi-rigid bodies (TFAs built of restraints) to balance the charge of the molecule and match NMR data. The pocket outside of cage was completely diffuse with no real TFA starting positions. Two TFA positions refined at 2/3 occupancy and the remaining two at 1/3 occupancy for an overall total of 6 per cage. Crystallographic data are summarized in Table 1.

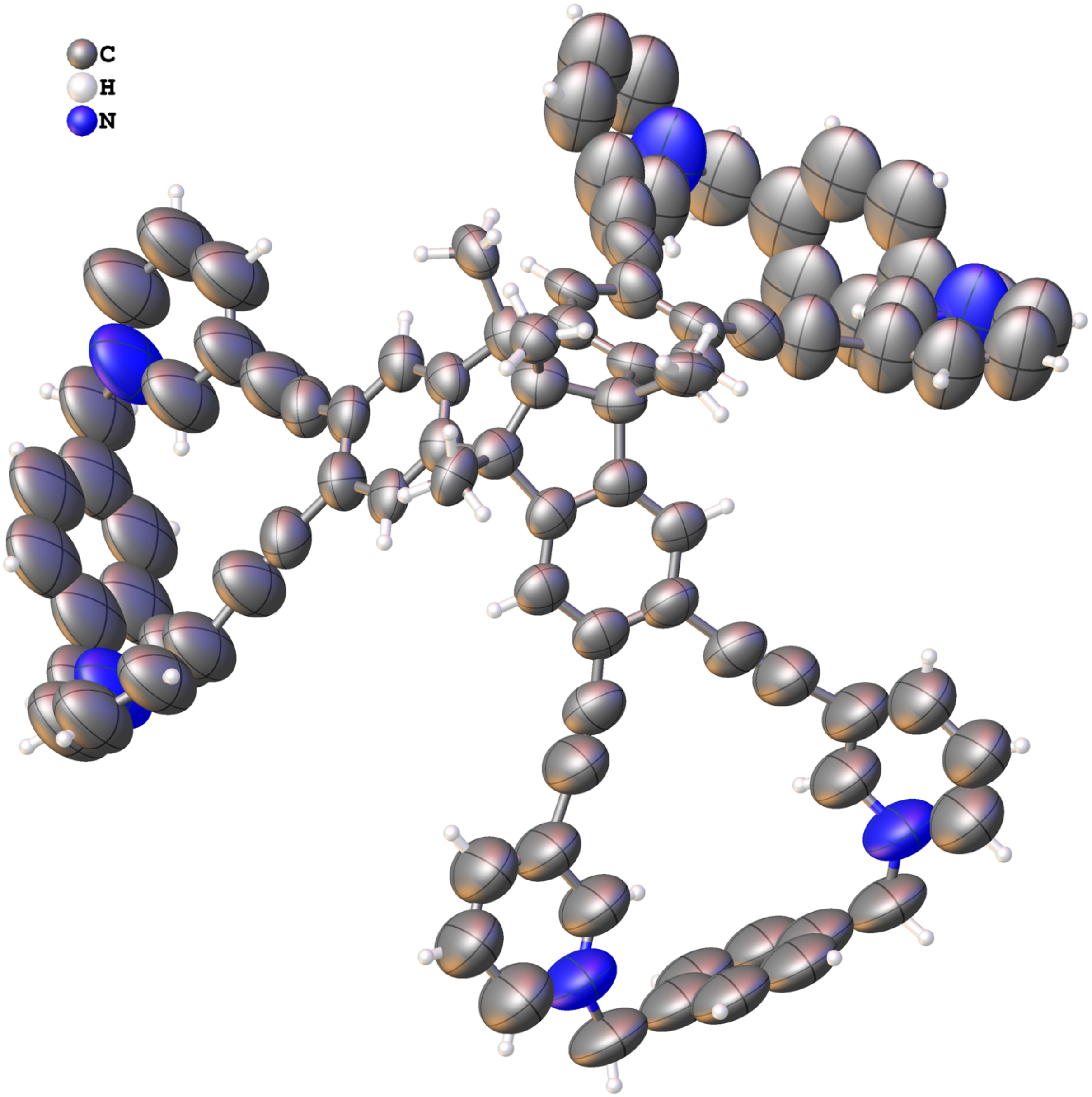
C
H
N

Table 1. Crystal data and structure refinement for **1**$(CF_3CO_2)_6$.

Report date	2023-08-15	
Identification code	PK20230327	
Empirical formula	C110 H73 F17 N9 O12	
Molecular formula	C92 H64 N6, 6(C2 F3 O2), 3[C2H3N]	
Formula weight	2035.77	
Temperature	100.0 K	
Wavelength	0.71073 Å	
Crystal system	Trigonal	
Space group	P31c	
Unit cell dimensions	a = 25.2070(12) Å	α= 90°.
	b = 25.2070(12) Å	β= 90°.
	c = 14.9840(10) Å	γ = 120°.
Volume	8245.2(10) $Å^3$	
Z	2	
Density (calculated)	0.820 Mg/m^3	
Absorption coefficient	0.067 mm^{-1}	
F(000)	2090	
Crystal size	0.452 x 0.232 x 0.228 mm^3	
Crystal color, habit	yellow block	
Theta range for data collection	1.866 to 20.868°.	
Index ranges	-25<=h<=25, -25<=k<=25, -14<=l<=15	
Reflections collected	41774	
Independent reflections	5758 [R(int) = 0.1244, R(sigma) = 0.0702]	
Completeness to theta = 20.868°	99.6 %	

Absorption correction	Semi-empirical from equivalents
Max. and min. transmission	0.0847 and 0.0626
Refinement method	Full-matrix least-squares on F^2
Data / restraints / parameters	5758 / 760 / 334
Goodness-of-fit on F^2	1.396
Final R indices [I>2sigma(I)]	R1 = 0.1611, wR2 = 0.3690
R indices (all data)	R1 = 0.2652, wR2 = 0.4500
Absolute structure parameter	0.9(6)
Extinction coefficient	n/a
Largest diff. peak and hole	0.603 and -0.389 e.Å^{-3}

Table 2. Atomic coordinates (x 10^4) and equivalent isotropic displacement parameters ($Å^2$x 10^3) for **1**($(CF_3CO_2)_6$. U(eq) is defined as one third of the trace of the orthogonalized U^{ij} tensor.

	x	y	z	U(eq)
C(5)	5961(9)	2492(6)	5670(9)	90(5)
C(10)	5473(6)	2058(8)	5167(12)	102(6)
C(9)	5538(7)	1626(5)	4676(10)	103(6)
C(8)	6091(9)	1629(6)	4686(10)	116(8)
C(7)	6579(7)	2064(8)	5188(12)	92(5)
C(6)	6514(7)	2496(5)	5680(9)	89(5)
C(2)	6667	3333	6630(20)	109(8)
C(4)	5436(9)	2715(11)	6966(13)	103(5)
C(3)	6014(9)	3009(11)	6244(12)	95(4)
C(20)	4024(10)	489(11)	3189(19)	207(8)
C(22)	3489(12)	438(11)	3537(19)	218(9)
C(23)	2923(10)	-43(14)	3270(20)	241(10)
C(24)	2892(9)	-471(12)	2660(20)	262(9)
N(2)	3427(10)	-420(11)	2312(18)	243(8)
C(21)	3994(9)	60(12)	2577(19)	221(9)
C(19)	4676(12)	934(9)	3736(13)	190(9)
C(18)	5101(12)	1225(10)	4092(16)	129(5)
C(32)	3368(11)	-708(15)	1410(20)	242(7)
C(1)	6667	3333	7700(19)	90(8)
C(13)	6486(12)	475(10)	3316(17)	207(8)

C(15)	6758(11)	178(13)	3733(15)	218(9)
C(16)	6704(13)	-352(13)	3363(18)	241(10)
C(17)	6379(14)	-586(12)	2574(19)	262(9)
N(1)	6107(11)	-289(11)	2157(15)	243(8)
C(14)	6160(11)	241(10)	2527(16)	221(9)
C(12)	6307(8)	875(10)	3806(13)	190(9)
C(11)	6177(11)	1167(11)	4164(16)	129(5)
C(25)	5702(10)	-585(9)	1359(11)	242(7)
C(26)	5095(8)	-613(7)	1368(6)	242(7)
C(27)	4971(10)	-266(7)	771(9)	242(7)
C(28)	4407(11)	-293(9)	793(15)	242(7)
C(29)	3966(9)	-667(11)	1410(18)	242(7)
C(30)	4089(9)	-1014(9)	2006(14)	242(7)
C(31)	4653(10)	-988(7)	1985(8)	242(7)
F(7A)	6450(30)	3390(20)	3970(40)	253(7)
F(9A)	6300(30)	2470(20)	3920(40)	253(7)
F(8A)	6580(30)	3030(20)	2730(40)	253(7)
O(5A)	5480(40)	3000(40)	2740(70)	253(7)
O(6A)	5200(30)	2130(30)	3560(60)	253(7)
C(5A)	5550(20)	2640(30)	3220(50)	253(7)
C(6A)	6240(20)	2890(20)	3460(30)	253(7)
F(1A)	4230(16)	2651(14)	3710(30)	253(7)
F(2A)	4631(16)	2091(15)	4090(30)	253(7)
F(3A)	3810(20)	1740(20)	3230(30)	253(7)
O(1A)	3934(19)	1985(19)	5540(30)	253(7)

O(2A)	3140(17)	1640(20)	4490(30)	253(7)
C(1A)	3686(17)	1874(16)	4780(30)	253(7)
C(2A)	4097(16)	2081(15)	3950(30)	253(7)
F(5A)	6360(20)	2080(20)	940(30)	328(18)
F(6A)	5694(17)	1715(19)	1920(30)	295(16)
F(4A)	6640(20)	2231(18)	2290(30)	295(16)
O(3A)	5799(19)	750(18)	2030(20)	243(16)
O(4A)	6670(20)	1050(20)	1460(40)	282(19)
C(3A)	6280(20)	1191(17)	1720(30)	283(18)
C(4A)	6251(17)	1788(16)	1730(30)	299(15)
F(12A)	3830(40)	1360(30)	40(40)	290(30)
F(11A)	4640(30)	2150(40)	560(40)	300(20)
F(10A)	3740(30)	2020(30)	870(40)	290(20)
O(8A)	4250(50)	2260(50)	-1360(40)	280(30)
O(7A)	3860(20)	2660(20)	-370(40)	200(20)
C(7A)	4070(30)	2330(30)	-600(30)	270(20)
C(8A)	4080(30)	1960(30)	230(30)	290(20)

Table 3. Bond lengths [Å] and angles [°] for **1**$(CF_3CO_2)_6$.

C(5)-C(10)	1.3900	C(22)-H(22)	0.9500
C(5)-C(6)	1.3900	C(22)-C(23)	1.3900
C(5)-C(3)	1.51(2)	C(23)-H(23)	0.9500
C(10)-H(10)	0.9500	C(23)-C(24)	1.3900
C(10)-C(9)	1.3900	C(24)-H(24)	0.9500
C(9)-C(8)	1.3900	C(24)-N(2)	1.3900
C(9)-C(18)	1.37(3)	N(2)-C(21)	1.3900
C(8)-C(7)	1.3900	N(2)-C(32)	1.500(18)
C(8)-C(11)	1.50(3)	C(21)-H(21)	0.9500
C(7)-H(7)	0.9500	C(19)-C(18)	1.09(2)
C(7)-C(6)	1.3900	C(32)-H(32A)	0.9900
C(6)-C(3)#1	1.50(3)	C(32)-H(32B)	0.9900
C(2)-C(3)#2	1.54(2)	C(32)-C(29)	1.460(17)
C(2)-C(3)#1	1.54(2)	C(1)-H(1A)	0.9800
C(2)-C(3)	1.54(2)	C(1)-H(1B)	0.9800
C(2)-C(1)	1.61(5)	C(1)-H(1C)	0.9800
C(4)-H(4A)	0.9800	C(13)-C(15)	1.3900
C(4)-H(4B)	0.9800	C(13)-C(14)	1.3900
C(4)-H(4C)	0.9800	C(13)-C(12)	1.49(3)
C(4)-C(3)	1.66(3)	C(15)-H(15)	0.9500
C(20)-C(22)	1.3900	C(15)-C(16)	1.3900
C(20)-C(21)	1.3900	C(16)-H(16)	0.9500
C(20)-C(19)	1.67(3)	C(16)-C(17)	1.3900

C(17)-H(17)	0.9500	F(1A)-C(2A)	1.352(19)
C(17)-N(1)	1.3900	F(2A)-C(2A)	1.350(19)
N(1)-C(14)	1.3900	F(3A)-C(2A)	1.331(19)
N(1)-C(25)	1.505(15)	O(1A)-C(1A)	1.27(2)
C(14)-H(14)	0.9500	O(2A)-C(1A)	1.27(2)
C(12)-C(11)	1.09(2)	C(1A)-C(2A)	1.54(3)
C(25)-H(25A)	0.9900	F(5A)-C(4A)	1.345(19)
C(25)-H(25B)	0.9900	F(6A)-C(4A)	1.352(19)
C(25)-C(26)	1.496(17)	F(4A)-C(4A)	1.349(19)
C(26)-C(27)	1.3900	O(3A)-C(3A)	1.26(2)
C(26)-C(31)	1.3900	O(4A)-C(3A)	1.26(2)
C(27)-H(27)	0.9500	C(3A)-C(4A)	1.54(2)
C(27)-C(28)	1.3900	F(12A)-C(8A)	1.341(19)
C(28)-H(28)	0.9500	F(11A)-C(8A)	1.335(19)
C(28)-C(29)	1.3900	F(10A)-C(8A)	1.345(19)
C(29)-C(30)	1.3900	O(8A)-C(7A)	1.26(2)
C(30)-H(30)	0.9500	O(7A)-C(7A)	1.26(2)
C(30)-C(31)	1.3900	C(7A)-C(8A)	1.55(3)
C(31)-H(31)	0.9500		
F(7A)-C(6A)	1.331(19)	C(10)-C(5)-C(6)	120.0
F(9A)-C(6A)	1.326(19)	C(10)-C(5)-C(3)	130.5(15)
F(8A)-C(6A)	1.332(19)	C(6)-C(5)-C(3)	109.5(15)
O(5A)-C(5A)	1.24(2)	C(5)-C(10)-H(10)	120.0
O(6A)-C(5A)	1.24(2)	C(5)-C(10)-C(9)	120.0
C(5A)-C(6A)	1.56(3)	C(9)-C(10)-H(10)	120.0

C(10)-C(9)-C(8)	120.0	C(5)-C(3)-C(4)	107.9(16)
C(18)-C(9)-C(10)	123.3(17)	C(6)#2-C(3)-C(5)	110.6(19)
C(18)-C(9)-C(8)	116.4(17)	C(6)#2-C(3)-C(2)	104.9(19)
C(9)-C(8)-C(11)	121.4(17)	C(6)#2-C(3)-C(4)	109.7(18)
C(7)-C(8)-C(9)	120.0	C(2)-C(3)-C(4)	117.4(16)
C(7)-C(8)-C(11)	118.6(17)	C(22)-C(20)-C(21)	120.0
C(8)-C(7)-H(7)	120.0	C(22)-C(20)-C(19)	117.5(11)
C(6)-C(7)-C(8)	120.0	C(21)-C(20)-C(19)	120.0(12)
C(6)-C(7)-H(7)	120.0	C(20)-C(22)-H(22)	120.0
C(5)-C(6)-C(3)#1	112.5(15)	C(20)-C(22)-C(23)	120.0
C(7)-C(6)-C(5)	120.0	C(23)-C(22)-H(22)	120.0
C(7)-C(6)-C(3)#1	127.5(15)	C(22)-C(23)-H(23)	120.0
C(3)#1-C(2)-C(3)#2	106.9(14)	C(24)-C(23)-C(22)	120.0
C(3)#1-C(2)-C(3)	106.9(14)	C(24)-C(23)-H(23)	120.0
C(3)-C(2)-C(3)#2	106.9(14)	C(23)-C(24)-H(24)	120.0
C(3)#2-C(2)-C(1)	112.0(13)	C(23)-C(24)-N(2)	120.0
C(3)#1-C(2)-C(1)	112.0(13)	N(2)-C(24)-H(24)	120.0
C(3)-C(2)-C(1)	112.0(13)	C(24)-N(2)-C(21)	120.0
H(4A)-C(4)-H(4B)	109.5	C(24)-N(2)-C(32)	115.6(13)
H(4A)-C(4)-H(4C)	109.5	C(21)-N(2)-C(32)	119.2(14)
H(4B)-C(4)-H(4C)	109.5	C(20)-C(21)-H(21)	120.0
C(3)-C(4)-H(4A)	109.5	N(2)-C(21)-C(20)	120.0
C(3)-C(4)-H(4B)	109.5	N(2)-C(21)-H(21)	120.0
C(3)-C(4)-H(4C)	109.5	C(18)-C(19)-C(20)	180.0(19)
C(5)-C(3)-C(2)	106.3(17)	C(19)-C(18)-C(9)	164.7(18)

N(2)-C(32)-H(32A)	112.4	C(14)-N(1)-C(17)	120.0
N(2)-C(32)-H(32B)	112.4	C(14)-N(1)-C(25)	120.6(13)
H(32A)-C(32)-H(32B)	109.9	C(13)-C(14)-H(14)	120.0
C(29)-C(32)-N(2)	96.9(16)	N(1)-C(14)-C(13)	120.0
C(29)-C(32)-H(32A)	112.4	N(1)-C(14)-H(14)	120.0
C(29)-C(32)-H(32B)	112.4	C(11)-C(12)-C(13)	180.0(4)
C(2)-C(1)-H(1A)	109.5	C(12)-C(11)-C(8)	171.9(18)
C(2)-C(1)-H(1B)	109.5	N(1)-C(25)-H(25A)	108.2
C(2)-C(1)-H(1C)	109.5	N(1)-C(25)-H(25B)	108.2
H(1A)-C(1)-H(1B)	109.5	H(25A)-C(25)-H(25B)	107.4
H(1A)-C(1)-H(1C)	109.5	C(26)-C(25)-N(1)	116.2(13)
H(1B)-C(1)-H(1C)	109.5	C(26)-C(25)-H(25A)	108.2
C(15)-C(13)-C(14)	120.0	C(26)-C(25)-H(25B)	108.2
C(15)-C(13)-C(12)	122.7(14)	C(27)-C(26)-C(25)	120.6(11)
C(14)-C(13)-C(12)	113.1(13)	C(27)-C(26)-C(31)	120.0
C(13)-C(15)-H(15)	120.0	C(31)-C(26)-C(25)	119.4(11)
C(13)-C(15)-C(16)	120.0	C(26)-C(27)-H(27)	120.0
C(16)-C(15)-H(15)	120.0	C(28)-C(27)-C(26)	120.0
C(15)-C(16)-H(16)	120.0	C(28)-C(27)-H(27)	120.0
C(17)-C(16)-C(15)	120.0	C(27)-C(28)-H(28)	120.0
C(17)-C(16)-H(16)	120.0	C(27)-C(28)-C(29)	120.0
C(16)-C(17)-H(17)	120.0	C(29)-C(28)-H(28)	120.0
C(16)-C(17)-N(1)	120.0	C(28)-C(29)-C(32)	119.9(15)
N(1)-C(17)-H(17)	120.0	C(28)-C(29)-C(30)	120.0
C(17)-N(1)-C(25)	119.1(13)	C(30)-C(29)-C(32)	120.1(15)

C(29)-C(30)-H(30)	120.0
C(31)-C(30)-C(29)	120.0
C(31)-C(30)-H(30)	120.0
C(26)-C(31)-H(31)	120.0
C(30)-C(31)-C(26)	120.0
C(30)-C(31)-H(31)	120.0
O(5A)-C(5A)-O(6A)	134(5)
O(5A)-C(5A)-C(6A)	113(4)
O(6A)-C(5A)-C(6A)	113(4)
F(7A)-C(6A)-F(8A)	108(3)
F(7A)-C(6A)-C(5A)	111(3)
F(9A)-C(6A)-F(7A)	107(3)
F(9A)-C(6A)-F(8A)	109(3)
F(9A)-C(6A)-C(5A)	111(3)
F(8A)-C(6A)-C(5A)	110(3)
O(1A)-C(1A)-C(2A)	119(3)
O(2A)-C(1A)-O(1A)	135(4)
O(2A)-C(1A)-C(2A)	106(3)
F(1A)-C(2A)-C(1A)	110(2)
F(2A)-C(2A)-F(1A)	108(2)
F(2A)-C(2A)-C(1A)	112(2)
F(3A)-C(2A)-F(1A)	104(3)
F(3A)-C(2A)-F(2A)	110(3)
F(3A)-C(2A)-C(1A)	112(3)
O(3A)-C(3A)-C(4A)	111(3)
O(4A)-C(3A)-O(3A)	114(3)
O(4A)-C(3A)-C(4A)	135(3)
F(5A)-C(4A)-F(6A)	100(3)
F(5A)-C(4A)-F(4A)	103(3)
F(5A)-C(4A)-C(3A)	116(3)
F(6A)-C(4A)-C(3A)	115(2)
F(4A)-C(4A)-F(6A)	105(3)
F(4A)-C(4A)-C(3A)	116(2)
O(8A)-C(7A)-C(8A)	122(4)
O(7A)-C(7A)-O(8A)	129(4)
O(7A)-C(7A)-C(8A)	109(3)
F(12A)-C(8A)-F(10A)	107(3)
F(12A)-C(8A)-C(7A)	111(3)
F(11A)-C(8A)-F(12A)	108(3)
F(11A)-C(8A)-F(10A)	108(3)
F(11A)-C(8A)-C(7A)	114(3)
F(10A)-C(8A)-C(7A)	108(3)

Symmetry transformations used to generate equivalent atoms:

#1 -y+1,x-y,z   #2 -x+y+1,-x+1,z

Table 4. Anisotropic displacement parameters ($Å^2 x 10^3$) for **1**($(CF_3CO_2)_6$). The anisotropic displacement factor exponent takes the form: $-2\pi^2[h^2 a^{*2}U^{11} + ... + 2 h k a^* b^* U^{12}]$.

	U^{11}	U^{22}	U^{33}	U^{23}	U^{13}	U^{12}
C(5)	110(11)	76(10)	87(11)	11(8)	3(10)	49(10)
C(10)	110(12)	76(12)	111(13)	6(9)	2(10)	41(10)
C(9)	115(11)	67(11)	113(13)	12(9)	6(10)	35(9)
C(8)	119(11)	84(13)	136(15)	-19(12)	-2(12)	42(11)
C(7)	107(12)	64(11)	93(12)	20(8)	8(10)	34(10)
C(6)	100(11)	70(11)	87(12)	9(10)	-2(11)	35(10)
C(2)	118(11)	118(11)	90(12)	0	0	59(5)
C(4)	137(13)	87(13)	111(11)	7(13)	10(9)	74(13)
C(3)	113(10)	88(11)	87(9)	3(9)	-9(7)	53(10)
C(20)	179(13)	181(13)	265(16)	-113(12)	-30(11)	93(11)
C(22)	187(13)	211(16)	271(18)	-98(14)	-21(12)	110(13)
C(23)	199(15)	218(18)	320(20)	-115(15)	-32(14)	119(14)
C(24)	239(17)	253(18)	317(17)	-133(14)	-48(13)	141(15)
N(2)	227(14)	204(14)	303(14)	-144(12)	-57(11)	112(12)
C(21)	202(15)	200(15)	269(16)	-120(13)	-35(12)	107(13)
C(19)	160(11)	179(15)	231(17)	-86(14)	-24(11)	85(11)
C(18)	138(10)	93(8)	140(10)	-11(7)	7(9)	45(8)
C(32)	249(12)	189(9)	283(10)	-147(8)	-60(9)	106(9)
C(1)	93(12)	93(12)	85(12)	0	0	47(6)
C(13)	179(13)	181(13)	265(16)	-113(12)	-30(11)	93(11)

C(15)	187(13)	211(16)	271(18)	-98(14)	-21(12)	110(13)
C(16)	199(15)	218(18)	320(20)	-115(15)	-32(14)	119(14)
C(17)	239(17)	253(18)	317(17)	-133(14)	-48(13)	141(15)
N(1)	227(14)	204(14)	303(14)	-144(12)	-57(11)	112(12)
C(14)	202(15)	200(15)	269(16)	-120(13)	-35(12)	107(13)
C(12)	160(11)	179(15)	231(17)	-86(14)	-24(11)	85(11)
C(11)	138(10)	93(8)	140(10)	-11(7)	7(9)	45(8)
C(25)	249(12)	189(9)	283(10)	-147(8)	-60(9)	106(9)
C(26)	249(12)	189(9)	283(10)	-147(8)	-60(9)	106(9)
C(27)	249(12)	189(9)	283(10)	-147(8)	-60(9)	106(9)
C(28)	249(12)	189(9)	283(10)	-147(8)	-60(9)	106(9)
C(29)	249(12)	189(9)	283(10)	-147(8)	-60(9)	106(9)
C(30)	249(12)	189(9)	283(10)	-147(8)	-60(9)	106(9)
C(31)	249(12)	189(9)	283(10)	-147(8)	-60(9)	106(9)
F(7A)	236(13)	207(12)	329(15)	-13(12)	-39(14)	121(12)
F(9A)	236(13)	207(12)	329(15)	-13(12)	-39(14)	121(12)
F(8A)	236(13)	207(12)	329(15)	-13(12)	-39(14)	121(12)
O(5A)	236(13)	207(12)	329(15)	-13(12)	-39(14)	121(12)
O(6A)	236(13)	207(12)	329(15)	-13(12)	-39(14)	121(12)
C(5A)	236(13)	207(12)	329(15)	-13(12)	-39(14)	121(12)
C(6A)	236(13)	207(12)	329(15)	-13(12)	-39(14)	121(12)
F(1A)	236(13)	207(12)	329(15)	-13(12)	-39(14)	121(12)
F(2A)	236(13)	207(12)	329(15)	-13(12)	-39(14)	121(12)
F(3A)	236(13)	207(12)	329(15)	-13(12)	-39(14)	121(12)
O(1A)	236(13)	207(12)	329(15)	-13(12)	-39(14)	121(12)

O(2A)	236(13)	207(12)	329(15)	-13(12)	-39(14)	121(12)
C(1A)	236(13)	207(12)	329(15)	-13(12)	-39(14)	121(12)
C(2A)	236(13)	207(12)	329(15)	-13(12)	-39(14)	121(12)
F(5A)	270(30)	330(40)	310(30)	10(20)	40(20)	90(30)
F(6A)	310(30)	260(30)	290(30)	20(20)	80(20)	120(20)
F(4A)	310(30)	270(30)	290(30)	10(20)	60(30)	140(30)
O(3A)	290(30)	260(30)	130(20)	-60(20)	0(20)	100(20)
O(4A)	310(40)	240(30)	270(40)	30(30)	80(30)	110(30)
C(3A)	290(30)	270(30)	250(40)	-20(20)	40(30)	120(20)
C(4A)	300(30)	270(30)	290(30)	-10(20)	60(20)	120(20)
F(12A)	310(50)	270(40)	260(50)	-60(30)	40(40)	120(40)
F(11A)	340(50)	250(40)	250(40)	-30(40)	50(30)	110(40)
F(10A)	330(50)	250(40)	220(40)	-40(30)	50(30)	110(40)
O(8A)	280(70)	330(70)	220(40)	-40(40)	70(40)	150(60)
O(7A)	110(30)	150(40)	210(40)	-50(30)	60(30)	-30(30)
C(7A)	290(50)	270(40)	220(40)	-40(30)	70(30)	120(40)
C(8A)	330(50)	270(40)	230(40)	-40(30)	60(30)	120(40)

Table 5. Hydrogen coordinates (x 10^4) and isotropic displacement parameters ($Å^2$x 10^3) for Badjic_PK20230327.

	x	y	z	U(eq)
H(10)	5095	2055	5160	122
H(7)	6957	2066	5195	110
H(4A)	5262	2270	6999	155
H(4B)	5120	2807	6769	155
H(4C)	5586	2895	7557	155
H(22)	3510	731	3955	262
H(23)	2557	-78	3509	289
H(24)	2505	-800	2478	314
H(21)	4359	96	2340	265
H(32A)	3026	-1136	1391	290
H(32B)	3324	-467	926	290
H(1A)	6863	3756	7918	136
H(1B)	6893	3137	7918	136
H(1C)	6244	3107	7918	136
H(15)	6980	338	4272	262
H(16)	6890	-555	3648	289
H(17)	6342	-948	2321	314
H(14)	5974	443	2242	265
H(25A)	5927	-358	818	290

H(25B)	5629	-1008	1305	290
H(27)	5273	-11	350	290
H(28)	4323	-56	385	290
H(30)	3788	-1270	2428	290
H(31)	4738	-1225	2392	290

Cell Studies

Materials. HEK-293 and HepG2 cell lines have been purchased from ATCC (American Type Culture Center). DMEM media, FBS, PBS, 0.25% Trypsin-EDTA solution, and Penicillin-Streptomycin were all purchased from Sigma-Aldrich. Tissue-culture flasks and clear-bottom 96-well plates have been purchased from VWR International. CellTiter 96® AQueous One Solution Cell Proliferation Assay (MTS Assay Kit) has been purchased from Promega.

Procedure

Cell culture. Cytotoxicity assay was performed using the human embryonic kidney (HEK-293) and human liver cancer (Hep-G2) cell lines, obtained from American Type Culture Collection (ATCC, Manassas, GA). HEK-293 and HepG2 cells were cultured in DMEM media supplemented with 10% FBS and 1% Penicillin-Streptomycin in an incubator at 37°C and at 5% CO_2. Cells were sub-cultured every 2-3 days using 0.25% Trypsin-EDTA solution.

Cell viability assay (MTS Assay).

Day 0

100 µL of cell culture media containing cells were seeded in a clear-bottom 96-well plate at a density of 1 x 10^4 cells/well. Cells were then allowed to adhere overnight.

Day 1

Cell culture media was then discarded, and cells were washed with PBS (x 2). 100 µL of compounds **1** and **2** were dissolved in cell culture media and were then added to HEK-293 cells and HepG2 cells at three different concentrations (100 µM, 50 µM, and 25 µM). Cells were also incubated with 100 µL of PBS alone and 100 µL of only cell culture media (without sample) to serve as controls. Viability is reported as the mean of three total wells per data point, and error bars represent +/- standard deviation.

Day 3

After an incubation period of 48 h, the cell culture media was removed, and cells were washed with PBS (x 2). 20 µL of MTS reagent with 100 µL of cell culture media was added to each well from Day 1 and to an additional three wells to serve as blank. Samples were then incubated for 3 h at 37°C and 5% CO_2. Absorbance was then recorded at 490 nm using SpectraMax, M5 Plate reader (Molecular Devices).
